# Current use of medicinal plants for children’s diseases among mothers in Southern Romania

**DOI:** 10.3389/fphar.2024.1377341

**Published:** 2024-05-22

**Authors:** Madalina Petran, Dorin Dragoș, Irina Stoian, Adelina Vlad, Marilena Gilca

**Affiliations:** ^1^ Department of Functional Sciences I/Biochemistry, Faculty of Medicine, Carol Davila University of Medicine and Pharmacy, Bucharest, Romania; ^2^ Department of Medical Semiology, Faculty of Medicine, Carol Davila University of Medicine and Pharmacy, Bucharest, Romania; ^3^ First Internal Medicine Clinic, University Emergency Hospital Bucharest, Carol Davila University of Medicine and Pharmacy, Bucharest, Romania; ^4^ Department of Functional Sciences I/Physiology, Faculty of Medicine, Carol Davila University of Medicine and Pharmacy, Bucharest, Romania

**Keywords:** children, complementary treatment, medicinal plants, pediatrics, Romania

## Abstract

There is a limited number of studies focusing on ethnomedical practices in children, particularly in Eastern Europe. Romania has a rich history of using medicinal plants in ethnopediatric care, and our objective was to identify the medicinal plants currently employed in treating childhood illnesses in the southern region of the country.

**Material and methods** Our investigation used structured interviews, focusing on respondent demographics, local names of therapeutically employed herbs, the specific plant part(s) utilized, methods of preparation and administration, and local folk indications of taxa. A total of 326 mothers with children aged 0 to 18, hospitalized in the “Grigore Alexandrescu” Children Emergency Hospital Bucharest and residing in Southern Romania, were enrolled in the study. Use Value Citation Index (UV*c*), Informant Consensus Factor (Fic), and Fidelity Level (FL) were calculated.

**Results** Twenty-five plants were identified for treating children’s diseases in Southern Romania. The majority of informants resided in urban areas, and mothers primarily acquired knowledge from family members and healthcare professionals. The herbs most frequently employed were *Mentha* spp. (UV = 0.509) for diarrhea, *Matricaria spp*. (UV = 0.301) for skin infections (Fic = 0.99) and digestive diseases (Fic = 0.98), and *Calendula officinalis* L. (UV = 0.365) for skin diseases (Fic = 0.99). Less utilized were *Raphanus raphanistrum subsp. sativus* (L.) Domin in respiratory diseases, *Prunus avium* (L.) L. stalks in urinary tract ailments, *Helianthus annuus* L. in ear infections, *Allium sativum* L. in intestinal parasitosis, *Viola tricolor* L. in hives, *Triticum aestivum* L. in dermatitis and *Allium ursinum* L. as a tonic. In 184 cases herbal treatment was used in conjunction with conventional medications. Education level correlated with the number of employed plants and the variety of treated ailments, while residency (rural vs. urban) did not. Both residency and education influenced plant procurement methods: rural background and, surprisingly, higher education were linked to a preference for harvesting rather than purchasing plants.

**Conclusion** Botanical remedies are still commonly used in the treatment of pediatric diseases in Southern Romania, although the variety of taxa seems reduced compared to the past. Further exploration is essential to unlock the maximum benefits of ethnopediatric practices.

## 1 Introduction

Traditional medicine, consisting of a multitude of health-related practices, approaches, knowledge, and beliefs, is an invaluable medical and cultural heritage for humankind. According to the World Health Organization, traditional medicine incorporates plants, animal and mineral-based medicines, spiritual therapies, manual techniques, and exercises (Burton et al., 2015). Among them, herbs represent the main remedies used over the millennia for preventing and treating a large variety of ailments. Since ancient times, people have prepared their own herbal medicines or acquired them from the local traditional healers (Che et al., 2017). It is highly suggestive that about 80% of the populations in developing countries still use herbal medicine to meet their primary healthcare requirements (Zhang et al., 2019). Moreover, in the past few decades, people have been rediscovering more and more traditional, and predominantly herbal medicine (Murgia et al., 2021), either as an alternative to or associated therapy with modern drugs (Santucci et al., 2021)

In traditional medicine, the knowledge is transmitted mainly orally, emphasizing the importance of documentation (Bruhn and Rivier, 2019) to prevent rapid loss. Despite women’s contributions to the History of Science often being overlooked or marginalized (Mariath and Baratto, 2023), an increasing number of studies point out the essential role women have played in preserving traditional medical practices over time. From ancient Greek medicine to early modern European herbalism, botanical local knowledge was often held by (more or less) anonymous female herbalists (Bowcutt and Caulkins, 2020). During the medieval period, lettered “middle class and elite” women were often educated in the “domestic arts,” encompassing medical subjects and everyday healing based on herbal remedies. Given their significant role in infant care and health, women were trained as empirical practitioners to provide primary medical assistance for children’s ailments (Allen, 2016; Soares, 2023).

The recent literature on traditional medicine is mainly focused on the knowledge of experienced practitioners or healers, whereas domestic medicine research focused on the users knowledge, such as the skills and cultural beliefs of women, is rather scarce (Choudhry, 1997; Cabré, 2008), although it is well documented that women and elders are the most active consumers of traditional medicine (Voeks, 2007; Schunko et al., 2012; Jimenez-Fernandez et al., 2023). Women were also related to collecting and drying herbs as a source of botanical drugs (Ishtiaq et al., 2022), as well as to plant preservation efforts (Bowcutt and Caulkins, 2020). Additionally, household strategies using a wide variety of plants and plant parts such as leaves, bark, roots, fruits, seeds, or animal elements like honey, eggs, and fat that are practiced by pauper women have been recognized for yielding satisfactory results. These strategies have successfully coexisted with conventional therapies (Mandu and Silva, 2000). Exploring the contemporary ethnomedical knowledge and practices of mothers holds promise for advancing ethnopediatric research.

Despite the impressive scientific achievements in pediatrics, an unacceptably high number of children suffer from life-threatening conditions (e.g., wasting) or die every year from preventable causes (e.g., acute respiratory infections, diarrhea) (“UNICEF Annual Report, 2021: Protecting Child Rights in a Time of Crises,” 2021). For instance, in 2020, 5 million children under the age of 5 lost their lives (equivalent to13,800 children every day), mostly due to the inaccessibility of quality healthcare, affordable treatments, and proper food and water (“UNICEF Annual Report 2021: Protecting Child Rights in a Time of Crises,” 2021). The wealth of traditional pediatric knowledge remains underexplored and undervalued, yet it holds the potential to provide accessible therapeutic solutions and inspire scientists in the development of new pharmacological agents. For example, a systematic review suggested that herbal medications can be effective for mild conditions, such as diarrhea, dehydration, and infantile colic (Anheyer et al., 2017).

Unfortunately, there is a relative scarcity of studies that specifically approach the subject of ethnomedical practices in children nowadays, especially in Eastern Europe. We found a few studies evaluating pediatric ethnopharmacological knowledge in Africa (Geissler et al., 2002; Nalumansi et al., 2014; Abubakar et al., 2017; Tugume and Nyakoojo, 2019), South America (Ruysschaert et al., 2009) and Asia (Mahomoodally and Sreekeesoon, 2014). Nevertheless, there is an increasing number of general ethnopharmacological studies in the Balkan area (Serbia, Bulgaria, Montenegro, Albania, Macedonia, etc.), which may provide insight into the pediatric potential of local plants for diseases common to both adults and children or human nutrition. For instance, these studies report the traditional use of plants for wound healing and skin related-problems (Jaric et al., 2018; Tsioutsiou et al., 2022), respiratory, gastrointestinal, urogenital, and cardiovascular diseases (Ivancheva and Stantcheva, 2000; Menković et al., 2011; Šavikin et al., 2013; Zlatkovic et al., 2014; Mustafa et al., 2015; Lumpert and Kreft, 2017; Janaćković et al., 2022; Gradinaru, 2023; Marković et al., 2023; Jarić et al., 2024), as well as the use of edible plants (Redžić and Ferrier, 2014; Dogan et al., 2015).

Romanian people have a valuable ethnomedical and ethnobotanical heritage kept alive through oral transmission, within families of healers, midwives, herb collectors, and monastic communities (Barca and Strachinaru, 1974; Butura, 1979; Pavelescu, 2004; Marian, 2010; 2008; Pop et al., 2010; Drăgulescu, 2013; Mărculescu and Olteanu, 2014; Dragulescu, 2019; 2017; Mattalia et al., 2020). Researchers evaluating the monographs of medicinal plants included in various editions of the Romanian Pharmacopeia (RPh) published between 1862–1993 (RPh I 1862; RPh II 1874; RPh III 1893; RPh IV 1926; RPh V 1943; RPh VI 1948; RPh VII 1956; RPh VIII 1965; RPh IX 1976; RPh X 1993) found that over time, 289 medicinal plants were included, with the majority (176, i.e. 60.9%) originating from Romanian ethnomedicine (Mărculescu et al., 2004; Benedec et al., 2017). The first edition of the RPh, consisting of 790 pages and written at the initiative of Dr. Carol Davila, was recognized as one of the most appreciated pharmacopeia in Eastern Europe, with its value corresponding to the scientific standards of that historical period (Besciu et al., 2011; Benedec et al., 2017). Out of the 301 drugs included in RPh I, 207 were of herbal origin (Stancu et al., 2014).

In a previous historical analysis focused on Romanian folk pediatrics practiced during 1860s–1970s, we found 153 medicinal plants with ethnotherapeutic significance for children’s diseases (Petran et al., 2020), but the level of their contemporary use was not yet explored. Considering the central role of women as household caregivers, this study aimed to evaluate the current ethnopediatric knowledge and domestic use of medicinal plants by mothers for treating their children in Southern Romania.

## 2 Materials and method

### 2.1 Background of study area

Romania is recognized as one of the most biogeographically diverse countries of the European Union, having Alpine, Continental, Pannonic, Pontic, and Steppic regions. These specific geographic features combined with its modified temperate continental climate provide the conditions for a diverse flora (3,829 vascular and 979 non-vascular spontaneous plant taxa) (Hurdu et al., 2022), also characterized by a great abundance of medicinal and aromatic plants. A recent evaluation concluded that in Romania there are more than 750 species of plants with medicinal properties (Dihoru and Boruz, 2014).

**FIGURE 1:**
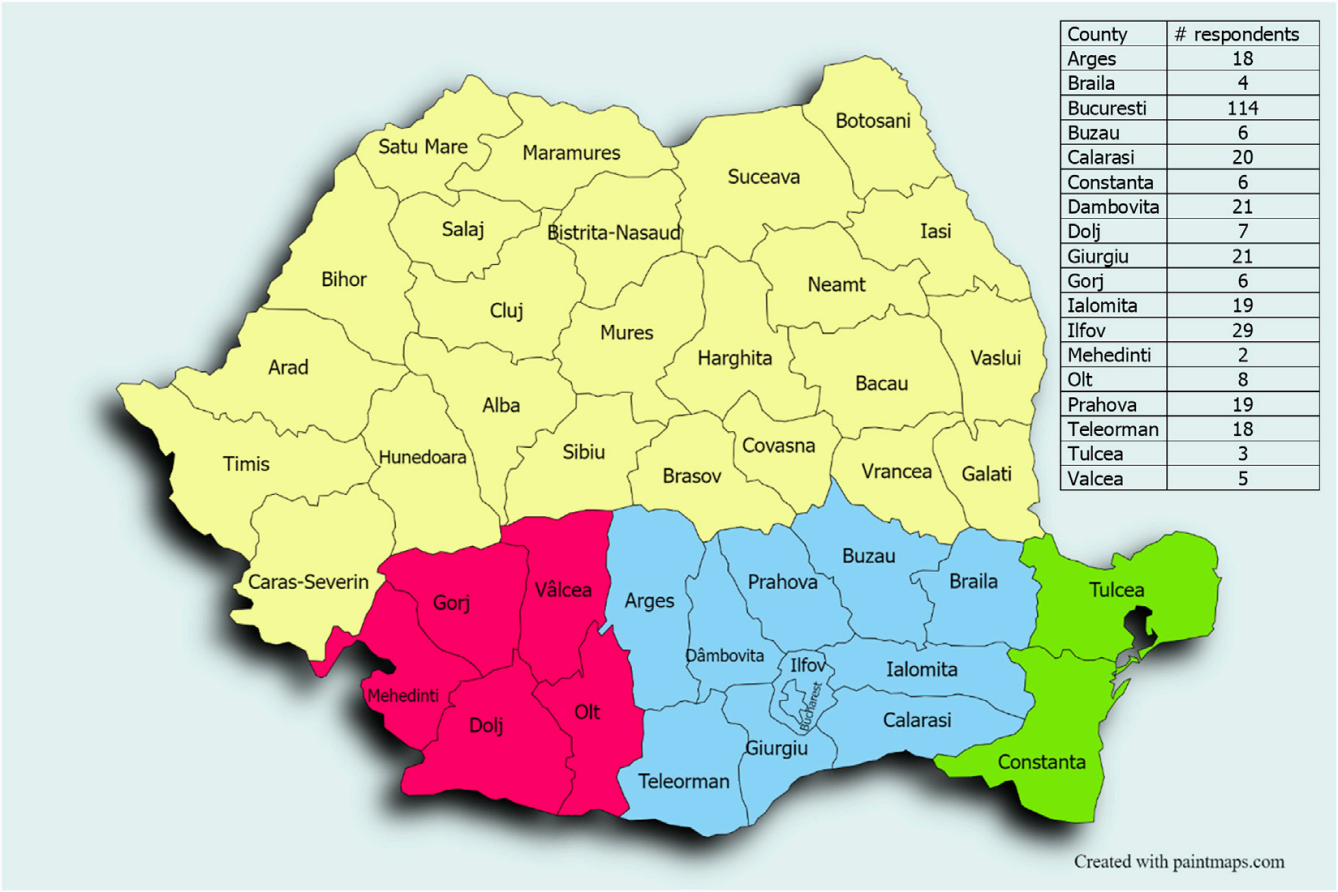
Map of Romania indicating the study area, which spans three main regions: Muntenia (blue), Oltenia (red), and Northern Dobruja (green), along with the distribution of respondents by counties (top right).

Southern Romania includes two regions, known historically as Walachia and Northern Dobruja (Figure 1). Walachia covers two historical regions, Muntenia (Greater Wallachia) and Oltenia (Lesser Wallachia), and has about 10,784,874 million people in an area of 101,248 km^2^. It is worth mentioning that the South-Eastern Carpathians alone, partially belonging to Southern Romania, represent one of the major biodiversity hotspots in Europe (Bálint et al., 2011; Dihoru and Boruz, 2014; Hurdu et al., 2022).

Near this area, in Northern Dobruja, there is the Danube Delta, a World Heritage (https://whc.unesco.org/en/list/588/). Danube Delta represents a unique biosphere reserve and wetland ecosystem, quoted as the best-preserved delta in Europe, and the third largest biodiversity area in the world, with over 5500 flora and fauna species (Ciocarlan, 1994; Claudino-Sales, 2019).

### 2.2 Methods

A survey instrument was developed by a panel of researchers with a medical background and extensive knowledge in phytotherapy and ethnomedicine, in accordance with other studies related to analogous issues (Kennedy, 2005; Raal et al., 2013; Wu et al., 2013). The study population encompassed mothers with a domicile in Southern Romania whose children fell in the age category 0–18 years and was conducted between September 2019 and December 2022 in “Grigore Alexandrescu” Children Emergency Hospital, a tertiary hospital in Bucharest, Romania. The project was approved by the Ethics Commission of ‘Grigore Alexandrescu’ Children Emergency Hospital (no. 3/1736). Ethnobotanical and ethnomedical investigations were carried out following the ethical guidelines outlined by the International Society of Ethnobiology (ISE) Code of Ethics (The ISE code of ethics, International Society of Ethnobiology, 2006).

Data collection was based on structured interviews (Etkin, 1993; Heinrich et al., 2009) applied to mothers of children hospitalized for various diseases. Responses were recorded by a health professional with expertise in phytotherapy and ethnomedicine. The questionnaire was strictly confidential, anonymized, and non-compulsory. The purpose, methodology, and objective of the study were explained to all participants, and written informed consent was obtained subsequently. The interviews occurred in an individual manner and took between 20 and 30 min to complete.

The questionnaire comprises structured open-ended and close-ended questions regarding the medicinal plant species used, their indications and ethnopharmacological activities*,* sources of knowledge, plant part(s) used, type of preparation, routes of administration, concomitant use of conventional drugs, and treatment outcomes (for further details, please refer to the Table 1, Supplementary Material). The demographic data of each participant (age, gender, area of residence, level of education) were recorded, together with the age when the children first received the herbal remedy. Standardized ethnobotanical procedures that confer transparency (e.g., availability of information on the frequency of citations), important to assess the relative cultural rank of a species, were followed (Heinrich et al., 2009). Since the present study was essentially a survey conducted within a hospital unit, the collection of voucher specimens was not feasible. The specific study design resembled that of other studies, such as surveys evaluating the use of herbal remedies by pharmacy customers (Knotek et al., 2012; Raal et al., 2013; Zeni et al., 2017) or by members of various minorities (Bhamra et al., 2017). The assessment of herbal authenticity was not within the scope of this study. The accuracy of the taxonomy and nomenclature of vascular plants was verified by cross-referencing with World Flora Online (WFO) (www.worldfora online.org).

**TABLE 1:** Multivariate analysis.

Independent parameters	Estimate	Std.Error	t_value	p-value
Used plant count–1st step				
Age	0.008564	0.008851	0.967	0.33
Urban residency	0.151991	0.162835	0.933	0.35
Higher educational level	0.941605	0.197033	4.779	3e-06
Purchased	−0.265285	0.202679	−1.309	0.19
Used plant count–2nd step				
Age	0.008353	0.008847	0.944	0.35
Higher educational level	0.964837	0.195416	4.937	1e-06
Purchased	−0.226657	0.198369	−1.143	0.25
Used plant count–3rd step				
Higher educational level	1.0090	0.1897	5.319	2e-07
Purchased	−0.2299	0.1983	−1.159	0.25
Ailments treated count–1st step				
Age	0.010236	0.007954	1.287	0.2
Urban residency	0.161682	0.146323	1.105	0.27
Higher educational level	0.632904	0.177053	3.575	0.0004
Purchased	−0.322763	0.182127	−1.772	0.077
Ailments treated count–2nd step				
Age	0.010012	0.007954	1.259	0.21
Higher educational level	0.657617	0.175695	3.743	0.00022
Purchased	−0.281673	0.178351	−1.579	0.12
Ailments treated count–3rd step				
Higher educational level	0.7105	0.1707	4.161	4e-05
Purchased	−0.2855	0.1785	−1.600	0.11
Procurement by purchase–1st step				
Age	−0.008062	0.017718	−0.455	0.65
Urban residency	1.239028	0.350530	3.535	0.0004
Higher educational level	−2.057001	0.615726	−3.341	0.0008
Procurement by purchase#–2nd step				
Urban residency	1.2334	0.3501	3.523	0.0004
Higher educational level	−2.0870	0.6124	−3.408	0.00065

Legend: The dependent variables (Used plant count, Ailments treated count, Procurement by purchase) are bold-typed as are the designations of the various steps of the MVA, for each of these dependent variables The second column contains the estimate, which is the average change in the log odds of the dependent variable associated with a one unit increase in each independent variable.

Following the collection and analysis of the questionnaires, we compiled a list of medicinal plants based on informant input. To assess the conformity and/or novelty of our results, we conducted a systematic search of the ethnomedical or bioscientific data on the identified medicinal plants. This comprehensive search was carried out across PubMed and Google Scholar databases. Our strategy involved using specific phrases such as: [(Latin name of the plant) OR (vernacular name of the plant)] AND (children OR toddler OR pediatrics OR adults OR indication OR biological activity). For example, a search query could include (Foeniculum vulgare OR fennel) AND (children OR toddler OR pediatrics OR adults OR indication OR biological activity). This method enabled us to analyze existing literature and evaluate the medicinal properties and potential applications of the identified plants within the age groups of interest across various medical contexts, and in adulthood, specifically in the pathologic spectrum reported by our informants.

### 2.3 Data analysis

In this article, the terms “variable” and “parameter” will be considered equivalent and used interchangeably. The same holds for the terms “association” and “correlation.” The statistical analysis consisted of both descriptive and inferential statistics. Descriptive statistics included the calculation of the median and interquartile range (from the first to the third quartile) to characterize numerical parameters such as mother age, used plants count, treated ailments count, and child age when the first herbal treatment was given.

Based on the number of plants used, the mothers were divided into two groups: one comprising those who utilized one or two plants (*n* = 191), and the other consisting of those who employed more than two plants (*n* = 135). Similarly, concerning the number of treated ailments, two groups of informants were defined: one including those who used plants for treating one or two ailments in their children (*n* = 202), and the other, those who treated more than two diseases (*n* = 124).

Shapiro-Wilk test was used to assess the normality of data distribution. Inferential statistics encompassed univariate analysis (UVA) and multivariate analysis (MVA). UVA included: 1. regression by Spearman’s method, used to estimate the correlation between two numerical parameters 2. Mann-Whitney (Wilcoxon) test with continuity correction for exploring the correlation of various numerical parameters with binary (i.e., with only two possible values) categorical parameters 3. Kruskal-Wallis test, employed to evaluate the correlation of numerical parameters with multivalent (i.e., with more than two possible values) categorical parameters 4. Fisher’s exact test is used to explore the correlations between various categorical parameters. In particular, Fisher’s exact test was utilized to determine whether there is an association between the social background (rural vs. urban) and the employment of medicinal plants. The strength of the association was estimated by odds ratio. The accompanying 95% confidence intervals for odds ratio are an estimation of the real value in the population: there is a 95% probability that the real value in the population falls within the confidence interval. Of course, it is difficult to decide whether the mothers in this tertiary care pediatric hospital are a representative sample of the entire population of mothers.

When multiple comparisons were performed by Mann-Whitney test, the significance level (commonly set at 0.05) was lowered according to Bonferroni correction: the corrected significance level was determined by dividing 0.05 by the number of comparisons. In cases where Kruskal-Wallis test yielded a significant result, pairwise Wilcoxon tests were subsequently conducted using Benjamini-Hochberg procedure to reduce the false discovery rate.

After having established by UVA the variables significantly associated with used plants count, treated ailments count, and purchase method, MVA was performed to determine the variables independently associated with each of these parameters. The method employed for MVA was backward stepwise regression. For each dependent variable, regression was first performed on all n (supposedly) independent variables pointed out by UVA as being statistically significantly associated with the dependent variable. Among the variables proven to lack a statistically significant (*p* > 0.05) contribution, the one with the smallest contribution (which was also the variable with the smallest t_value) was eliminated. Subsequently, regression was performed again on the remaining n - 1 variables, and once more, the variable with the smallest contribution was eliminated. This process continued iteratively until all remaining variables were statistically significantly associated with the dependent variable. The steps of the MVA for all three dependent variables are shown in Table 1.

All statistical calculations and resulting graphical representations were performed using R language and environment for statistical computing and graphics (version 4.2.3.). The employed packages were dplyr, tidyverse, ggplot2, ggpubr, mosaic, e1071, and mediation.

We divided the data regarding indications into use categories of diseases (Table 2), according to the International Classification of Primary Care (ICPC)(WHO Second edition ICPC-2, 2012), which was adapted to fit the ethnomedical reality (Staub et al., 2015).

**TABLE 2:** Classification of ethnopediatric indications included in the present study, adapted from the International Classification of Primary Care (ICPC).

Body system	Example of disease
General	Weakness, Allergies, Anemia
Musculoskeletal	Trauma
Neuropsychological	Agitation, Sleep disturbances/Insomnia/Nightmares/Weeping during sleep
Respiratory	Acute respiratory diseases, Asthma, Bronchitis, Phlegm in the throat, Cold, Cough, Ear pain
Digestive	Colic, Abdominal cramps, Acute digestive infections, Diarrhea, Flatulence, Intestinal worms, Intestinal parasites
Skin	Burn wounds/Burns, Dermatitis, Diaper (napkin) dermatitis, Eczema, Skin inflammation, Skin infections, Skin lesions, Verruca, Wounds, Eye infections
Urinary	Urinary tract infections, Urolithiasis

The collected data were analyzed using quantitative methods. Use-Value Citation Index, Fidelity Level, and Informant Consensus Factor were calculated (Friedman et al., 1986; Phillips and Gentry, 1993; Hoffman and Gallaher, 2007; Heinrich et al., 2009; Hajdari et al., 2018; Trotter and Logan, 2019).

The Use-value citation index (UV_c_) evaluates the relative importance of each species based on its cited uses, and it is calculated for all taxa with the formula:

$${\text{UV}}_{c}=\left(\sum U_{\text{is}}\right)/N,$$

where U_is_ is the sum of the total number of all individual use citation reports concerning a given taxa, divided by the total number of informants (N) (Phillips and Gentry, 1993; Hoffman and Gallaher, 2007).

The fidelity level (FL) represents the percentage of informants reporting the usage of a certain medicinal plant for the same major indication (Friedman et al., 1986) and was calculated to determine the most important species used to treat the most frequently reported diseases or ailments.

$$\text{FL }\left(\%\right)=Np/N\times 100,$$

where *Np* is the number of informants reporting the usage of a plant species to treat a particular disease, and *N* denotes the total number of informants utilizing the plant as a treatment for any given disease (Friedman et al., 1986; Mahomoodally and Sreekeesoon, 2014).

The categories selected for the Informant Consensus Factor (F_ic_) analysis are outlined in Table 3. Each taxon’s usage was assigned to the relevant category before analysis, employing the following formula:

$$F_{\text{ic}}=\left(N_{\text{uc}}‐N_{t}\right)/\left(N_{\text{uc}}‐1\right),$$

where N_uc_ is the total number of use citations in each category and N_t_ is the number of taxa used in that category. F_ic_ estimates user consensus regarding the medicinal plants. The value of this factor ranges from 0 to 1. High F_ic_ values (close to 1.0) are obtained when relatively few species are reported to be used by a large proportion of informants for a particular nosological category, whereas lower F_ic_ values indicate that informants disagree upon the taxa to be used in the treatment within a certain category.

**TABLE 3:** Informant consensus factor.

Disease category	N_t_	N_ur_	F_ic_
Respiratory diseases	8	169	0.96
Digestive diseases	9	361	0.98
Urinary tract diseases	1	2	1
Skin conditions	4	253	0.99
Nervous system	1	3	1
Trauma/musculoskeletal	1	87	1
General: weakness, allergies, anemia	5	68	0.94

Legend. F_ic_ - Informant Consensus Factor; N_t_ -number of taxa used in that category; N_ur_-total number of use citations in each category.

Binomial distribution was used to calculate the confidence limits for the various percentages yielded by our study sample.

## 3 Results

### 3.1 Demographic characteristics of participants

**FIGURE 2:**
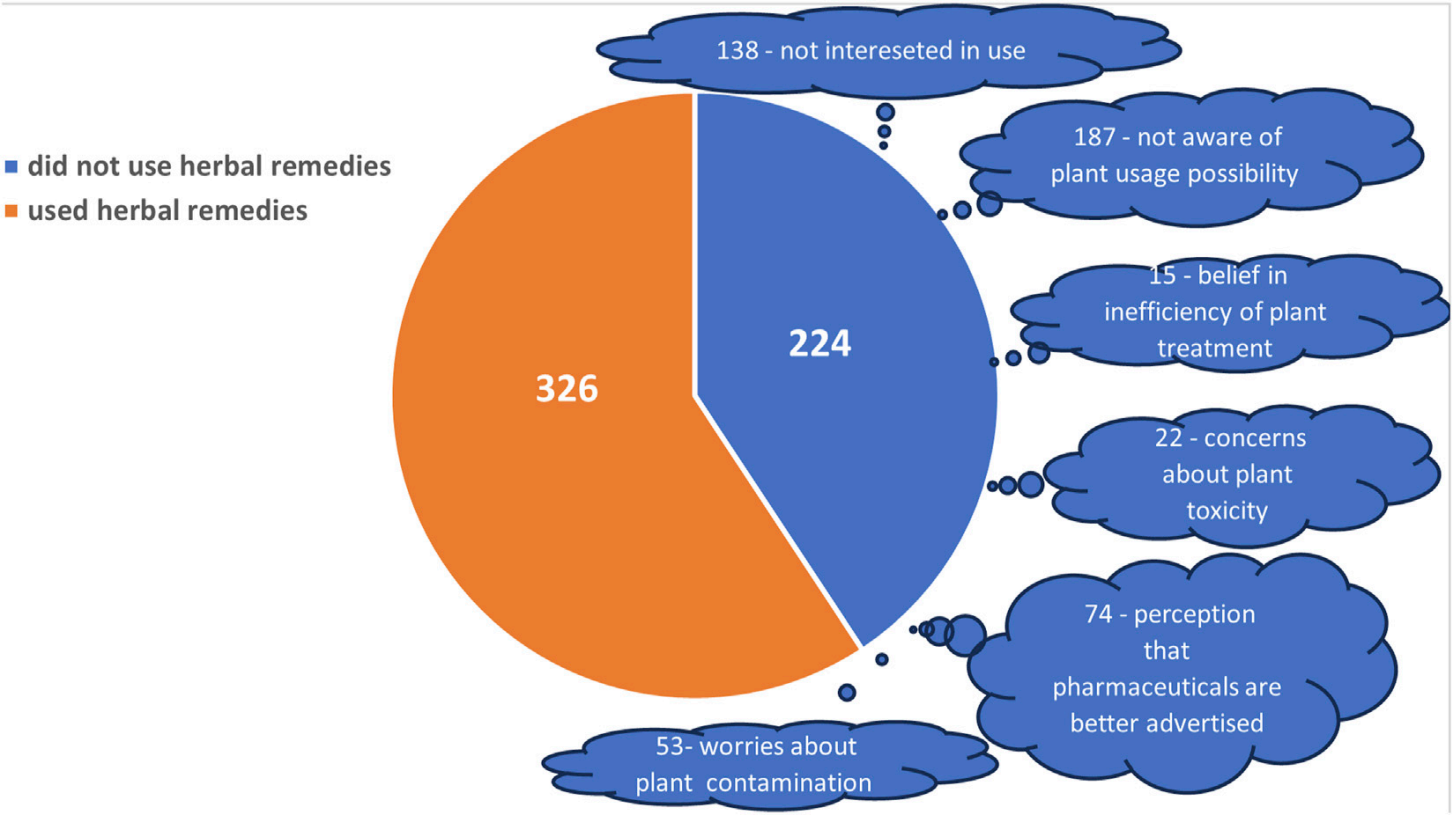
Selection of informants for survey participation, with each category representing the number of informants. 326 informants declared the usage of herbal remedies and were included in the study, whereas 224 did not, for reasons depicted in the figure.

A total of 550 mothers living in Southern Romania were initially interviewed with a median age of 37 (interquartile range (IQR) of 31–42 and a skewness of −0.2194345). Among them, 224 respondents (40.72%, 95% confidence limits (95% CL): 38.63–42.82) did not utilise herbal remedies based on raw-plant material in treating their children (Figure 2). Reasons for this included: not being aware of this option (187 mothers, among whom 138 were nonetheless interested in using plants to treat their children), having learned from media that plant treatment is not useful or may even be toxic (22 mothers), and considering herbal remedies as inefficient (15 mothers).

Additionally, 74 informants indicated advertising of the pharmaceutic products as one of the factors influencing their decisions. Concerns were raised by 53 informants about plant contamination by pollutants. They declared that due to the lack of time and opportunity to harvest plants of trusted quality, they used pharmaceutical agents based on plant extracts, but mixed with synthetic compounds, vitamins, and minerals that are generally recommended by physicians, pharmacists, friends, and in the media. Due to the mixed nature (herbal and non-herbal/natural and synthetic) of the medicines used, we decided to exclude them from enrollment in the study.

The employment of medicinal plants was declared by 177 and denied by 76 out of 253 mothers living in rural communities. Out of 297 mothers living in urban areas, 149 have confirmed the employment of medicinal plants, while 148 have denied it. Fisher exact test yields a less than 0.00001 *p*-value, indicating a strong association between social background and the utilization of medicinal herbs. Indeed, mothers of rural background have a higher propensity to use medicinal plants compared to mothers of urban background, as reflected by the odds ratio of 2.31 with a 95% confidence interval of 1.63–3.29.

The remaining 326 mothers (59.27%, CL95%: 57.18%–61.37%), who declared the use of medicinal plants, used plant treatment based on raw vegetal material (self-harvested or bought) for their children’s illnesses and were included in the study (Figure 2).

These herbal therapy users originated from 14 counties of Southern Romania: Argeș (18), Braila (4), Bucuresti (114), Buzau (6), Calarasi (20), Constanta (6), Dambovita (21), Dolj (7), Giurgiu (21), Gorj (6), Ialomita (19), Ilfov (29), Mehedinti (2), Olt (8), Prahova (19), Teleorman (18), Tulcea (3), Valcea (5) (Figure 1).

Approximately one-third (37%) of the participants resided in rural areas, while the remaining majority (63%) lived in urban areas. In terms of age distribution, 97% of the mothers were above 18 years old, with a significant majority falling into the 35–39 and 40–44 age groups. Regarding educational attainment, 4% of the respondents had no formal education, 18% of them had completed primary education, 57% had attained secondary-level education, and 21% held a college degree (Table 4).

**TABLE 4:** Demographic characteristics of the herbal therapy users.

Characteristic	Description	Percentage (%)	95 (%) CL
Age category	14–18	3	2.06–3.94
	18–24	7.4	5.95–8.85
	25–29	14.3	12.36–16.24
	30–34	11.2	9.45–12.95
	35–39	27.3	24.83–29.77
	40–44	23.6	21.25–25.95
	>45	13.2	11.33–15.07
Residence	Urban	37	34.33–39.67
	Rural	63	60.33–65.67
Religion	Christian	100	
Level of education	No education	4	2.91–5.09
	Low/primary level	18	15.87–20.13
	Medium level	57	54.26–59.74
	College degree	21	18.74–23.26

Note. The 95% confidence limits (95% CL) are calculated relative to the population of herbal treatment employing women.

**FIGURE 3:**
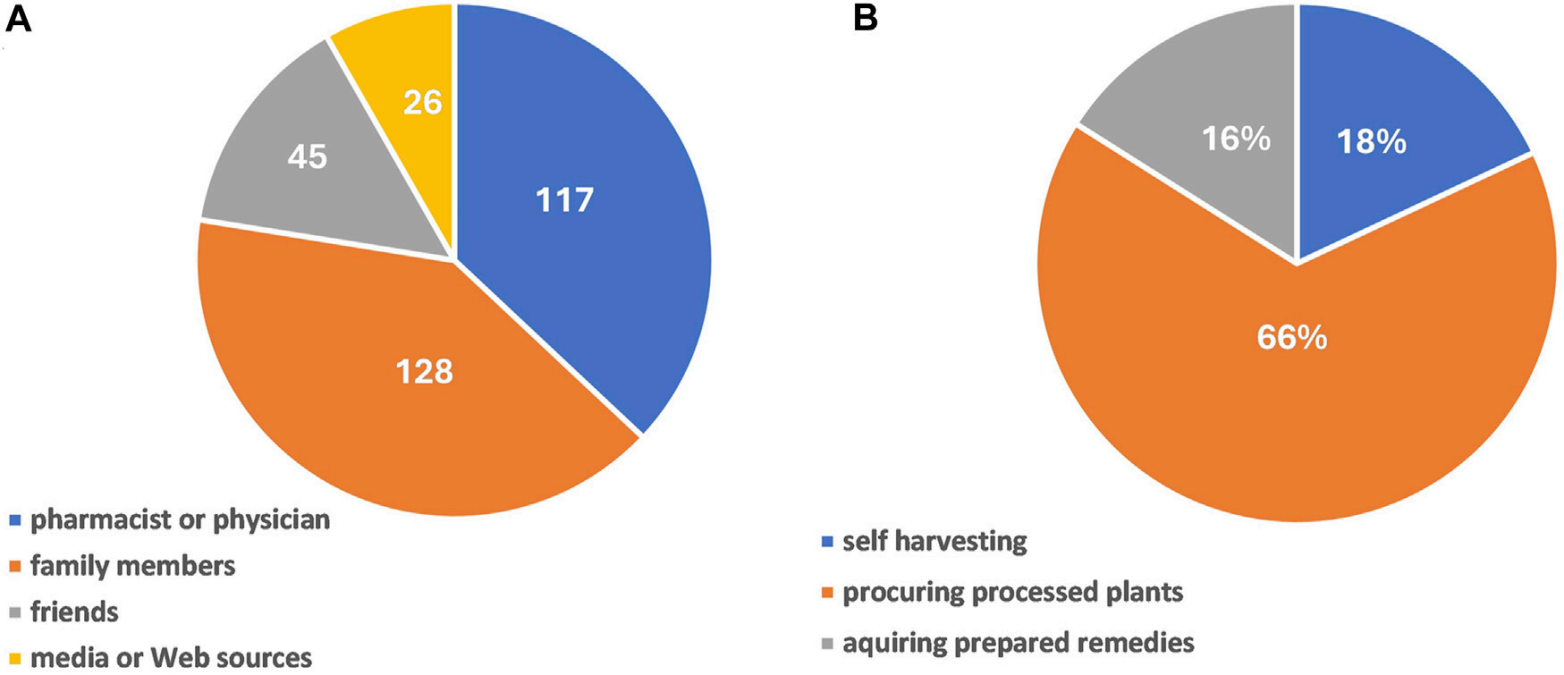
Recommendation sources for medicinal plant usage (with each category representing the number of informants) **(A)**, and the sourcing of the botanical remedies documented in our survey (with each category representing the percentage of informants) **(B)**.

From this point on, the 95% confidence limits were calculated relative to the population of herbal treatment employing women. In 117 cases (35.89%, 95% CL: 33.23%–38.55%) the treatment was recommended by a pharmacist or a physician, in 128 cases (39.26%, 95% CL: 36.56%–41.96%) by family members, in 45 cases (13.80%, 95% CL: 11.89%–15.71%) by friends, and in 36 cases (11.04%, 95% CL: 9.3%–12.78%) the information was found in media or on the Web (Figure 3A). Interestingly, more than one-third of the informants (40.49%, 95% CL: 37.77%–43.21%) stated that herbal remedies were the only treatment used, whereas the others (59.51%, 95% CL: 56.79%–62.23%) associated plants with allopathic drugs. In 196 of the cases, children were given both raw plant-based remedies and pharmaceutical drugs based on plant extracts.

An impressive 88% (95% CL: 86.2%–89.8%) of the informants reported an improvement in the disease course following herbal therapies and consequently declared their intention to use and recommend the use of plant-based remedies in the future.

### 3.2 Diversity of plant species used for pediatric treatment

The collected data revealed that a total of 25 medicinal plant species from 15 families were used to treat pediatric diseases. Plant indications and activities, parts used, way of administration, UVc, FL, and targeted body systems are provided in Table 5.

**TABLE 5:** The medicinal plants recorded in the study, with their corresponding therapeutic use, route of administration, mode or preparation, use value citation index, and fidelity level.

Nr	Latin name	Family name	English name	Romanian name	Origin	Indications and targeted body systems (nr. of mentions and FL)	Ethnopharmacological activities	Parts used	Route of administration	Preparation mode	Body systems	Actions	UVc
1	*Abies alba* Mill	Pinaceae	Silver fir	Brad argintiu	Native	Acute respiratory diseases	Expectorant	Buds	Internal	Syrup	1	1	0.055
						Bronchitis (12, 100)							
2	*Allium cepa* L	Amaryllidaceae	Onion	Ceapa	Exotic	Respiratory	Calming	Bulb, leaves	External	Baked, poultice	1	3	0.074
						Asthma (1, 4.1)	Relaxing						
						Bronchitis (23, 95.9)	Anti-inflammatory						
3	*Allium sativum* L	Amaryllidaceae	Garlic	Usturoi, ai	Exotic	Digestive	Anti-parasitic	Bulb, leaves	Internal	Raw bulb	1	1	0.012
						Intestinal worms (2, 50)			External–intra rectal				
						Intestinal parasites (2, 50)							
4	*Allium ursinum* L	Amaryllidaceae	Wild garlic, ramson	Leurda, usturoiul ursului	Native	General	Tonic	Leaves	Internal	Raw leaves	1	1	0.025
						Weakness (5, 100)	Immunostimulant						
5	*Anethum graveolens* L	Apiaceae	Dill	Marar	Native	Digestive	Relaxing Carminative	Aerial parts	Internal	Infusion	1	2	0.055
						Abdominal cramps (2, 13.3)		Seeds					
						Infantile colic (13, 86.7)							
6	*Arnica montana* L	Asteraceae	Mountain arnica	Arnica	Native	Musculoskeletal: Trauma (87, 100)	Vulnerary	Leaves	External	Ointment	1	2	0.264
							Anti-inflammatory						
7	*Calendula officinalis* L	Asteraceae	Common marigold	Galbenele	Native	Skin	Emollient	Capitulum	External	Ointment	1	3	0.365
						Diaper rash (92, 72.4)							
						Eczema (11, 8.7)	Vulnerary						
						Wounds (3, 2.4)	Anti-infective						
						Burns (3, 2.4)							
						Skin infections (18, 14.2)							
8	*Carum carvi* L	Apiaceae	Caraway	Chimen	Native	Digestive	Carminative	Seeds	Internal	Infusion	1	1	0.034
						Cramps (2, 13.3)							
						Infantile colic (13, 86.7)							
9	*Chelidonium majus* L	Papaveraceae	Greater celandine	Rostopasca, negelarita	Native	Skin	Anti-infective	Latex	External	Sap	1	2	0.095
						Warts (37, 100)	Immune stimulant						
10	*Cucurbita pepo* L	Cucurbitaceae	Pumpkin	Dovleac, bostan	Exotic	Digestive	Anti- parasitic	Seeds	Internal	Seeds	1	1	0.049
						Intestinal worms (18, 100)							
11	*Foeniculum vulgare* Mill	Apiaceae	Fennel	Fenicul	Exotic	Digestive	Carminative	Fruits	Internal	Infusion	1	1	0.055
						Intestinal cramps (2, 13.3)							
						Infantile colic (13, 86.7)							
12	*Helianthus annuus* L	Asteraceae	Common sunflower	Floarea soarelui	Exotic	Ear Ear pain (2, 100)	Analgesic	Seeds oil	External	Seed oil	1	1	0.006
13	*Hippophae rhamnoides* L	Elaeagnaceae	Sea buckthorn	Catina alba, catina de rau	Native	Respiratory Respiratory infections (74, 100)	Tonic Immune stimulant	Fruits	Internal	Syrup, infusion	1	2	0.224
14	*Hypericum perforatum* L	Hypericaceae	Saint John’s wort	Sunatoare, pojarnita	Native	Digestive	Vulnerary	Aerial parts	Internal	Infusion	1	1	0.043
						Liver diseases (4, 28.5)							
						Gallbladder dyskinesia (10, 71.5)							
15	*Matricaria spp.*	Asteraceae	Chamomile	Musetel, romanita	Native	1. Internal -Digestive	Relaxing	Capitulum	Internal External	Infusion Decoction	2	4	0.301
						Diarrhea (14, 11)							
						Infantile colic (86, 68)	Carminative						
						2.External–Skin and mucosa inflammation (19, 15)	Anti-inflammatory						
						Eczema (5, 4)	Anti-infective						
						Eye infection (3, 2)							
16	*Mentha spp.*	Lamiaceae	Mint	Menta, izma buna	Native	Digestive	Relaxing	All green parts	Internal	Infusion	1	4	0.509
						Diarrhea (154, 93.3)	Carminative						
						Bloating (11, 6.7)	Anti-infective						
							Anti-flatulent						
17	*Pimpinella anisum* L	Apiaceae	Anise	Anason	Exotic	Digestive	Relaxing Carminative	Fruits	Internal	Infusion	1	2	0.055
						Intestinal cramps (2, 13.3)							
						Infantile colic (13, 86.7)							
18	*Prunus avium (L.) L*	Rosaceae	Cherry	Cires	Native	Renal Urinary tract infections (2, 100)	Diuretic	Stalks	Internal	Infusion	1	1	0.006
19	*Raphanus raphanistrum subsp. sativus (L.) Domin*	Brassicaceae	Black radish	Ridiche neagra	Exotic	Respiratory Bronchitis (2, 100)	Anti-inflammatory	Root	Internal	Sap with honey	1	3	0.006
							Calming						
							Expectorant						
20	*Rosa canina* L	Rosaceae	Dog rose	Maces	Native	Respiratory Acute infective diseases (17, 100)	Tonic	Fruits	Internal	Infusion, syrup	1	3	0.052
							Anti-infective						
							Anti-inflammatory						
21	*Thymus serpyllum* L	Lamiaceae	Wild thyme	Cimbrisor de camp	Native	Respiratory Acute infective diseases (12, 100)	Anti-infective Expectorant	Aerial parts	Internal	Infusion	1	2	0.04
22	*Tilia tomentosa* Moench	Malvaceae	Silver linden	Tei argintiu, tei alb	Native	Psychological: Agitation (3, 5.5)	Relaxing Calming Sedative	Flowers	Internal	Infusion	2	3	0.153
						Anxiety (1, 2)							
						Respiratory							
						Acute infective diseases with cough (50, 92.5)							
23	*Triticum aestivum* L	Poaceae	Wheat	Grau	Exotic	Skin	Emollient	bran	External	Decoction	1	1	0.018
						Eczema (6, 100)							
24	*Urtica dioica* L	Urticaceae	Common nettle	Urzica	Native	General	Tonic	Young leaves	Internal	Infusion, young leaves cooked	1	1	0.101
						Weakness (13, 38.2)							
						Blood							
						Anemia (21, 61.8)							
25	*Viola tricolor* L	Violaceae	Heartsease	Trei frati patati	Native	General	Anti-allergic	Flowering branches	Internal	Infusion	1	1	0.012
						Allergies (4, 100)							

Legend- FL-fidelity level; UVc -use value citation index.

Apiaceae and Asteraceae were the dominant families with four species each followed by Amaryllidaceae (three species) and Lamiaceae and Rosaceae (two species). The highest proportion of the medicinal plants used is herbaceous (76%), followed by trees (12%), shrubs (8%), and grasses (4%). The remedies were made from fruits, leaves, roots, seeds, stems, buds, flowers, bulbs, or sap. In some cases, a combination of plants was administered.

Only 18% (95% CL: 15.87%–20.13%) of the probands have harvested themselves the plants cultivated in their gardens or growing in the surroundings, whereas the majority of 82% (95% CL: 79.87%–84.13%) have purchased processed plant parts (66%, 95% CL: 63.38%–68.62%) or already prepared remedies (16%, 95% CL: 13.97%–18.03%) from pharmacies, specialized stores, and local markets (Figure 3B).

Various methods of preparation were used for the 25 medicinal plant species: decoction, infusion, ointment, poultice, and syrup, with infusion as the most preferred method (56%), followed by decoctions and poultice (12%). Conversely, ointments, syrups, and the raw administration of plant parts, sap, or seeds were less frequently utilized, accounting for only 8% of the preparations. Some of the medicinal remedies were administered with the meal, either cooked (e.g., garlic bulbs) or raw (e.g., sea buckthorn fruits). Water was the main solvent employed for the herbal treatments.

The remedies were administered orally, as baths with cold or warm infusions, or as external applications (crushed or baked plant materials covering the affected body parts). In one case of intestinal worms (oxyurids), a garlic clove was inserted in the anus.

### 3.3 Pediatric diseases treated using medicinal plants

The diseases were grouped into seven categories, depending on the targeted body systems, among which digestive, skin, and respiratory tract diseases featured predominantly. Informant consensus factor (F_ic_) for each nosological category is provided in Table 3.

The most versatile species, having the largest area of indications were chamomile (*Matricaria spp*) and silver linden (*Tilia tomentosa* Moench.)*.*

Monotherapy preparations were predominant, although some diseases were treated with polyherbal mixtures. For instance, an infusion of a mixture containing fennel (*F. vulgare* Mill.), caraway (*Carum carvi* L.), dill (*Anethum graveolens* L.) seeds, and chamomile capitulum was used to treat infant colic, beginning at 1.5-month of age. Mint (*Mentha spp*.) leaves combined with chamomile capitulum were employed to treat diarrhea, while Saint John’s wort (*Hypericum perforatum* L.) flowers with mint leaves were used to treat gallbladder dyskinesia.

Silver fir (*Abies alba* Mill.), onion (*Allium cepa* L.), black radish (*Raphanus raphanistrum subsp. sativus* (L.) Domin), dog rose (*Rosa canina* L.), wild thyme (*Thymus serpyllum* L.) and silver linden were indicated for the treatment of respiratory diseases. Anise (*Pimpinella anisum* L.), chamomile*,* fennel, mint, caraway, Saint John’s wort, garlic (*Allium sativum* L.), pumpkin (*Cucurbita pepo* L.) and dill were administered to treat digestive ailments, whereas for skin conditions, chamomile*,* common marigold (*Calendula officinalis* L.), greater celandine (*Chelidonium majus* L.), and wheat (*Triticum aestivum* L.) were prescribed as plant remedies.

### 3.4 Medicinal plants ordered according to their ethnopediatric relevance

The highest number of mentions was registered for mint. It was used by 165 out of 326 herbal treatment employing women (50.61%, 95% CL: 47.84%–53.38%), with a mean administration time of 4.8 days. Interestingly, FL is 93.3 for diarrhea and only 6.7 for bloating. The youngest patient treated in this manner was a 3-month-old. Chamomile the next most frequently referenced plant, was reported 128 times (39.26%, 95% CL: 36.56%–41.97%), with the highest FL (68) recorded for infantile colic.

Common marigold was identified in 127 cases (38.96%, 95% CL: 36.26%–41.66%), being indicated in the treatment of various skin ailments, such as diaper dermatitis (FL 72.4), eczema (FL 8.7), etc. Prepared as an ointment, it was applied to the affected area since the first month of life. Another plant administered in ointments was the mountain arnica (*Arnica montana* L.) with 87 mentions (26.69%, 95% CL: 24.24%–29.14%). The indications were traumatic lesions (FL 100), such as concussions, sprains, and hematoma. The youngest patient that received this preparation was 2 years old.

Sea buckthorn (*Hippophae rhamnoides* L.) fruits, with 74 mentions (22.7%, 95% CL: 20.38%–25.02%), were indicated as a tonic in treatment of weakness and as an immunostimulant agent in acute respiratory diseases (FL 100). A three-year-old child was the youngest recipient. Also, ramson (*Alliumm ursinum* L.) was indicated as an immunostimulant, being eaten raw in salads (FL- 100). Silver linden flowers were referenced 54 times (16.56%, 95% CL: 14.51%–18.62%) and were utilized in the treatment of respiratory disorders, notably cough (with a FL- 92.5), as well as agitation and anxiety. The youngest patient treated was 3 years of age.

Greater celandine latex was mentioned in 37 cases (11.35%, 95% CL: 9.59%–13.11%), for the treatment of skin verruca (Fl- 100), with an anti-infective role. The patient at the lowest end of the age spectrum was 2 years old. Common nettle (*Urtica dioica* L.) young leaves were employed in 34 cases (10.43%, 95% CL: 8.74%–12.12%) to treat anemia (FL- 61.8) and weakness (FL-38.2), and a 1.5 years old was the youngest among the patients. Boiled wheat bran was applied as a poultice on dry dermatitis lesions (FL-100) with very good results.

Garlic bulbs and pumpkin seeds were indicated in intestinal parasites (FL-50 garlic) and worm treatment (FL-50 garlic, FL-100 pumpkin), whereas black radish sap with honey and dog rose was administered in acute respiratory diseases and cough (FL-100), in children aged over 3 years old.

### 3.5 Alignment of our data with other ethnopediatric sources or with the available bioscientific evidence

Our search in PubMed and Google Scholar for available data on the listed medicinal plants, comprising 25 in total, revealed the following results (for more details, please refer to the Table 2, Supplementary Material):

1) 22 plants were reported to have ethnopediatric practices in other countries. The plants lacking documented use in this regard are black radish, wheat, and wild thyme.
2) 21 plants were evaluated in clinical studies or mentioned in case reports involving children. The ones that have not undergone such investigations are black radish, silver fir, ramson, and silver linden.
3) 21 plants were assessed in clinical studies or mentioned in case reports in adults for similar purposes as those reported in our present research. Those that have not been subject to such scrutiny are fennel, greater celandine, pumpkin, and ramson.
4) 24 plants were reported to have *in vivo* various biological activities, supporting the ethnopediatric use documented herein. Silver fir was the only one without *in vivo* research backing.
5) 23 plants were found to exhibit various biological activities *in vitro*, consistent with their ethnopediatric use as reported in the present study. Those lacking *in vitro* studies include caraway and sea buckthorn.
6) 14 plants have been extensively studied, with scientific literature covering research for each category among the five mentioned above: anise, arnica, chamomile, cherry (*Prunus avium* (L.) L.), common marigold, common nettle, dill, garlic, heartsease (*Viola tricolor* L.), mint, onion, rosa canina, Saint John’s wort, and sunflower.

Based on this data, it appears that although the herbal species mentioned here for ethnopediatric practices are quite common, only part of them have been extensively researched regarding the illnesses reported by the respondents interviewed herein. Furthermore, the majority of the medical uses documented by the mothers enrolled in our survey align with the scientific literature.

### 3.6 Statistical considerations

The relationships between categorical variables like age groups, urban/rural residence, education levels, and numerical variables (e.g., mother’s age, count of used plants, count of treated ailments) were explored by conducting correlation and multivariate analyses, as outlined in the Data analysis section.

Shapiro-Wilk test demonstrated that none of the numerical variables followed a normal distribution. Therefore, non-parametric tests were used to assess the associations between numerical and categorical variables.

The associations between categorical parameters as evaluated by Fisher’s exact test are shown in Table 6.

**TABLE 6:** The correlation between categorical parameters as estimated by Fisher’s exact test.

		Residency		p-value	Education		p-value
		Rural	Urban		Inferior	Superior	
Used plants	≤2	123	68	0.56	62	129	**E-08**
	>2	82	53		9	126	
Ailments treated	≤2	129	73	0.64	61	141	**E-06**
	>2	76	48		10	114	
Administration route	External	51	29	0.89	18	62	0.88
	Internal	154	92		53	193	
Plant procurement	Purchased	154	109	**0.0008**	68	195	**8E-05**
	Harvested	51	12		3	60	
Preparation method	cooked	10	1	0.14	0	11	**0.0001**
	decoction	5	4		2	7	
	infusion	121	84		59	146	
	ointment	25	15		8	32	
	raw	23	11		2	32	
	syrup	21	6		0	27	
Recommendation source	family	95	46	**0.01**	26	115	**0.0001**
	friends	39	23		9	53	
	medical_prof	65	37		36	66	
	web_media	6	15		0	21	

Legend: As multiple (12) comparisons were performed, the significance level (commonly set at 0.05) was lowered according to Bonferroni correction: the corrected significance level was 0.05 divided by the number of comparisons, namely, α_corrected_ = 0.05/12 ≈ 0.004. The comparisons that yielded significant results were bold-typed.

The associations between numerical and binary categorical parameters as appraised by Mann-Whitney test are shown in Table 7, while those between numerical and multivalent categorical parameters as estimated by Kruskal-Wallis test, are displayed in Table 8.

**TABLE 7:** The correlation between numerical and dichotomous categorical parameters as estimated by Mann-Whitney (Wilcoxon) test.

Numerical parameters	Categorical parameters	#pts. Yes	#pts. No	Median yes [IQR]	Median no [IQR]	W Statistics	p-value
Treatment duration [days]	Internal administration	**246**	**80**	**5 [4–7]**	**7 [5–10]**	**14243.5**	**8E-10**
Ailments treated count	Internal administration	246	80	2 [1–3]	2 [1–3.25]	10372.5	0.5
Used plant count	Internal administration	246	80	2 [1–4]	2 [1–3]	9347.5	0.5
Mothers’ age [years]	Internal administration	246	80	37 [31–43]	37 [31–41]	9406	0.6
Child age at first herbal treatment	Internal administration	246	80	2 [0.5–4]	2.5 [0.33–4]	9843.5	1
Mothers’ age [years]	Ailments treated >2	124	202	38 [34–42.25]	36 [28–42]	10688	0.026
Treatment duration [days]	Ailments treated >2	124	202	5 [5–7.25]	5 [4–7]	11735	0.3
Child age at first herbal treatment	Ailments treated >2	124	202	2 [0.33–4]	2 [1–4]	13007	0.6
Used plant count	Higher educational level	**255**	**71**	**2 [1–4]**	**2 [1–2]**	**5461**	**1E-07**
Ailments treated count	Higher educational level	**255**	**71**	**2 [1–3.5]**	**2 [1–2]**	**6122.5**	**2E-05**
Mothers’ age [years]	Higher educational level	**255**	**71**	**38 [33–42]**	**32 [23–41]**	**6338.5**	**1E-04**
Child age at first herbal treatment	Higher educational level	**255**	**71**	**3 [0.5–5]**	**2 [0.79–2]**	**6544.5**	**3E-04**
Treatment duration [days]	Higher educational level	255	71	5 [5–7]	5 [4–7]	7993.5	0.1
Ailments treated count	Favorable outcome	274	52	2 [1–3]	3 [1–3.25]	8268	0.06
Used plant count	Favorable outcome	274	52	2 [1–3]	2.5 [1–4]	7884.5	0.2
Mothers’ age [years]	Favorable outcome	274	52	37 [31–42]	38 [31.25–43]	7595.5	0.4
Treatment duration [days]	Favorable outcome	274	52	5 [5–7]	5 [4–10]	6678.5	0.5
Child age at first herbal treatment	Favorable outcome	274	52	2 [0.5–4]	2 [0.31–5]	6803	0.6
Child age at first herbal treatment	Purchased	**263**	**63**	**2 [0.33–4]**	**3 [2–5]**	**11007**	**5E-05**
Ailments treated count	Purchased	**263**	**63**	**2 [1–3]**	**3 [2–4]**	**10266**	**0.002**
Used plant count	Purchased	**263**	**63**	**2 [1–3]**	**3 [2–4]**	**10113**	**0.005**
Mothers’ age [years]	Purchased	263	63	37 [29–42]	38 [33.5–42.5]	8956.5	0.3
Treatment duration [days]	Purchased	263	63	5 [4.5–7]	5 [5–10]	8836	0.4
Ailments treated count	Urban residency	121	205	2 [1–3]	2 [1–3]	11733.5	0.4
Used plant count	Urban residency	121	205	2 [1–4]	2 [1–3]	11737	0.4
Mothers’ age [years]	Urban residency	121	205	37 [31–42]	37 [31–43]	12734.5	0.7
Child age at first herbal treatment	Urban residency	121	205	2 [0.67–4]	2 [0.42–4]	12114	0.7
Treatment duration [days]	Urban residency	121	205	5 [4–7]	5 [5–7]	12220.5	0.8
Mothers’ age [years]	Used plant >2	135	191	38 [34–42]	36 [28–42.5]	10867.5	0.016
Treatment duration [days]	Used plant >2	135	191	5 [5–7]	5 [4–7]	12052.5	0.3
Child age at first herbal treatment	Used plant >2	135	191	3 [0.33–4]	2 [0.58–4]	12293.5	0.5

Legend: W statistics and *p*-values were calculated by means of Mann-Whitney test. As multiple (31) comparisons were performed, the significance level (commonly set at 0.05) was lowered according to Bonferroni correction: the corrected significance level was 0.05 divided by the number of comparisons, namely, α_corrected_ = 0.05/31 ≈ 0.0016. The comparisons that yielded significant results were bold-typed. #pts. = patients count; “yes” and “no” in the 3^rd^–5^th^ columns refer to categorical parameters in the second column. The median and the interquartile range [IQR] in the 5^th^ and 6^th^ columns are those of the numerical parameter in the 1^st^ column. For example, the numbers in the first row mean that 246 mothers declared having internally administered the herbal remedy, while 80 mothers used it externally; the median duration of internally given treatment was 5 days, while median duration of externally administered treatment was 7 days.

**TABLE 8:** The correlation between numerical and multivalent categorical parameters as estimated by Kruskal-Wallis test.

Parameter	Values	Mothers’ age [years] median (IQR)	Kruskal-Wallis chi-squared	p-value
Preparation method	cooked	36 (33–41)	6.2665	0.2811
	decoction	37 (36–40)		
	infusion	37 (28–42)		
	ointment	37 (30.5–41)		
	raw	39 (36–43.8		
	syrup	38 (31.5–40)		
Recommendation source	medical professionals (MP)	35 (26.2–40.8)	20.224	0.00015 (MP–WM: 0.001; MP–Fr: 0.0025; MP–Fa: 0.03; Fa–WM: 0.02)
	family (Fa)	37 (28–42)		
	friends (Fr)	38.5 (34.2–43)		
	web, media (WM)	42 (38–46)		

Legend: Kruskal-Wallis test was employed to calculate Kruskal-Wallis chi-squared statistics (in the 4^th^ column) and *p*-values (in the 5^th^ column). The parenthesis in the last cell of the table contains the *p*-values obtained by two-by-two comparisons using the pairwise Wilcoxon test, while Benjamini-Hochberg procedure was employed to decrease the false discovery rate. ∼ = compared with.

## 4 Discussions

More than half (59%) of the mothers interviewed reported using herbal remedies based on raw plant material (either self-harvested or purchased) to treat their children’s medical conditions. The primary sources of knowledge for these remedies were family and health professionals such as pharmacists and physicians. The significant proportion of informants (40.49%) who indicated that herbal remedies were the only treatment administered to their children could be attributed to several factors. Previous studies have suggested that parents may avoid pharmaceutical agents, including concentrated plant extracts, due to concerns about potential harm to their children, favoring unprocessed plant-based treatments instead (Ventola, 2010; Lucas et al., 2018). Additionally, mothers may consider herbal remedies as providing satisfactory results.

An anticipated outcome was that regarding the rural-urban disparity: the mothers of rural background have a higher propensity to use medicinal plants compared to mothers of urban background. This result may have various explanations. Adherence to urban culture may be responsible for a shift in the perceptions and attitudes toward traditional healthcare practices (Pimentel et al., 2021). Conversely, rural culture is characterized by slower economic development and higher poverty, leading to a preference for low-cost or homemade remedies over synthetic drugs.

The current study identified 25 medicinal plant species belonging to 15 families used in treating children’s diseases in Southern Romania. While direct comparisons to historical records, based on a larger overall knowledge of local healers, midwives, and doctors may not be ideal, in the absence of other local contemporary investigations on the same topic, we contrasted our findings with data reported in our previous study on plants historically relevant in Romanian ethnopediatrics. This previous study documented 153 medicinal plants from 52 families, excluding edible varieties like ramson (Petran et al., 2020).

Notably, there has been a significant decrease in the number of medicinal plants used for pediatric purposes, suggesting a reduction in ethnopediatric knowledge transmitted through generations in Romania. This decline, while serving as an indirect indicator, requires further validation. Comparing the therapeutic uses of the various plants reported in the present survey with those documented in our previous investigation, we found that only two plant species, *F. vulgare,* and *P. anisum,* were used for infantile colic in both studies. A clinical trial carried out in Italy confirmed the effectiveness of *F. vulgare* in treating infant colic when administered in combination with *Matricariae recutita* and *Melissa officinalis* (Savino et al., 2005). Some pediatric employments, traditionally acknowledged but previously unreported in Romania, were identified for four plant species: ear pain for *Helianthus annuus*, digestive diseases for *H. perforatum*, and respiratory disorders for *T. serpyllum* and *T. tomentosa*. However, our present study indicated fewer reported utilizations for most plant species compared to our previous research.

Asteraceae, Apiaceae, and Amaryllidaceae were the dominant families used in herbal remedy preparation. The utilization of a particular plant species to treat entirely different conditions could be attributed to the large range of active phytocompounds present in the plant, acting on various body systems. For instance, medicinal plants identified in our survey for treating a certain ailment were mentioned for dissimilar curative properties in other European countries: *A. sativum,* mentioned as an antiparasitic agent in our study, was found to be effective for whooping cough in Italy (Muhe et al., 1994); *C. carvi* used as carminative for children in Southern Romania, was employed for enuresis in Italy (Leporatti and Ghedira, 2009); *C. majus,* utilized for curing warts in our study, was reported in Poland for treating jaundice and digestive tract parasites (Zielińska et al., 2018); *H. perforatum,* used by Romanian mothers for treating children’s liver diseases, was used in Poland for burns treatment (Coppock and Dziwenka, 2021); *U. dioica,* popular for treating pediatric weakness and anemia in the geographic area investigated herein, was noted to be used as an antitussive in Spain (Rigat et al., 2015), whereas *V. tricolor*, utilized as an antialergic in our study, was used for treating seborrhoea of the scalp in nursing infants in Poland (Witkowska-Banaszczak et al., 2005), and as an expectorant in Ukraine (Herasymova et al., 2022).

Conversely, the use of the same plant species for treating a particular illness across various geographical regions may not only indicate their wide habitat range but also the widespread recognition of their efficacy in treating the respective illness. Similar ethnopediatric uses documented in our survey were also acknowledged in other European countries for the same medicinal plant: the treatment of respiratory diseases in Bosnia-Herzegovina with *Abies alba* (Prazina et al., 2011); the antiparasitic effects of *A. sativum* in Italy (Pieroni et al., 2005; Passalacqua et al., 2007; Dutto et al., 2012); *Allium ursinum* L*.* used as a tonic both in Italy and Poland (Leporatti and Ivancheva, 2003; Luczaj et al., 2012; Sõukand, 2016); recovery after local trauma with *Arnica montana* noted in Ireland (Crowe and Lyons, 2004); vulnerary effects of *Calendula officinalis* reported in Russia (Shaparenko et al., 1979); skin warts treated with *C. majus* in Italy (Leporatti and Corradi, 2001; Muršić, 2020); and *Matricaria chamomilla*, mentioned for treating digestive illnesses and colic in Germany (Nathan and Scholten, 1999).

The majority of the participants in our study have purchased plants and herbal remedies from specialized stores, local producers, and pharmacies. The remainder have harvested them from home gardens and available lands in the vicinity of their homesteads to avoid traveling long distances in their search for wild sources. Corresponding results have been reported in other surveys conducted in Eastern Europe (Pawera et al., 2017).

Most plant species were utilized primarily for managing respiratory, digestive, and dermatological conditions, likely stemming from the high prevalence of these diseases in children living in Southern Romania (“Institutul National De Sanatate Publica. Raportul Național de Sănătate a Copiilor Și Tinerilor Din Romania, 2011,” 2011). Infusion stood out as the predominant method for preparing herbal medicine, while water was the preferred solvent utilized. Alcoholic extracts are not commonly recommended in pediatric preparations due to their toxic potential (Gaw and Osterhoudt, 2019). The use as herbal baths in small children may have a rationale: transdermal delivery of pharmacological agents in this age group may be more efficient than their internal administration in adults (Delgado-Charro and Guy, 2014; Proksch, 2014; Visscher et al., 2015).

Regarding the minimal age of administration, we found that 7 out of the 25 medicinal plants were administered to babies younger than 3 months old: *Matricaria spp*, *Calendula officinalis*, *C. carvi*, *A. graveolens*, *F. vulgare*, *P. anisum*, *Mentha spp*. This information may be particularly relevant, considering the specific precautions imposed by the safety concerns associated with the employment of herbal treatment in newborns. Some plants (e.g., *Foeniculum vulgare*, *Matricaria spp*, *Calendula officinalis*) are acknowledged as safe for use in infants (Alexandrovich et al., 2003; Gozum et al., 2007; Martinelli et al., 2017; Sharifi-Heris et al., 2018) or as having minor negative outcomes such as skin irritations and/or reddening due to hypersensitivity reactions to the calendula extract in creams (Guala et al., 2007). However, more severe adverse events have been reported for certain plants, such as toxicity in two breastfed newborns resulting from excessive maternal use of *P. anisum* and other herbs, probably owing to the excretion of anethole in breastmilk (*Anise.*, 2006; Khoda Karami et al., 2008). There is a general scarcity of clinical studies in newborns and small children due to ethical concerns, but the available data on the use of herbs in nursing mothers may partially fill this gap (*Peppermint*, 2006).

### 4.1 Available scientific data on the most used medicinal plants for children’s diseases in Southern Romania

The majority of medicinal plants utilized by the mothers participating in our study to treat their children were employed for similar ailments as reported in ethnobotanical surveys, clinical studies, and case reports conducted worldwide in both children and adults, as revealed by our literature search in PubMed and Google Scholar (summarized in Table 2, Supplementary Material). This indicates their accurate utilization by our informants and the preservation of a solid knowledge base regarding the curative properties of the botanical remedies. Another issue highlighted by our search of the ethnomedical or bioscientific data on the identified medicinal plants was the scarcity of clinical studies on pediatric treatments with botanical remedies.

Mint and chamomile, the plants with the highest number of mentions in our survey were largely employed for ethnopediatric uses in other countries. The efficacy and safety of common marigold as a topical remedy for treating diaper dermatitis, an indication mentioned in the present study, was reported in a randomized comparative trial that included infants less than 3 years old. In topical applications, marigold yielded better outcomes than aloe (Panahi et al., 2012). Additionally, in another study conducted on 171 children aged 5–18 years with otalgia due to acute otitis media (an indication not mentioned in our study), Sarrell et al., 2003 found clinical benefits for naturopathic herbal extract ear drops containing marigold flowers, among other ingredients.

Mountain arnica has been utilized for treating various types of traumatic lesions. Scientific evidence supports its use in combination with echinacea to facilitate the detachment of the umbilical cord (Perrone et al., 2012) and for soft-tissue bruising in children aged 0–8.5 years (Thompson et al., 2010).

Ramson is most commonly consumed as food or spice in many countries (Abbet et al., 2014; Pawera et al., 2017; Mustafa et al., 2020). However, caution should be exercised, particularly in vulnerable groups such as young children, due to reported gastrointestinal toxicity in the literature (Fuchs et al., 2011; Lüde et al., 2016). Notably, a search with the phrase “*Allium ursinum* OR ramson OR wild garlic AND children” in PubMed and ScienceDirect databases provided no clinical studies. Silver linden’s flowers were employed to treat respiratory diseases, agitation, and anxiety. Some scientific evidence for anxiolytic activity is available (Gutiérrez et al., 2014), but it is not focused on pediatric use. Furthermore, another search using the phrase “*T. tomentosa* OR silver linden AND children” in PubMed and ScienceDirect databases revealed no clinical study.

Greater celandine fresh latex was reported here to be applied on skin verruca. Interestingly, in our previous historical review on Romanian medicinal plants with ethnopediatric use (1860s–1970s), another plant part and indication were identified: the root of greater celandine was administered as a bath for general strengthening (Petran et al., 2020).

Boiled wheat bran was found to be used mainly externally in dry dermatitis, whereas wheat in spray formulations was reported to be efficient for controlling gingival inflammation in schoolchildren (Bello et al., 2020). *In vitro* studies showed that wheat extract has modulated the expression of inflammation-associated molecules (Funel et al., 2020), and speeded up keratinocyte healing and tissue regeneration (Tito et al., 2020; Morretta et al., 2022). Clinical studies indicated wound healing effects as well, including the curing of venous leg ulcers (Romanelli et al., 2015) and the moisturizing effects of this plant extract (Boisnic et al., 2019).

Black radish, used for the treatment of respiratory disorders and cough in our study, was not yet mentioned in the scientific literature for pediatric use.

### 4.2 Dynamics of the ethnopediatric use of medicinal plants in Romania

Our research has identified a considerable decline in ethnobotanical knowledge compared to the earlier period examined by our team from 1860 to 1970, with only 25 medicinal plants identified, as opposed to the 153 previously documented (Petran et al., 2020). Several potential explanations for this trend may include the following:

• The information was collected from subjects residing in a relatively narrow geographical area i.e., Southern Romania.
• The respondents included in the study were domestic users of medicinal herbs, and not specialized healers possessing a sound knowledge of the matter.
• The accelerated economic development may have caused a gradual replacement of herbs by conventional drugs during the last decades. It is well-known that the globalization trend is associated with the abandonment of ancestral traditions by younger generations (Lee et al., 2001).
• The health benefits of medicinal plants as well as the results of the ethnopharmacological research are scarcely acknowledged and advertised.
• The health insurance systems provide no financial support for the use of herbals. By contrast, in other countries (e.g., Germany), such facilities are granted for the herbal treatment of children under a certain age or with certain ailments (e.g., developmental disorders), even without the need of a medical prescription (Du et al., 2014).
• The number of traditional healers (who might impart their knowledge to other members of the community) has tremendously decreased in modern times.
• Transmission of ethnobotanical knowledge declined due to the vicissitudes characteristic of the communist period (e.g., informational censorship, marginalization, and oppression of bourgeois experts and monastic communities).
• Deforestation in Romania has become a huge community problem (Petrişor and Petrişor, 2018; 2017; Peptenatu et al., 2020). According to the data available in the literature, deforestation may harm biodiversity and medicinal plant use (Shanley and Luz, 2003).

### 4.3 Statistical considerations

**FIGURE 4:**
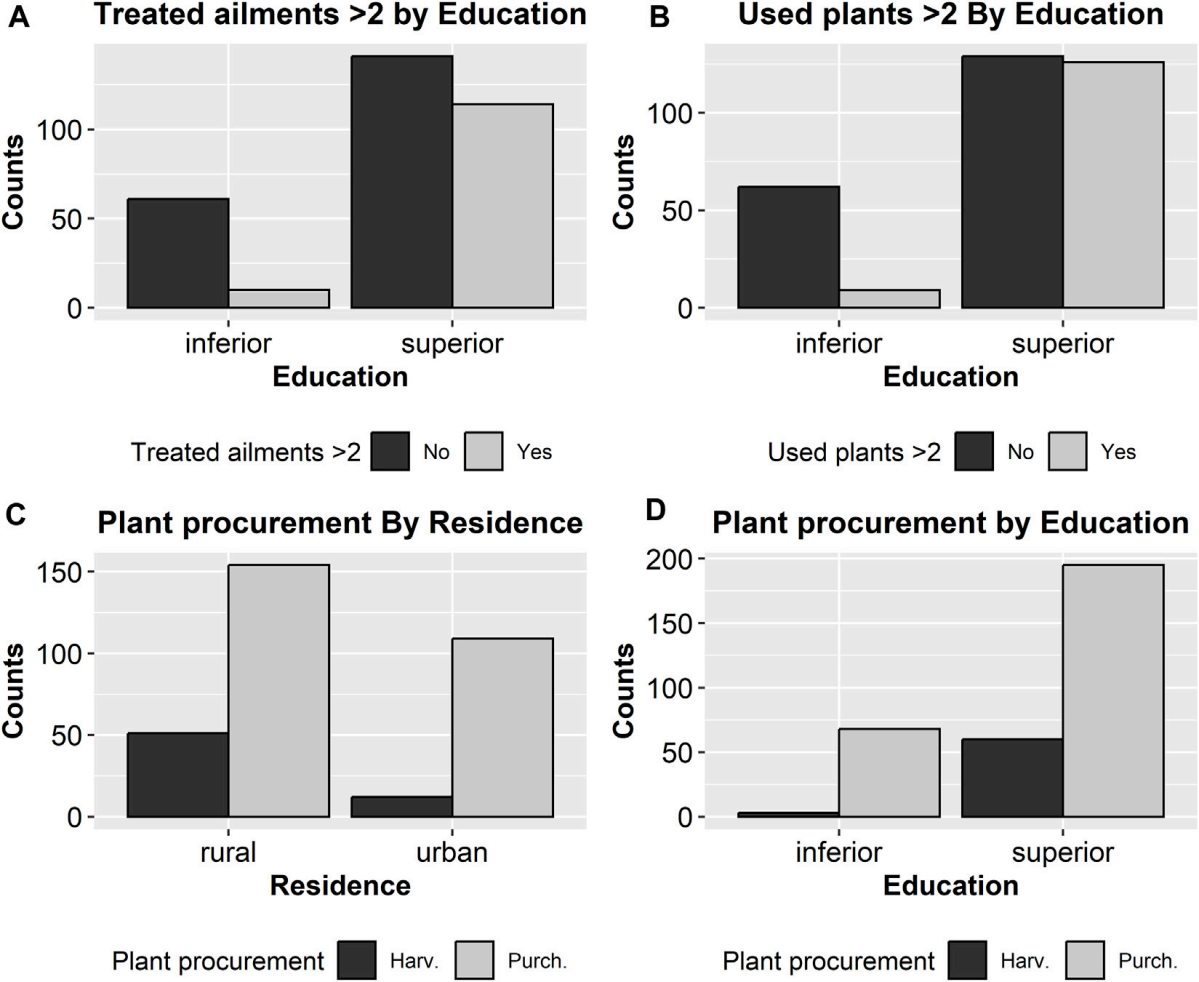
Significant correlations between binary categorical parameters as revealed by Fisher’s exact test. The corresponding *p*-values are 10^−6^ **(A)**, 10^−8^ **(B)**, 0.0008 **(C)**, 8 × 10^−5^ **(D)**.

Statistical analysis revealed intriguing and valuable findings regarding the relationships between categorical and numerical variables documented in this study. Fisher’s exact test (Table 6) demonstrated that only the education level (among the categorical parameters) was significantly associated with both the number of employed plants and the number of treated ailments (the higher the educational level, the greater the number of employed plants and the number of treated ailments) (Figure 4).

**FIGURE 5:**
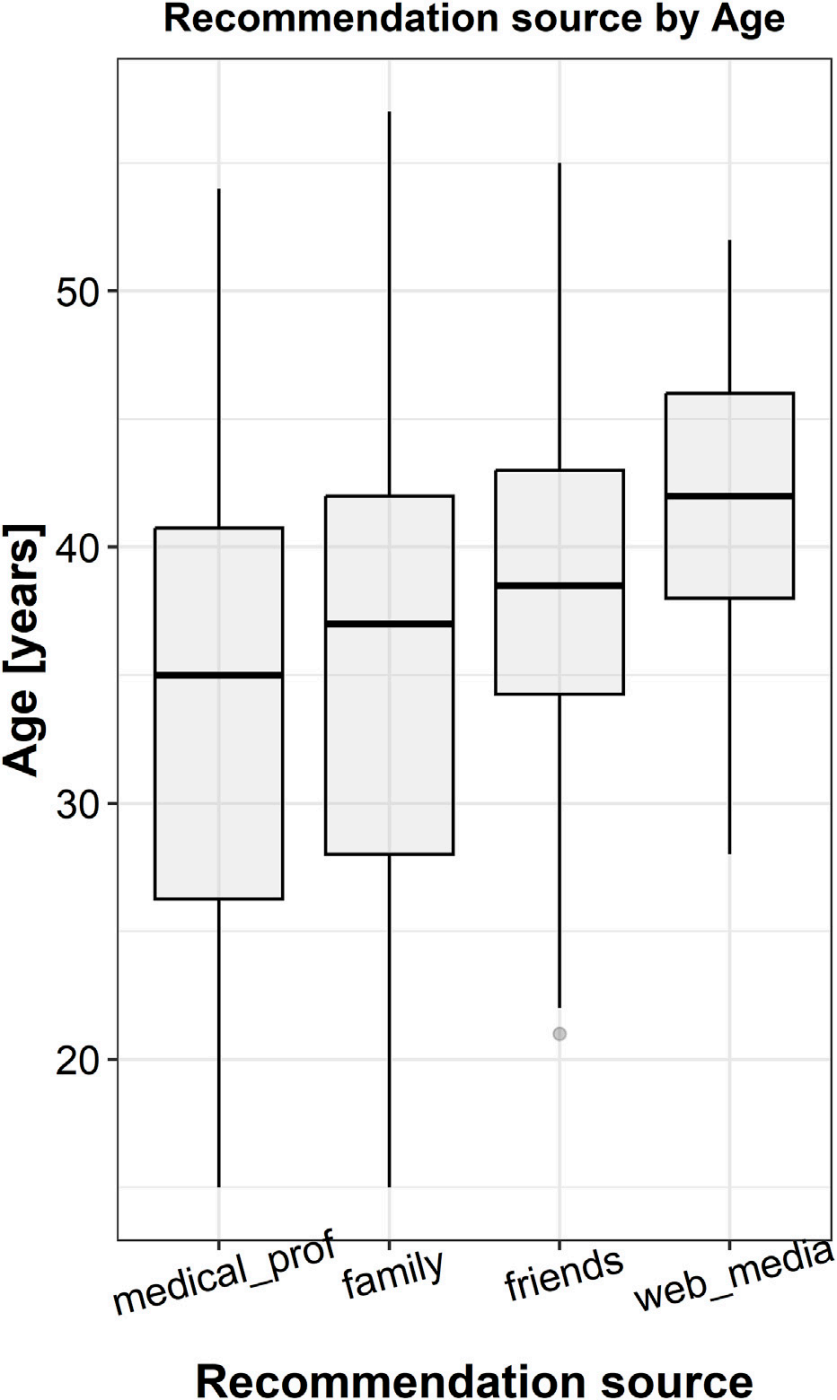
Significant correlation between the source of information and mothers’ age as revealed by Kruskal-Wallis test. The corresponding *p*-value is 0.00015.

The manner herbs were procured is also correlated with education level—somehow counterintuitively, mothers with higher educational levels had a greater propensity to harvest the plants themselves. A rural background is also associated with a higher probability of using plants harvested (rather than purchased) by the mothers (Figure 5). Remarkably, education level was not associated with residency status.

**FIGURE 6:**
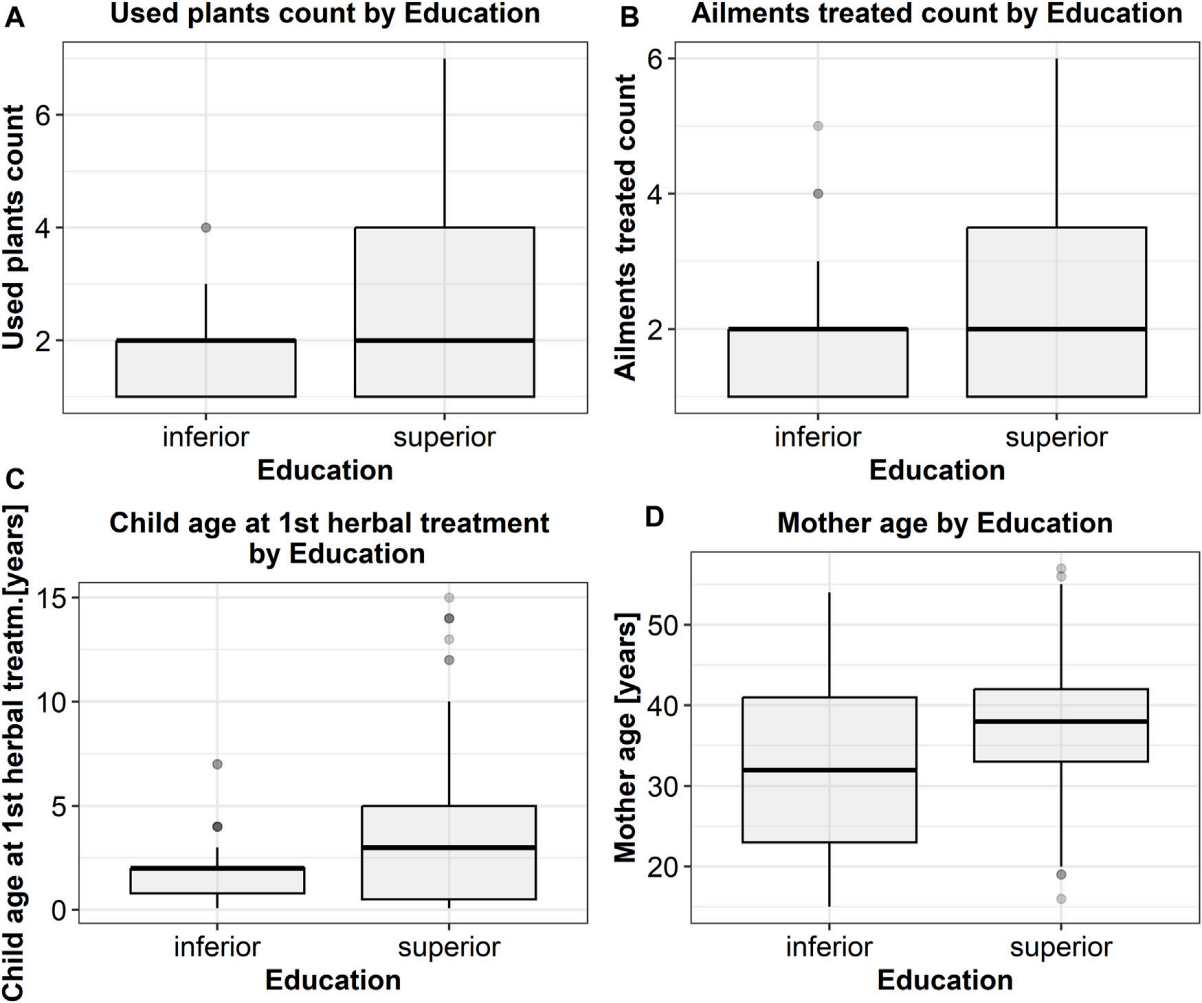
Significant correlation between numerical and categorical parameters as revealed by Mann-Whitney test. The corresponding *p*-values are 10^−7^ **(A)**, 2 × 10^−5^ **(B)**, 3 × 10^−4^ **(C)**, 10^−4^ **(D)**.

Fisher’s exact test (Table 6) demonstrated that the preparation method was also influenced by education level: while infusion was by far the most prevalent preparation method in both subgroups (lower and higher educated), higher-educated mothers utilized a more diverse range of preparation methods, in contradistinction to lower educated mothers, which used almost exclusively infusion as a preparation method (Figure 6).

**FIGURE 7:**
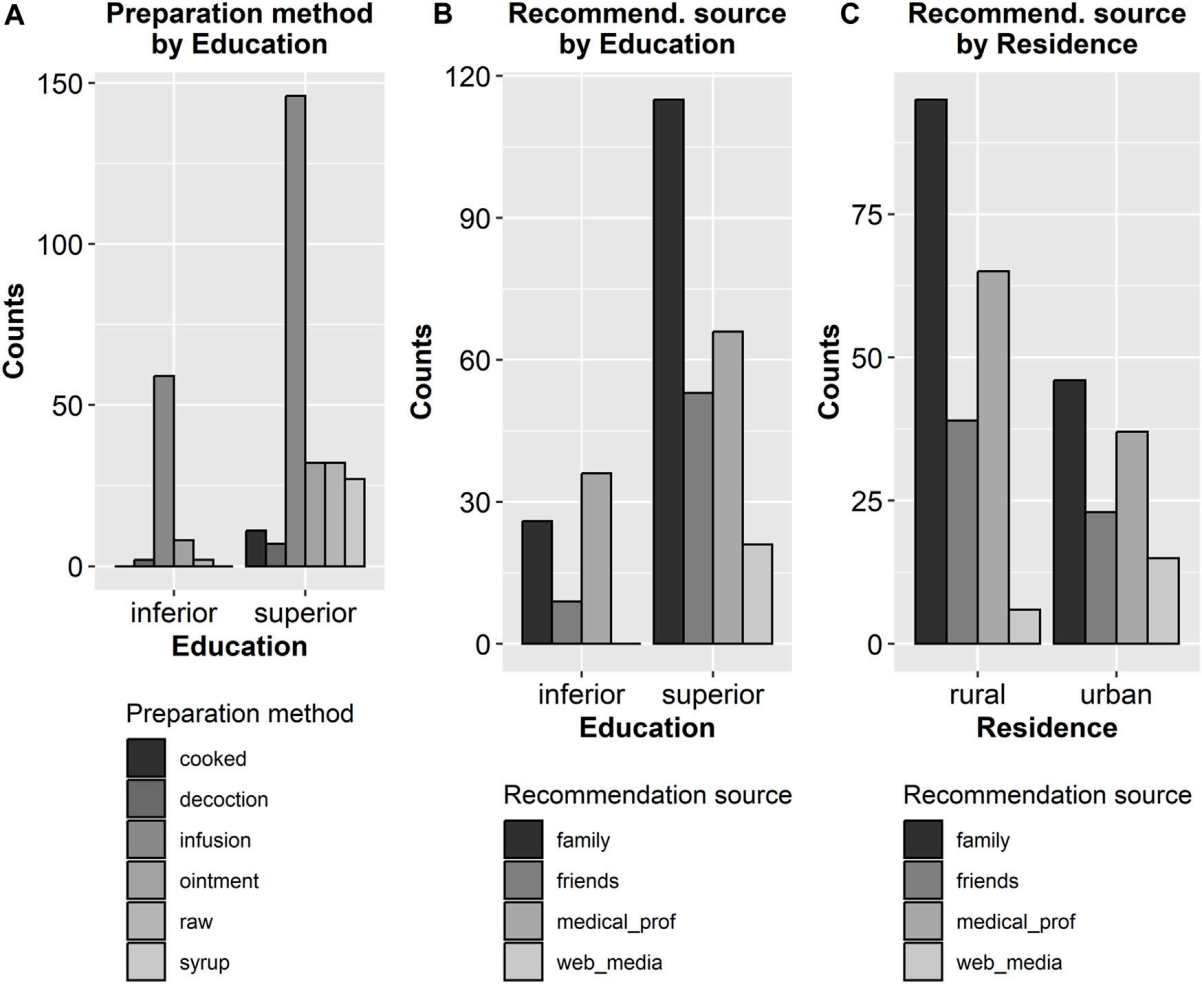
Significant correlations between binary and multivalent categorical parameters as revealed by Fisher’s exact test. The corresponding *p*-values are 0.0001 **(A)**, 0.0001 **(B)**, 0.01 **(C)**.

Education level also influenced the recommendation source employed by the mothers (Table 7): for lower educated mothers, medical professionals, and family members (in this order) were the most important sources, while for higher educated mothers, family members (not medical professionals) were by far the most important source, while web and media were also a significant source (absent in lower educated mothers) (Figure 7).

A similar result was found for the association between residency and the source of information: for mothers originating from a rural environment, family members and medical professionals (in this order) being by far the most important sources, while for the mothers dwelling in urban areas the information sources were more uniformly represented, with the web and media having greater importance (Figure 4).

Kruskal-Wallis test too revealed an interesting and rather unexpected result (Table 8): mothers using the web and media as an information source were generally older, while those seeking the advice of medical professionals tended to be younger (Figure 4)

Mann-Whitney test (Table 7) confirmed that a higher educational level was associated with a greater number of both employed herbs and treated diseases, as well as with a later start in treating the children with herbs. In other words, lower-educated mothers had a tendency to initiate herbal treatment for their children at an earlier stage. Harvesting (as opposed to purchasing) medicinal plants was also associated with a higher count of employed herbs and treated disorders, and a later start in the employment of herbal treatment in children. This association can be attributed, at least partially, to the correlation between a higher education level and a more pronounced inclination to harvest (rather than purchase) the plants.

In the MVA, age, residency, education level, and procurement method were all entered as putative independent parameters in the determinism of both the count of employed plants and the count of treated disorders (dependent parameters). MVA revealed that education level was the only independent determinant for both of these parameters. Interestingly, both residency and education level emerged as independent determinants of procurement method, while age did not show significant independent influence.

### 4.4 Study limitations

The number of informants included in the study was relatively reduced, mainly due to the specific restrictive conditions during the SARS-COV19 pandemic. Furthermore, the majority of informants resided in urban areas, whereas traditional knowledge regarding the therapeutic use of herbal remedies is typically better preserved in rural zones. Consequently, the conclusions drawn from our survey may not apply to the entire population of Romania, as our informants were exclusively from Southern Romania, and other regions may exhibit variations in medicinal flora and/or folk medical traditions.

Additionally, no herbal authentication was conducted, with the results solely relying on self-reports from our informants. Although botanical authentication would have been ideal, it was deemed impractical for commercial plant products subjected to processing, such as fragmentation or powdering, which eliminates morphological characteristics necessary for accurate macroscopical analyses (Pawar et al., 2017; Ichim et al., 2020).

Furthermore, while all vernacular names of medicinal plants provided by informants were common, there were two instances where these denominations failed to differentiate between several similar taxa in the genus Mentha and Matricaria. As a result, a definitive botanical identification of these two particular taxa cannot be asserted.

## 5 Conclusion

Medicinal plants continue to hold a relatively important role in treating various children’s diseases in Southern Romania, although the number of taxa used nowadays seems to have decreased compared to the past. This survey highlighted that the level of education was associated with the number of employed plants and the range of ailments treated, while residency (rural *versus* urban) was not. Interestingly, both residency and education influenced the method plants were procured: the inclination to harvest (rather than purchase) the plants was associated, as expected, with a rural background and, surprisingly, with a higher educational level.

Whether this traditional knowledge is fading away or dynamically adapting to economic, political, and cultural changes amid the pressures of world globalization is yet to be established (Heinrich, 2010; Pardo-de-Santayana et al., 2022). Ethnopediatric practices in Romania constitute a valuable heritage that urgently requires protection against potential cultural erosion and warrants further exploration to unlock their maximum benefits.

## Data Availability

The raw data supporting the conclusion of this article will be made available by the authors, without undue reservation.
