# Supplementary material for: Current use of medicinal plants for children’s diseases among mothers in Southern Romania
Source: Front Pharmacol. 2024 May 22;15:1377341. doi: 10.3389/fphar.2024.1377341 (PMC11150775; doi:10.3389/fphar.2024.1377341)
Supplement: Supplementary file 1 [file Table1.DOCX]

**QUESTIONAIRE no.:**

**Data Regarding the MOTHER:**

**Age: Education level: Childhood residence and landform:**

**Present residence and landform: Religion: Employment status: employed/ unemployed**

**Number of children:**

**Plant treatment in childhood:**

Did the mother receive plant treatment? (Yes/No)

If yes, specify the plant and the treated condition:

**Present plant treatment:**

Does the mother currently use plant treatment? (Yes/No)

If no, please provide the reason why not:

**Data Regarding the CHILD**:

**Age: Gender:**

**Plant-based treatments:**

Does the child receive plant-based treatments? (Yes/No)

If no, please provide the reason:

Age at first plant-based treatment:

Plant origin:

Picked and/or prepared by the family

Purchased

Preference for plant source: Family prepared/ Purchased

**Plant Treatment Data Required:**

- **Plant name** (Latin name where possible):
- **Source of information**: family members, physicians, pharmacists, friends, media (web, press, commercials, parenting books), others
- **Recommender:** family members, physicians, pharmacists, friends, media (web, press, commercials, parenting books), others
- **Medical conditions recommended for treatment:**
- **Effect on disease/medical condition evolution:** no effect; improvement; fully cured
- **Administration route:** external route (bath, topically, rubs, fumigations, others: ); internal route
- **Preparation types:** infusions, decoctions, tinctures, syrups, ointments, raw preparations, poultices, others
- **Administration as sole treatment or in association with other treatments/medication:**
- **Frequency/ dosage/ treatment duration:** how many times/day, quantity per administration (dosage), number of days
- **Adverse reactions:** if present, specify observed reactions (nausea, vomiting, allergies, rashes, others)
- **First time/ age of the child when plant treatment initiated:**
- **Frequency of remedy use:**
- **Other observations:**

| **Plant name**  **(Latin name where possible)** | **Plant parts used** | **Source of information** | **Medical conditions**  **recommended for treatment** | **Effect on medical condition evolution** | **Route of administration** | **Type of preparation** | **Administered alone/ with other types of treatment** | **Frequency/dosage/ treatment duration** | **Adverse reactions** | **First time/age when treatment was initiated** | **Frequency of treatment use** | **Other observations** |
| --- | --- | --- | --- | --- | --- | --- | --- | --- | --- | --- | --- | --- |
|  |  |  |  |  |  |  |  |  |  |  |  |  |
|  |  |  |  |  |  |  |  |  |  |  |  |  |
|  |  |  |  |  |  |  |  |  |  |  |  |  |
|  |  |  |  |  |  |  |  |  |  |  |  |  |
|  |  |  |  |  |  |  |  |  |  |  |  |  |
|  |  |  |  |  |  |  |  |  |  |  |  |  |
|  |  |  |  |  |  |  |  |  |  |  |  |  |
|  |  |  |  |  |  |  |  |  |  |  |  |  |
|  |  |  |  |  |  |  |  |  |  |  |  |  |
|  |  |  |  |  |  |  |  |  |  |  |  |  |
