# Supplementary material for: Current use of medicinal plants for children’s diseases among mothers in Southern Romania
Source: Front Pharmacol. 2024 May 22;15:1377341. doi: 10.3389/fphar.2024.1377341 (PMC11150775; doi:10.3389/fphar.2024.1377341)
Supplement: Supplementary file 2 [file Table2.pdf]

Table S2. The ethnopediatric studies and bioscientific evidence for the medicinal plants identified in the present study

**Nr.**	**Plant (Latin name, Family, English name)**	**Indications and biological activities reported in the present study**	**Ethnopediatric use in other countries previously reported in the literature**	**Clinical studies or case reports in children**	**Clinical studies or case reports in adults** (for similar use as the one reported in the present study)	***In vivo* studies** (supporting the use reported in the present study)	***In vitro* studies** (supporting the use reported in the present study)
1.	*Abies alba* Mill., Pinaceae (silver fir)	Acute respiratory diseases: bronchitis (expectorant)	*Bosnia-Herzegovina*: respiratory diseases (Prazina et al., 2011)	-	Respiratory: benefits as an aerosol of a water-alcohol extract for respiratory tract diseases (Baraneţchi et al., 1985)	-	Antioxidant, cytotoxic, antibacterial (Macovei et al., 2023), antifungal (Valková et al., 2022)
2.	*Allium cepa* L., Amaryllidaceae (onion)	Respiratory: asthma, bronchitis (calming, relaxing, anti-inflammatory)	*India:* jaundice; growth and development - onions with jaggery; colic - onion juice with salt (Singh and Khar, 2022) *Indonesia*: fever – compresses as febrifuge (Erda et al., 2021)	Skin: alopecia areata – topical crude onion juice (Sharquie and Al-Obaidi, 2002)	Respiratory: inhibition of allergen-induced bronchial obstruction (Dorsch et al., 1987), viral flu with mild symptoms (Tan et al., 2020)	Respiratory: reducing lung inflammation, oxidative stress and tracheal responsiveness in a murine model of asthma (Oliveira et al., 2015; Ghorani et al., 2018; Marefati et al., 2018; Shakeri et al., 2023), antibacterial	Relaxant on the tracheal smooth muscle (Memarzia et al., 2019), anti-inflammatory (Takahashi and Shibamoto, 2008; El-Hashim et al., 2020), fungicidal (Kocić-Tanackov et al., 2017), antibacterial (Ziarlarimi et al., 2011)

Nr.	Plant (Latin name, Family, English name)	Indications and biological activities reported in the present study	Ethnopediatric use in other countries previously reported in the literature	Clinical studies or case reports in children	Clinical studies or case reports in adults (for similar use as the one reported in the present study)	*In vivo* studies (supporting the use reported in the present study)	*In vitro* studies (supporting the use reported in the present study)
						(Akrami et al., 2015)	
3.	*Allium sativum* L., Amaryllidaceae (garlic)	Digestive: intestinal worms, intestinal parasites (anti-parasitic)	*Benin, Nigeria:* convulsions (Ayivi and Dan, 1990) *Ethiopia:* teething symptoms (Getaneh et al., 2018) *Italy:* intestinal worms (Passalacqua et al., 2007; Dutto et al., 2012); antimalarial (Gurmu et al., 2018); whooping cough (Muhe et al., 1994); anthelmintic (Pieroni et al., 2005;	Digestive: antiparasitic in infections with Hymenolepiasis nana or Giardia lamblia (Soffar and Mokhtar, 1991), treatment of *Blastocystis* and giardiasis in children (Abdo et al., 2023), oral antibacterial as mouth rinse (Thomas et al., 2017), biomaterial for pulpotomy of primary molars (Faghihi et al., 2021) Skin: antiviral in verruca vulgaris	Digestive: anti Helicobacter pylori (Zardast et al., 2016) Antifungal: anti Cryptococcus neoformans (Davis et al., 1990)	Digestive: antiparasitic in various animal models (Abd El-Galil and Aboelhadid, 2012; Cortes et al., 2017; Abd-ELrahman et al., 2022)	Antiparasitic (Fallahi et al., 2016; Băieş et al., 2022), vermicidal (Krishnakumari and Majumder, 1960), anthelmintic (Iqbal et al., 2001; Urban et al., 2008a)

Nr.	Plant (Latin name, Family, English name)	Indications and biological activities reported in the present study	Ethnopediatric use in other countries previously reported in the literature	Clinical studies or case reports in children	Clinical studies or case reports in adults (for similar use as the one reported in the present study)	*In vivo* studies (supporting the use reported in the present study)	*In vitro* studies (supporting the use reported in the present study)
			Passalacqua et al., 2007)	(Silverberg, 2002)<br>Respiratory: non-specific prevention of acute respiratory infections (Andrianova et al., 2003), increased arterial oxygen pressure in hepatopulmonary syndrome (Sani et al., 2006)<br>Ear: analgesic in otalagia associated with acute otitis media (Sarrell et al., 2001)			
4.	*Allium ursinum* L.,	General: weakness (tonic,	*India*: tonic and immune booster (Shahrajabian,	-	-	General: antioxidant properties in	Tonic (Leahu et al., 2015), antioxidant (Nikkhahi et al.,

Nr.	**Plant (Latin name, Family, English name)**	**Indications and biological activities reported in the present study**	**Ethnopediatric use in other countries previously reported in the literature**	**Clinical studies or case reports in children**	**Clinical studies or case reports in adults** (for similar use as the one reported in the present study)	***In vivo* studies** (supporting the use reported in the present study)	***In vitro* studies** (supporting the use reported in the present study)
	Amaryllidaceae<br>(wild garlic, ramson)	immune stimulant)	2021); tonic (Khan et al., 2017)<br>*Italy, Poland*: edible tonic (Leporatti and Ivancheva, 2003; Luczaj et al., 2012; Sõukand, 2016)<br>*Serbia*: edible spice (Jarić et al., 2007a)			hypercholesterolemic rabbits (Bombicz et al., 2016)	2018; Pop et al., 2020; Davidović-Plavšić et al., 2021; Kovačević et al., 2023), antimicrobial (Barbu et al., 2023), antimicrobial and antiparasitic (Krstin et al., 2018), antibacterial and antifungal (Galdiero et al., 2020), inhibitory effect against HIV-1- and HIV-2-induced cytopathicity (Smeets et al., 1997), antioxidant and antiproliferative (Sobolewska et al., 2006; Stanisavljević et al., 2020), antimicrobial, antioxidant and

Nr.	Plant (Latin name, Family, English name)	Indications and biological activities reported in the present study	Ethnopediatric use in other countries previously reported in the literature	Clinical studies or case reports in children	Clinical studies or case reports in adults (for similar use as the one reported in the present study)	*In vivo* studies (supporting the use reported in the present study)	*In vitro* studies (supporting the use reported in the present study)
							spasmolytic (Pavlović et al., 2017), antioxidant (BÂRLĂ et al., 2016)
5	*Anethum graveolens* L., Apiaceae (dill)	Digestive: abdominal cramps, infantile colic (relaxing, carminative)	*India*: infantile colic, digestive illnesses (Nair and CHANDA, 2007; Dhiraj and Anjna, 2013), carminative, antiflatulent (Shekhawat and Jana, 2010) *Iran*: infantile colic, digestive ailments (Sherkatolabbasieh et al., 2021), gastrointestinal disorders (Bahmani et al., 2014)	Digestive: antigiardial (Sahib et al., 2014)	Digestive: relaxing (Heidarifar et al., 2014), carminative (Hekmatzadeh et al., 2014), antispastic in irritable bowel disease (Mohammad, 2012), protects esophageal mucosa (Nam et al., 2021)	General: anti-inflammatory (Naseri et al., 2012),	Antiparasitic (Abas and Elagib, 2021), antimicrobial (Kaur and Arora, 2008; Castro et al., 2017)

Nr.	Plant (Latin name, Family, English name)	Indications and biological activities reported in the present study	Ethnopediatric use in other countries previously reported in the literature	Clinical studies or case reports in children	Clinical studies or case reports in adults (for similar use as the one reported in the present study)	*In vivo* studies (supporting the use reported in the present study)	*In vitro* studies (supporting the use reported in the present study)
6.	*Arnica montana* L., Asteraceae (mountain arnica)	Musculoskeletal: trauma (vulnerary, anti-inflammatory)	*Ireland*: control bruising, reduce swelling, and promote recovery after local trauma (Crowe and Lyons, 2004)<br>*Mexico*: wound healing (Alonso-Castro et al., 2022)	Skin: detachment of the umbilical cord (Perrone et al., 2012), soft tissue bruising vulnerary (Thompson et al., 2010a)<br>Musculoskeletal: external and internal bruising (Kaplan, 1994; Thompson et al., 2010b), antalgic and vulnerary (Crowe and Lyons, 2004)<br>Eye: dry eye treatment (Buzzonetti et al., 2023)	Musculoskeletal: effective treatment of swelling and bruising of the face (Lee et al., 2007a), anti-inflammatory, used in post-sport ankle sprain (Jurcău and Jurcău, 2013), accelerates postoperative healing, with a quicker resolution of the extent and the intensity of ecchymosis after osteotomies in rhinoplasty surgery (Chaiet and Marcus, 2016), muscle pain reliever (Adkison et al., 2010), effective in post-surgical setting, pain and	Musculoskeletal: antiinflammatory (Macêdo et al., 2004; Röhrl et al., 2023), vulnerary (Jia et al., 2006)	Musculoskeletal: promoter of healing (Olioso et al., 2016), anti-inflammatory (Röhrl et al., 2022)

Nr.	Plant (Latin name, Family, English name)	Indications and biological activities reported in the present study	Ethnopediatric use in other countries previously reported in the literature	Clinical studies or case reports in children	Clinical studies or case reports in adults (for similar use as the one reported in the present study)	*In vivo* studies (supporting the use reported in the present study)	*In vitro* studies (supporting the use reported in the present study)
					inflammation (Iannitti et al., 2016)		
7.	*Calendula officinalis* L., Asteraceae (common marigold)	Skin: diaper rash, eczema, wounds, burns, skin infections (emollient, vulnerary, anti-infective)	*Russia*: vulnerary suppurative otitis media (Shaparenko et al., 1979)	Skin: diaper dermatitis (Panahi et al., 2012), diaper dermatitis (Adib-Hajbaghery et al., 2014)<br>Ear: analgesic in ear pain associated with acute otitis media (Sarrell et al., 2001, 2003)	Skin: positive effects of the ointment with marigold extract on venous ulcer epithelialization (Duran et al., 2005), vulnerary in diabetic foot ulcers (Buzzi et al., 2016)	Skin: radiodermatitis healing (Schneider et al., 2015)	Vulnerary (Fronza et al., 2009; Nicolaus et al., 2017), anti-infective (Chakraborthy, 2008)
8.	*Carum carvi* L., Apiaceae (caraway)	Digestive: cramps, infantile colic (carminative)	*Italy, Tunisia*: enuresis (Leporatti and Ghedira, 2009)<br>*Morocco*: treat infantile colic (Said et al., 2015)	Digestive: appetite stimulant (Prangya Paramita Pati et al., 2023)	Digestive: improves bowel movements (Yousefi et al., 2019), anti-dyspepsia (Thompson Coon and Ernst, 2002)	Digestive: relaxes intestinal muscles (Al-Essa et al., 2010)	
9.	*Chelidonium majus* L.,	Skin: verruca	*Italy*: wart treatment	Skin: benefits in skin wart -	-	Skin: wart treatment	HPV replication inhibition (Nawrot et

Nr.	Plant (Latin name, Family, English name)	Indications and biological activities reported in the present study	Ethnopediatric use in other countries previously reported in the literature	Clinical studies or case reports in children	Clinical studies or case reports in adults (for similar use as the one reported in the present study)	*In vivo* studies (supporting the use reported in the present study)	*In vitro* studies (supporting the use reported in the present study)
	Papaveraceae (greater celandine)	(anti-infective, immune stimulant)	(Muršić, 2020), (Leporatti and Corradi, 2001) *Iran*: wart treatment (Miraldi et al., 2001) *Poland*: jaundice and digestive tract parasites (Zielińska et al., 2018)	antiviral (Nawrot et al., 2020) General: improved overall humoral and cellular immunity response (Khmel'nitskaia et al., 1998)		(Sterling et al., 2014) General: anti-retroviral activity in mice (Gerenčer et al., 2006), anti-inflammatory (Lee et al., 2007b), anti-hepatic tumor in mice (Biswas et al., 2008)	al., 2021; Musidlak et al., 2022)
10.	*Cucurbita pepo* L., Cucurbitaceae (pumpkin)	Digestive: intestinal worms (anti-parasitic)	*Iran:* anthelmintic (Moosavy et al., 2018) *Turkey*: anthelmintic (Kozan et al., 2006); endocrine diseases (SARI et al., 2023a)	General: food supplement trial (Buzigi et al., 2020) Blood: anemia treatment (Resmi et al., 2017)	-	Digestive: pronounced anthelmintic effect (A. ACORDA et al., 2019; Băieş et al., 2023), *in vitro* and *in vivo* anthelmintic efficiency (Grzybek et al., 2016),	Anthelmintic efficiency (Urban et al., 2008b; Okpo and Nnajekwu, 2013; Grzybek et al., 2016), antiparasitic (Maldonade et al., 2020; Băieş et al., 2022), anthelmintic efficacy(Hussein and Shukur, 2020; Boros

Nr.	Plant (Latin name, Family, English name)	Indications and biological activities reported in the present study	Ethnopediatric use in other countries previously reported in the literature	Clinical studies or case reports in children	Clinical studies or case reports in adults (for similar use as the one reported in the present study)	*In vivo* studies (supporting the use reported in the present study)	*In vitro* studies (supporting the use reported in the present study)
						antiparasitic in a mouse model (Beshay et al., 2019), anthelmintic in ostrich (Feitosa et al., 2012), anthelmintic in mice (Erol and Hamit, 2015; Alhawiti et al., 2019; Ashour et al., 2023; Saleh et al., 2024), anthelmintic in sheep (Meenakshisundaram et al., 2017)	et al., 2021; Salman and Ardalan, 2022)
11.	*Foeniculum vulgare* Mill., Apiaceae (fennel)	Digestive: intestinal cramps, infantile colic (carminative)	*India:* treatment of colic in children (Badgujar et al., 2014) *Morocco*: treatment of	Digestive: benefits in infantile colic (Savino et al., 2005)	-	Digestive: decreasing colic intensity (Alexandrovich et al., 2003)	-

Nr.	Plant (Latin name, Family, English name)	Indications and biological activities reported in the present study	Ethnopediatric use in other countries previously reported in the literature	Clinical studies or case reports in children	Clinical studies or case reports in adults (for similar use as the one reported in the present study)	*In vivo* studies (supporting the use reported in the present study)	*In vitro* studies (supporting the use reported in the present study)
			infantile colic (Said et al., 2015)				
12.	*Helianthus annuus* L., Asteraceae (common sunflower)	Ear: ear pain (analgesic)	*India* : skincare of infants in Ayurveda (KHATRI, 2013)<br>*Turkey*: atopic dermatitis (Akbaş et al., 2022)	Skin: topically applied sunflower seed oil improved skin conditions, prevented invasive bacterial infections in preterm infants (Darmstadt et al., 2004), skin microbiome alteration (Fischer et al., 2021), skin barrier recovery (Ahmed et al., 2007; Summers et al., 2019)<br>General: sunflower oil	Skin: preservation of skin barrier- potentially useful in ear pain (Danby et al., 2013)	Skin: wound healing properties- potentially useful in ear pain (Abhishek Anand et al., 2012)	Antimicrobial activity - potentially useful in ear pain (Selected Evidence-Based Health Benefits of Topically Applied Sunflower Oil, 2015)

Nr.	Plant (Latin name, Family, English name)	Indications and biological activities reported in the present study	Ethnopediatric use in other countries previously reported in the literature	Clinical studies or case reports in children	**Clinical studies or case reports in adults** (for similar use as the one reported in the present study)	***In vivo* studies** (supporting the use reported in the present study)	***In vitro* studies** (supporting the use reported in the present study)
				supplementation as an alternative to corn-soy blends, treatment of moderate acute malnutrition among children aged 6 to 59 months (Nane et al., 2021), children with familial hypercholesterolemia (Negele et al., 2015), severe acute malnutrition (Shahunja et al., 2021)			
13.	*Hippophae rhamnoides* L., Elaeagnaceae	Respiratory: infections (tonic, immune stimulant)	*China*: remarkable anti-fatigue activity (Ni et al., 2013;	Digestive: increased levels of appetite factors, leptin and neuropeptide	General: strongly associated with its dietary benefits for the immune system (Leahu et al., 2019)	General: significant immunomodulatory activity, specific activation	-

Nr.	Plant (Latin name, Family, English name)	Indications and biological activities reported in the present study	Ethnopediatric use in other countries previously reported in the literature	Clinical studies or case reports in children	Clinical studies or case reports in adults (for similar use as the one reported in the present study)	*In vivo* studies (supporting the use reported in the present study)	*In vitro* studies (supporting the use reported in the present study)
	(sea buckthorn)		Xiao et al., 2013)<br>*Mongolia:* sea buckthorn beverage for people like sportsmen, manual workers, children, and aged and pregnant women to enhance strength (Pokharel et al., 2021)	Y, gastric emptying and gastrointestinal digestive function, growth and development in children with functional dyspepsia (Xiao et al., 2013)		of cell-mediated immune response (Geetha et al., 2005)	

Nr.	Plant (Latin name, Family, English name)	Indications and biological activities reported in the present study	Ethnopediatric use in other countries previously reported in the literature	Clinical studies or case reports in children	Clinical studies or case reports in adults (for similar use as the one reported in the present study)	*In vivo* studies (supporting the use reported in the present study)	*In vitro* studies (supporting the use reported in the present study)
14.	*Hypericum perforatum* L., Hypericaceae (Saint John's wort)	Digestive: liver diseases, gallbladder dyskinesia (vulnerary)	*Poland*: treatment of burns (Coppock and Dziwenka, 2021)<br>*USA:* neurological diseases, depression, eczema, wart, cold, antioxidant (Lohse et al., 2006)	Ear: analgesic in ear pain associated with acute otitis media (Sarrell et al., 2001)<br>Psychological: attention deficiency disorder (Weber et al., 2008), depression (Hübner and Kirste, 2001)	Skin: vulnerary (Hajhashemi et al., 2018), wound healing (Samadi et al., 2010)	Digestive: hepatoprotective (Öztürk et al., 1992; Bayramoglu et al., 2014), gastroprotective (Zdunić et al., 2009), effective on inflammation, fibrosis, and necrosis in corrosive esophageal burns (Sümeli et al., 2022)<br>General: antioxidant in hyperlipidemic rats (Ghosian Moghaddam et al., 2016), antihyperglycemi c in induced	Anti-inflammatory (Hammer et al., 2010; Huang et al., 2011), antioxidant against hydrogen peroxide induced apoptosis in PC 12 cells (Zou et al., 2010)

Nr.	Plant (Latin name, Family, English name)	Indications and biological activities reported in the present study	Ethnopediatric use in other countries previously reported in the literature	Clinical studies or case reports in children	Clinical studies or case reports in adults (for similar use as the one reported in the present study)	*In vivo* studies (supporting the use reported in the present study)	*In vitro* studies (supporting the use reported in the present study)
						diabetic rats (S et al., 2011)	
15.	*Matricaria spp.* Asteraceae (chamomile)	Digestive: diarrhea, infantile colic Skin: skin and mucosa inflammation, eczema Eye: infection (relaxing, carminative, anti-inflammatory, anti-infective)	*Brazil*: flu, cough; stomach pain, nausea/vomiting, intestinal colic; calming/sedative; shingles (Gentil et al., 2010); intestinal cramps; flu symptoms; calm down the children (Alves and da Silva, 2003); rashes, eczema, and hemorrhoids; orally as a mild sedative or for	Digestive: calming in colic (Ghorat et al., 2017; Salehipoor et al., 2019; Savino et al., 2005; Sorme et al., 2019; Weizman et al., 1993), infantile colic (significantly more effective than simethicone (Martinelli et al., 2017), infantile colic(Sorme et al., 2019), antiinflammatory and spasmolytic effects on the stomach and duoden (Mann	Skin: benefits in atopic dermatitis, inflammatory dermatoses (Patzelt-Wenczler and Ponce-Pöschl, 2000), wound healing after dermabrasion of tattoos (Glowania et al., 1987) Psychological: relaxing, anxiolytic (Keefe et al., 2016; Ebrahimi et al., 2022) Digestive: oral mucositis (Carl and Emrich, 1991), reduces biofilm accumulation and gingival bleeding in patients with gingivitis (Goes et	Digestive: anti-Helicobacter pylori activity in rats (Patzelt-Wenczler and Ponce-Pöschl, 2000), antidiarrheal, antisecretory and antispasmodic activities in mice (Mehmood et al., 2015), reduces intestinal damage and visceral pain development in a rat model of DNBS-induced colitis(Parisio et al., 2019), protective effect against ethanol-induced gastric	Maintains the intestinal epithelial barrier integrity, anti-inflammatory in an in vitro model of intestinal inflammation (Weber et al., 2020; COCETTA et al., 2023), antibacterial on cariogenic species (Braga et al., 2021, 2022), antimicrobial (Ali, 2009; Křížkovská et al., 2023; Pastare et al., 2023), antiinflammatory (Singh et al., 2018; Asadi et al., 2020; Duan et al., 2022; Biltekin et al., 2023), antioxidant activity

Nr.	Plant (Latin name, Family, English name)	Indications and biological activities reported in the present study	Ethnopediatric use in other countries previously reported in the literature	Clinical studies or case reports in children	Clinical studies or case reports in adults (for similar use as the one reported in the present study)	*In vivo* studies (supporting the use reported in the present study)	*In vitro* studies (supporting the use reported in the present study)
			indigestion, diarrhea, and colic (Chavez, 2000) *UAE*: soothing infants (Abdulrazzaq et al., 2009) *USA*: colic, stomach ache, gastrointestinal; congestion, coughs, cold; calming, insomnia (Lohse et al., 2006); indigestion, diarrhea, and colic (Noonan et al., 2004); rashes, eczema; mild sedative or for indigestion, diarrhea and	and Staba, 1986), diarrhea (Aertgeerts et al., 1985; de la Motte et al., 1997) Respiratory: acute non-bacterial tonsillitis (Popovych et al., 2019), common cold (Javid et al., 2019) General: chemotherapy-induced neutropenia (Daneshfard et al., 2020), postoperative oedema after surgery for hypospadias	al., 2016), effective in minor aphthous stomatitis (Andishe Tadbir et al., 2015), relieve primary burning mouth syndrome (BMS) symptoms (Aitken-Saavedra et al., 2020)	mucosal lesions in rats (Cemek et al., 2010), oral wound healing in rats (Duarte et al., 2011) Psychological: sedative in rodents (Meneses et al., 2023) General: anti-inflammatory in carrageenan-induced paw in rats (Ortiz et al., 2017), anti-inflammatory and antioxidant in rats (Nargesi et al., 2018), wound healing, anti-inflammatory, antioxidant in rats (Jarrahi et al., 2010; Niknam et	and inhibitory effect on digestive enzymes (Franco et al., 2019)

Nr.	Plant (Latin name, Family, English name)	Indications and biological activities reported in the present study	Ethnopediatric use in other countries previously reported in the literature	Clinical studies or case reports in children	Clinical studies or case reports in adults (for similar use as the one reported in the present study)	*In vivo* studies (supporting the use reported in the present study)	*In vitro* studies (supporting the use reported in the present study)
			colic (Halsted, 2004)<br>*Germany*: digestive illnesses, colic (Nathan and Scholten, 1999)	(Zhang et al., 2021)<br>Psychological: slightly effective treatment for ADHD (Niederhofer, 2009)		al., 2021), antioxidant in polycystic ovary syndrome rats (Alahmadi et al., 2020), antioxidative in streptozotocin-induced diabetic rats (Cemek et al., 2008), digestive anti-nociceptive and anti-inflammatory effects in rats (Ortiz et al., 2016)<br>Skin: improves psoriazis-like lesions in a murine model (Chen et al., 2024), anti-infective in mice (Ali, 2009)	

Nr.	Plant (Latin name, Family, English name)	Indications and biological activities reported in the present study	Ethnopediatric use in other countries previously reported in the literature	Clinical studies or case reports in children	Clinical studies or case reports in adults (for similar use as the one reported in the present study)	In vivo studies (supporting the use reported in the present study)	In vitro studies (supporting the use reported in the present study)
16.	*Mentha spp.*, Lamiaceae (mint)	Digestive: diarrhea, bloating (relaxing, carminative, anti-infective, anti-flatulent)	*Brazil*: intestinal cramps; flu symptoms; calm down the children (Alves and da Silva, 2003)<br>*Turkey*: cough (Tural Büyük E et al., 2014)<br>*UAE*: soothing infants(Abdulrazzaq et al., 2009)<br>*USA*: stomachaches, indigestion, colic, overall, gastrointestinal; colds, cough, asthma, congestion; headache,	Digestive: benefits in infantile colic (equal efficiency with simethicone) (Alves et al., 2012), functional abdominal pain (Asgarshirazi et al., 2015), relieving symptoms of the irritable bowel syndrome (Kline et al., 2001), influences gut motility in children with functional abdominal pain(Shulman et al., 2022), as no significant effect on gut	Digestive: carminative (Amin, 2005), biliary disorders, dyspepsia, enteritis, flatulence, gastritis, intestinal colic, and spasms of the bile duct, gallbladder and gastrointestinal (GI) tract (McKay and Blumberg, 2006), carminative, sedation, immunity (Eid and Jaradat, 2020), gastrointestinal ailments (Wali et al., 2022), reduces colonic spasm during endoscopy (Leicester and Hunt, 1982), safe and effective therapy for pain and global symptoms in	Digestive: antidiarrheic in a castor oil-induced animal model (Mussarat et al., 2022), gastrointestinal and respiratory diseases in calves and piglets (Ayrle et al., 2016), anti-Helicobacter pilorii activity in rats (Zahid et al., 2020), antioxidant, antibacterial and intestinal anti-inflammatory effect in broilers (Moharreri et al., 2022), antioxidant and anti-inflammatory in an ulcerative	Antifungal (Tullio et al., 2023), antifungal alone or in combination with azole drugs (Tullio et al., 2019; Hsu et al., 2020), antimicrobial (Hussain et al., 2010; Magi et al., 2015; Badea et al., 2019; Wińska et al., 2019; Zahid et al., 2020; Hejna et al., 2021; Arbab et al., 2022; Kafa et al., 2022; Nakamura et al., 2022; Sarwar et al., 2022; Slimestad et al., 2022; Fazal et al., 2023), antioxidant (Sun et al., 2014; Arruda et al., 2017; Aldoghachi et al., 2021; Hejna et al., 2021; Slimestad et

Nr.	Plant (Latin name, Family, English name)	Indications and biological activities reported in the present study	Ethnopediatric use in other countries previously reported in the literature	Clinical studies or case reports in children	Clinical studies or case reports in adults (for similar use as the one reported in the present study)	In vivo studies (supporting the use reported in the present study)	In vitro studies (supporting the use reported in the present study)
			anxiety (Lohse et al., 2006)	microbiome in children with abdominal pain (Thapa et al., 2022), ameliorates pediatric nausea (Arruda and Yeh, 2019), ameliorates abdominal pain in children and adolescents (Van Tilburg and Felix, 2013), beneficial in chronic or recurrent abdominal pain in children (Banez, 2008)<br>Renal: may ameliorate postoperative urinary retention	adults with irritable bowel syndrome (Alammar et al., 2019), dyspepsia, irritable bowel syndrome, intraluminal spasmolytic agent during barium enemas or endoscopy (Koretz and Rotblatt, 2004)<br>Respiratory, Psychological, Digestive: headache, bronchitis, diarrhoea, dizziness, stomach ache vermifuge (Juárez-Vázquez et al., 2013), therapy for pain and global symptoms in adults with irritable bowel syndrome (Lacy et al., 2021)	colitis mouse model (Puppala et al., 2022)<br>Respiratory: anti-inflammatory in asthmatic mice (Su and Lin, 2022)<br>General: anti-inflammatory and nociceptive in rats (Mogosan et al., 2017), immunomodulatory and anti-inflammatory action in Schistosoma mansorni infected mice (Zaia et al., 2016), anti-nociceptive and inflammatory effect against acute and chronic	al., 2022; Chen et al., 2023), anti-inflammatory (Sun et al., 2014; Arruda et al., 2017; Hejna et al., 2021; Liu et al., 2021), antispasmodic on gastrointestinal guinea pig and rabbit preparates (Hills and Aaronson, 1991) and rat tracheal smooth muscle samples (Gao et al., 2011)

Nr.	Plant (Latin name, Family, English name)	Indications and biological activities reported in the present study	Ethnopediatric use in other countries previously reported in the literature	Clinical studies or case reports in children	Clinical studies or case reports in adults (for similar use as the one reported in the present study)	In vivo studies (supporting the use reported in the present study)	In vitro studies (supporting the use reported in the present study)
				(Fryatt and Bell, 2020)<br>Neurologic: relieves discomfort, nausea/vomiting, nervousness, and sleep disturbance in paediatric surgical patients (Czarnecki et al., 2022), acute therapy of tension-type headaches reviewed in (Göbel et al., 2016)<br>General: ameliorates dysmenorrhea in adolescents(Gutman et al., 2022) (Masoumi et al., 2016)	Respiratory: respiratory disease (Afzal et al., 2021), antioxidant and anti-inflammatory activity in epithelial cells of the human upper respiratory system (Gao et al., 2011)<br>Psychological: acute therapy of tension-type headaches reviewed (Göbel et al., 2016)<br>General: complementary treatment for chronic pain (Buckle, 1999), anti-inflammatory in patients with peri-implant mucositis (Alqutub et al., 2023)	inflammation in rodents (Atta and Alkofahi, 1998)	

Nr.	Plant (Latin name, Family, English name)	Indications and biological activities reported in the present study	Ethnopediatric use in other countries previously reported in the literature	Clinical studies or case reports in children	Clinical studies or case reports in adults (for similar use as the one reported in the present study)	In vivo studies (supporting the use reported in the present study)	In vitro studies (supporting the use reported in the present study)
				Respiratory: ameliorated cough in a 21-day-old newborn patient infected with SARS-CoV-2Click or tap here to enter text.			
17.	*Pimpinella anisum* L., Apiaceae (anise)	Digestive: intestinal cramps, infantile colic (relaxing, carminative)	*Brazil:* most commonly used for children (the specific indications for Pimpinella anisum are not mentioned) (Gentil et al., 2010)<br>*Iran*: attenuates infants gastrointestinal problems (Zargari, 1997)	Digestive: increases the weight gain in the preterm infant through a galactagogue effect on mothers (Khalili et al., 2023); significantly greater acceptance of breast-feeding following anise ingestion by mothers (Schaal	Digestive: carminative, antiseptic, digestive and a folk remedy to insomnia and constipation (Bisset and Wichtl, 1994), carminative (Ali-Shtayeh and Jamous, 2008), carminative during pregnancy (Ali-Shtayeh et al., 2015), laxative (Picon et al., 2010)<br>General: ameliorates primary	Muscular: attenuates spontaneous and agonist-induced uterine contraction in term-pregnant rats (Alotaibi, 2020)<br>General: analgesic in mice (Maciej Serda et al., 2006; Tas, 2009), analgesic and anti-inflammatory	Antispastic and relaxant on rat anococcygeus smooth muscle preparations stimulated with acetylcholine (Tirapelli et al., 2007), relaxant effects on the isolated tracheal muscle of the guineapig (Reiter and Brandt, 1985), relaxant on isolated tracheal chains of the guinea pig pre-contracted with

Nr.	Plant (Latin name, Family, English name)	Indications and biological activities reported in the present study	Ethnopediatric use in other countries previously reported in the literature	Clinical studies or case reports in children	Clinical studies or case reports in adults (for similar use as the one reported in the present study)	In vivo studies (supporting the use reported in the present study)	In vitro studies (supporting the use reported in the present study)
			Palestine: most commonly used for children (the specific indications for Pimpinella anisum are not mentioned)(Al-Jabi et al., 2021a); flatulence, relaxant, cures abdominal pain; cold (Ali-Shtayeh et al., 2015)<br>*Syria:* nutrition (prelacteal feed); pain relief, calming infants to sleep (Gentil et al., 2010; Al-Jabi et al., 2021a)	et al., 2000), promote oral feeding in immature newborn by odor stimulation (Cao Van et al., 2018)	dysmenorrhea (Khoda Karami et al., 2008)	(Maciej Serda et al., 2006)	methacholine (Boskabady and Ramazani-Assari, 2001), strong antibacterial activity against human pathogens (Al-Bayati, 2008; Al-Jabi et al., 2021b)

Nr.	Plant (Latin name, Family, English name)	Indications and biological activities reported in the present study	Ethnopediatric use in other countries previously reported in the literature	Clinical studies or case reports in children	Clinical studies or case reports in adults (for similar use as the one reported in the present study)	In vivo studies (supporting the use reported in the present study)	In vitro studies (supporting the use reported in the present study)
			Turkey: gas pains and digestive problems, constipation, nausea and vomiting (Gürol et al., 2019); relieves gas pains and prevent constipation, soothing effects (Giray Bozkaya et al., 2008; Abdulrazzaq et al., 2009)<br>*UAE*: soothing infants (Abdulrazzaq et al., 2009)				
18.	*Prunus avium* (L.) L., Rosaceae (cherry)	Renal: urinary tract infections (diuretic)	*India*: convulsion treatment	Musculoskeletal: inhibitory effects on osteoclastogenes	Renal: diuretic (Hooman et al., 2009; Prvulovic et	Renal: diuretic (Babotă et al., 2021);	Anti-inflammatory and antimicrobial (Abedini et al., 2020; Nunes et al., 2022),

Nr.	Plant (Latin name, Family, English name)	Indications and biological activities reported in the present study	Ethnopediatric use in other countries previously reported in the literature	Clinical studies or case reports in children	Clinical studies or case reports in adults (for similar use as the one reported in the present study)	In vivo studies (supporting the use reported in the present study)	In vitro studies (supporting the use reported in the present study)
			(Sheikh et al., 2022)	is in obese children and adolescents (Corbo et al., 2019)	al., 2011; Ademovic et al., 2017) General: antiinflammatory, antioxidant, antihypertensive (Arbizu et al., 2023)		anti-inflammatory and antiproliferative (Gonçalves et al., 2022), anti-inflammatory (Blazsó and Gábor, 1994)
;19.	*Raphanus raphanistrum subsp. sativus* (L.) Domin, Brassicaceae (black radish)	Respiratory: bronchitis (anti-inflammatory, calming, expectorant)	-	-	Respiratory: cough, throat inflammation (Nóra Papp et al., 2011), antitussive, expectorant and anti-asthmatic in clinical treatment of traditional Chinese medicine (Gao et al., 2022), antifever, antitussive and tonic in the Iranian traditional medicine (Afsharypuor and Balam, 2005)	Respiratory: increased expression of phase I and II detoxification enzymes in mice (N'Jai et al., 2012), protective effects against bleomycine-induced pulmonary fibrosis in a rat model (Asghari et al., 2015) Digestive: anti-inflammatory effects in dextran	Induction of phase I and II detoxification enzymes in HepG2 cells (Hanlon et al., 2007), anti-inflammatory through selective immunomodulatory effects (Jeon et al., 2020), antioxidant in HepG2 cells (Ahn et al., 2018), antioxidant (Lugasi et al., 1998), antioxidant in in human fetal lung fibroblasts (Luo et al., 2018), antibacterial (Beevi et al., 2009)

Nr.	Plant (Latin name, Family, English name)	Indications and biological activities reported in the present study	Ethnopediatric use in other countries previously reported in the literature	Clinical studies or case reports in children	Clinical studies or case reports in adults (for similar use as the one reported in the present study)	In vivo studies (supporting the use reported in the present study)	In vitro studies (supporting the use reported in the present study)
						sulfate sodium-induced ulcerative colitis in mice (Kim et al., 2020), anti-inflammatory in a non-alcoholic fatty liver disease rat model (Ahn et al., 2019), antioxidant and hepatoprotective effect against CCl4-induced liver injury in rats (Ahn et al., 2018), antioxidant in rats fed a lipid-rich diet (Lugasi et al., 2005), anti-inflammatory and antioxidant in fat-rich diet fed rats (Sipos et al., 2002)	

Nr.	Plant (Latin name, Family, English name)	Indications and biological activities reported in the present study	Ethnopediatric use in other countries previously reported in the literature	Clinical studies or case reports in children	Clinical studies or case reports in adults (for similar use as the one reported in the present study)	In vivo studies (supporting the use reported in the present study)	In vitro studies (supporting the use reported in the present study)
						General: attenuates cadmium-induced immunotoxicity in rats (Salah-Abbès et al., 2015)	
20.	*Rosa canina* L., Rosaceae (dog rose)	Respiratory: acute infective diseases (tonic, anti-infective, anti-inflammatory)	*Indonesia*: treatment of the cold and flu-like syndrome (Sakinah, 2021) *Poland*: dietary supplement (Łuczaj and Szymański, 2007) *Turkey*: dietary supplement and immune stimulant (Gulec et al., 2018)	Digestive: treatment of constipation in pediatric surgery practice (Krycky et al., n.d.) Psychological: attention-deficit/hyperactivity disorder treatment – ongoing trial (Golsorkhi et al., 2022)	Respiratory: treatment of respiratory problems (cough, bronchitis, and cold), flu, infectious ailments, vitamin C deficiency, anemia, vermifuge, tonic (Ayati et al., 2021) Skin: antiinfective, antiinflamatory on sunburned skin (Ala et al., 2018) General: immunostimulant (Tsioutsiou et al.,	Respiratory: antioxidant in a mouse model of paraquat-induced lung injury (Amirshahrokhi, 2020) General: antioxidant in a cyclophosphamide-induced testicular toxicity model in mice (Parandin et al., 2023), and in experimentally nephrolithiasic	Antioxidant (Tumbas et al., 2012; Mihaylova et al., 2019; Fetni et al., 2020; Moldovan et al., 2021; Nicolescu et al., 2022; Sabahi et al., 2022; Sallustio et al., 2022; Peña et al., 2023), antioxidant and cytoprotective in mouse primary splenocytes (Soualeh et al., 2017), antioxidant and anti-inflammatory (Wenzig et al., 2008),

Nr.	Plant (Latin name, Family, English name)	Indications and biological activities reported in the present study	Ethnopediatric use in other countries previously reported in the literature	Clinical studies or case reports in children	Clinical studies or case reports in adults (for similar use as the one reported in the present study)	In vivo studies (supporting the use reported in the present study)	In vitro studies (supporting the use reported in the present study)
					2019), immunomodulator, in HIV/AIDS patients (Koosha Paydary et al., 2012), decreased chemotaxis of peripheral blood PMNs serum acute phase protein CRP in healthy volunteers (Kharazmi and Winther, 1999) Musculoskeletal: antiinflammatory in patients with osteoarthritis (Warholm et al., 2003; Rein et al., 2004; Winther et al., 2005), positive clinical outcomes in patients with rheumatoid arthritis (Willich et al., 2010)	Wistar rats (Tayefi-Nasrabadi et al., 2012), immunomodulato r (Koosha Paydary et al., 2012), antiinflammatory (Deliorman Orhan et al., 2007; Lattanzio et al., 2011)	antiinflamatory (Kharazmi and Winther, 1999; Lattanzio et al., 2011; Nadpal et al., 2016; Hendrich et al., 2020; Soulimani et al., 2021; Wanes et al., 2021), antioxidant and antimicrobial (Jovanović et al., 2022), antimicrobial (Živković et al., 2015; Jafarirad et al., 2016; Gulbagca et al., 2019; Hendrich et al., 2020; Polumackanycz et al., 2020; Rovná et al., 2020), anti-inflammatory and anabolic effects on primary canine chondrocytes (Shakibaei et al., 2012), antibacterial

Nr.	Plant (Latin name, Family, English name)	Indications and biological activities reported in the present study	Ethnopediatric use in other countries previously reported in the literature	Clinical studies or case reports in children	Clinical studies or case reports in adults (for similar use as the one reported in the present study)	In vivo studies (supporting the use reported in the present study)	In vitro studies (supporting the use reported in the present study)
							(Kumarasamy et al., 2002; Quave et al., 2008), immunomodulator (Saaby et al., 2011; Koosha Paydary et al., 2012; Saaby and Nielsen, 2012)
21.	*Thymus serpyllum* L., Lamiaceae (wild thyme)	Respiratory: acute infective diseases (anti-infective, expectorant)	-	Respiratory: nocturnal cough and sleep quality (Farouk Abolwafa et al., 2023), thyme and ivy in reducing the severity of cough in children (Sharif et al., 2024), cough (Ludwig et al., 2015)	Respiratory: respiratory inflammations, immunostimulant, bronchitis, anti-asthmatic, expectorant (Mustafa et al., 2015)	Digestive: anti-inflammatory and immunomodulatory in a rodent model of intestinal colitis (Algieri et al., 2014)<br>General: antioxidant in spontaneously hypertensive rats (Mihailovic-Stanojevic et al., 2013)	Antioxidant (Egea et al., 2010; Komes et al., 2011; Hussain et al., 2013; Brezoiu et al., 2020; Pavlić et al., 2022), antimicrobial (Alzoreky and Nakahara, 2003; Brezoiu et al., 2020; Shanaida et al., 2021), antibacterial and antifungal , (Rasooli and Mirmostafa, 2002; Salaria et al., 2022)

Nr.	Plant (Latin name, Family, English name)	Indications and biological activities reported in the present study	Ethnopediatric use in other countries previously reported in the literature	Clinical studies or case reports in children	Clinical studies or case reports in adults (for similar use as the one reported in the present study)	In vivo studies (supporting the use reported in the present study)	In vitro studies (supporting the use reported in the present study)
22	*Tilia tomentosa* Moench., Malvaceae (silver linden)	Psychological: agitation, anxiety Respiratory: acute infective diseases with cough (relaxing, calming, sedative)	*Turkey:* gas pains and digestive problems, constipation, and nausea and vomiting; sore throat, cough (Gürol et al., 2019b); respiratory diseases (SARI et al., 2023b)	-	Psychological: anxiolytic (Gutiérrez et al., 2014) Respiratory: respiratory tract diseases and flu, tonsillitis and throat ache, bronchitis (Sargin et al., 2013), upper respiratory catarrh, common colds, irritable coughs, restlessness (Bradley, 2006), (Frezza et al., 2019), emollient, antitussive (Hritcu and Cioanca, 2016)	Psychological: anxiolytic (mice) (Viola et al., 1994; Turrini et al., 2020)	Sedative (mouse hippocampal neurons) (Allio et al., 2015), synaptosomal membranes from bovine cerebral cortex (Viola et al., 1994), antimicrobial (Sargin et al., 2013), anti-inflammatory, immunomodulator (Matsuda et al., 2002; Manuele et al., 2008; Georgiev et al., 2017), antiproliferative (Barreiro Arcos et al., 2006)
23	*Triticum aestivum L.*, Poaceae (wheat)	Skin: eczema (emollient)	-	Digestive: controlling gingival inflammation (Bello et al., 2020), decreases	Skin: moisturizing (Boisnic et al., 2019), venous ulcer healing (Romanelli et al., 2015), treatment of	Skin: anti-inflammatory (Funel et al., 2020; Alake et al., 2023), keratinocyte	Antiproliferative (Whent et al., 2012), antiinflammatory (Anson et al., 2011; Whent et al., 2012; Sanguigno et al.,

Nr.	Plant (Latin name, Family, English name)	Indications and biological activities reported in the present study	Ethnopediatric use in other countries previously reported in the literature	Clinical studies or case reports in children	Clinical studies or case reports in adults (for similar use as the one reported in the present study)	In vivo studies (supporting the use reported in the present study)	In vitro studies (supporting the use reported in the present study)
				the fecal bile acid excretion in juvenile ulcerative colitis (Ejderhamn et al., 2009), milk substitute for children with lactose intolerance (Suthutvoravut et al., 1984)<br>Blood: increases the Hb levels and the interval between transfusions in children with thalassemia major (Marwaha et al., 2004; Singh et al., 2010), reduces chemotherapy-	hyperkeratotic skin conditions (Ferrando, 1986)<br>Skin: anti-aging properties, cosmetic applications (Martins et al., 2022), cosmetic use in emulsions (Akhtar and Yazan, 2008), treatment of severe atopic dermatitis-complicated cataracts (Niwa et al., 1998)<br>General: antioxidant (Anson et al., 2011), melasma treatment (Bavarsad et al., 2021)	healing (Morretta et al., 2022), tissue regenerative (Tito et al., 2020), treatment of atopic dermatitis-like skin lesions in mice (Lee et al., 2023), atopic dermatitis lesions healer (Lee et al., 2018), skin wound healing (Sui et al., 2020)	2018; YIN et al., 2019; Lee et al., 2023; Vinci et al., 2023; Zargar et al., 2023), antioxidant (Colombo et al., 2009; Senol et al., 2012; YIN et al., 2019; Lee et al., 2023; Spaggiari et al., 2023; Vinci et al., 2023), antimicrobial (Spaggiari et al., 2023), antibacterial (Bassyouni et al., 2022), skin wound healing (Sui et al., 2020), attenuates chemokine expression in human keratinocytes (Lee et al., 2018), protective effects in ultraviolet-stressed human keratinocyte cells

Nr.	Plant (Latin name, Family, English name)	Indications and biological activities reported in the present study	Ethnopediatric use in other countries previously reported in the literature	Clinical studies or case reports in children	Clinical studies or case reports in adults (for similar use as the one reported in the present study)	In vivo studies (supporting the use reported in the present study)	In vitro studies (supporting the use reported in the present study)
				induced febrile neutropenia in pediatric cancer patients (Garami et al., 2004)			(Arda and Doğanlar, 2022), enhances the proliferation and hydration of human keratinocytes (Hahm et al., 2021)
24.	*Urtica dioica* L., Urticaceae (common nettle)	General: weakness Blood: anaemia (tonic)	*Ecuador*: tonic, nutritional (Roche et al., 2017) *Spain:* antitussive (for children) (Rigat et al., 2015)	General: benefits in weakness and anemia in children with cancer (Gözüm et al., 2007; Karali et al., 2012; Roche et al., 2017);	General: immunity, metabolic and nutritional, anemia (Matejić et al., 2020), antioxidant, reviewed in (Jaiswal and Lee, 2022), immunomodulator in human lung cancer (Timoshenko et al., 1996), immunomodulator in HIV/AIDS patients (Koosha Paydary et al., 2012), galactagogue for breast-feeding mothers with	General: antioxidant, reviewed in (Jaiswal and Lee, 2022), immunostimulant in DHEA-induced mice (Shamsi et al., 2023), immunostimulant in BALB/c mice infected with Leishmania major (Badirzadeh et al., 2020), anti-anemia and immunostimulant in CCl4-Treated	Anti-viral effects in rotavirus infection (Knipping et al., 2012), and SARS-CoV-2 (Sabzian-Molaei et al., 2022, 2023), antioxidant and antibacterial (Dağlıoğlu et al., 2023; Khan et al., 2023), antioxidant (Mihaylova et al., 2019; Jaiswal and Lee, 2022; Uğur and Güzel, 2023), anti-leishmanial effect (Badirzadeh et al., 2020),

Nr.	Plant (Latin name, Family, English name)	Indications and biological activities reported in the present study	Ethnopediatric use in other countries previously reported in the literature	Clinical studies or case reports in children	Clinical studies or case reports in adults (for similar use as the one reported in the present study)	In vivo studies (supporting the use reported in the present study)	In vitro studies (supporting the use reported in the present study)
					preterm babies (Özalkaya et al., 2018), nutrition (Bhusal et al., 2022) Blood: hematopoiesis, strengthening the organism (Sõukand and Kalle, 2013), tonic, anemia (Jarić et al., 2007), (Stinging Nettle, 2021)	Rats (Meral and Kanter, 2003), immunomodulator (Koosha Paydary et al., 2012)	immunomodulatory effects (Akbay et al., 2003), anti-anemia (Lukačovičová and Havránek, 2015), nutrition (Adhikari et al., 2016; Chakravartula et al., 2021; Radha et al., 2021; Montoya-Arroyo et al., 2022), anti-inflammatory and anabolic effects on primary canine chondrocytes (Shakibaei et al., 2012), immunomodulator (Koosha Paydary et al., 2012)
25.	*Viola tricolor* L., Violaceae (heartsease)	General: allergies (anti-allergic)	*Iran*: functional constipation (Tavassoli et al., 2021)	Digestive: functional constipation in children	General: antiallergic (Klövekorn et al., 2007), anti-inflammatory,	General: anti-inflammatory in male Wistar rats	Immunosuppressant (aqueous Viola extract inhibited proliferation of

Nr.	Plant (Latin name, Family, English name)	Indications and biological activities reported in the present study	Ethnopediatric use in other countries previously reported in the literature	Clinical studies or case reports in children	Clinical studies or case reports in adults (for similar use as the one reported in the present study)	In vivo studies (supporting the use reported in the present study)	In vitro studies (supporting the use reported in the present study)
			Poland: seborrhoea of the scalp in nursing infants (Witkowska-Banaszczak et al., 2005) *Ukraine*: expectorant infusion (Herasymova et al., 2022)	(Tavassoli et al., 2021) Skin: an open-label trial of 27 children using a homeopathic cream of Oregon grape root (M. aquifolium), pansy (Viola tricolor hortensis), and gotu kola (Centella asiatica) revealed complete resolution of atopic dermatitis in 6 children and marked improvement in 16.73 (Rakel, 2018)	antiallergic (Harati et al., 2018) (Tobyn et al., 2011)	(Toiu et al., 2007), anti-inflammatory in a model of sunburn in rats (Piana et al., 2013), and in a mouse model of chronic asthma (Harati et al., 2018)	cultured activated lymphocytes by reducing IL-2, IFN-γ and TNF-α production)(Hellinger et al., 2014), antimicrobial activity (Witkowska-Banaszczak et al., 2005b), antioxidant (Vukics et al., 2008; Koike et al., 2015)

Legend- #- (ethno)pediatric use different from the one reported in the present study; PF- polyherbal formulation including the respective plant; MT- mixed treatment: plant combined

References

A. ACORDA, J., C. MANGUBAT, I. Y. E., and P. DIVINA, B. (2019). Evaluation of the in vivo efficacy of pumpkin (<i>Cucurbita pepo</i>) seeds against gastrointestinal helminths of chickens. *Turk J Vet Anim Sci* 43, 206–211. doi: 10.3906/vet-1807-39

Abas, A.-S. M., and Elagib, S. M. (2021). Antiparasitic activity of aqueous extract of Anethum graveolens against Entamoeba histolytica: In vitro and in vivo study. *Biocatal Agric Biotechnol* 34, 102026. doi: 10.1016/j.bcab.2021.102026

Abd El-Galil, M. A. A., and Aboelhadid, S. M. (2012). Trials for the control of trichodinosis and gyrodactylosis in hatchery reared Oreochromis niloticus fries by using garlic. *Vet Parasitol* 185, 57–63. doi: 10.1016/j.vetpar.2011.10.035

Abd-ELrahman, S. M., Mohamed, S. A.-A., Mohamed, S. E., El-Khadragy, M. F., Dyab, A. K., Hamad, N., et al. (2022). Comparative Effect of Allicin and Alcoholic Garlic Extract on the Morphology and Infectivity of Eimeria tenella Oocysts in Chickens. *Animals (Basel)* 12. doi: 10.3390/ani12223185

Abdo, S. M., Ibrahim Ghallab, M. M., Zaghloul, M. S., and Elhadad, H. (2023). Efficacy of Nitazoxanide, Nitazoxanide-Garlic Combination and Tinidazole in Treatment of Giardia duodenalis and Blastocystis hominis: Coproscopic Assessment. *Iran J Parasitol*. doi: 10.18502/ijpa.v18i1.12379

Abdulrazzaq, Y. M., Kendi, A. Al, and Nagelkerke, N. (2009). Soothing methods used to calm a baby in an Arab country. *Acta Paediatr* 98, 392–396. doi: 10.1111/J.1651-2227.2008.01029.X

Abedini, A., Colin, M., Hubert, J., Charpentier, E., Angelis, A., Bounasri, H., et al. (2020). Abundant Extractable Metabolites from Temperate Tree Barks: The Specific Antimicrobial Activity of Prunus Avium Extracts. *Antibiotics* 9, 111. doi: 10.3390/antibiotics9030111

Abhishek Anand, DASS, L. L., SHARMA, A. K., and Abhishek Anand (2012). Wound healing potential of sunflower and olive oils in goats. *Indian Journal of Small Ruminants* 18, 225–228.

Ademovic, Z., Hodzic, S., Halilic-Zahirovic, Z., Husejnagic, D., Dzananovic, J., Saric-Kundalic, B., et al. (2017). Phenolic compounds, antioxidant and antimicrobial properties of the wild cherry (Prunus avium L.) stem. *Acta Periodica Technologica*, 1–13. doi: 10.2298/APT1748001A

Adhikari, B. M., Bajracharya, A., and Shrestha, A. K. (2016). Comparison of nutritional properties of Stinging nettle (Urtica dioica) flour with wheat and barley flours. *Food Sci Nutr* 4, 119–124. doi: 10.1002/FSN3.259

Adib-Hajbaghery, M., Mahmoudi, M., and Mashaiekhi, M. (2014). The effects of Bentonite and Calendula on the improvement of infantile diaper dermatitis. *J Res Med Sci* 19, 314–8.

Adkison, J. D., Bauer, D. W., and Chang, T. (2010). The Effect of Topical Arnica on Muscle Pain. *Annals of Pharmacotherapy* 44, 1579–1584. doi: 10.1345/aph.1P071

Afsharypuor, S., and Balam, M. H. (2005). Volatile Constituents of *Raphanus sativus* L. var. *niger* Seeds. *Journal of Essential Oil Research* 17, 440–441. doi: 10.1080/10412905.2005.9698955

Afzal, S., Ahmad, H. I., Jabbar, A., Tolba, M. M., Abouzid, S., Irm, N., et al. (2021). Use of Medicinal Plants for Respiratory Diseases in Bahawalpur, Pakistan. *Biomed Res Int* 2021. doi: 10.1155/2021/5578914

Ahmed, A. S. M. N. U., Saha, S. K., Chowdhury, M. A. K. A., Law, P. A., Black, R. E., Santosham, M., et al. (2007). Acceptability of massage with skin barrier-enhancing emollients in young neonates in Bangladesh. *J Health Popul Nutr* 25, 236–40.

Ahn, M., Kim, J., Choi, Y., Ekanayake, P., Chun, J., Yang, D., et al. (2019). Fermented black radish (*Raphanus sativus* L. var. *niger*) attenuates methionine and choline deficient diet-induced nonalcoholic fatty liver disease in mice. *Food Sci Nutr* 7, 3327–3337. doi: 10.1002/fsn3.1200

Ahn, M., Kim, J., Hong, S., Kim, J., Ko, H., Lee, N. H., et al. (2018). Black Radish (Raphanus sativus L. var. niger) Extract Mediates Its Hepatoprotective Effect on Carbon Tetrachloride-Induced Hepatic Injury by Attenuating Oxidative Stress. *https://home.liebertpub.com/jmf* 21, 866–875. doi: 10.1089/JMF.2017.4102

Aitken-Saavedra, J., Chaves Tarquinio, S. B., De Oliveira Da Rosa, W. L., Fernandes Da Silva, A., Almeida MacHado, B. M. E., Santos Castro, I., et al. (2020). Effect of a Homemade Salivary Substitute Prepared Using Chamomile (Matricaria chamomilla L.) Flower and Flax (Linum usitatissimum L.) Seed to Relieve Primary Burning Mouth Syndrome: A Preliminary Report. *https://home.liebertpub.com/acm* 26, 799–806. doi: 10.1089/ACM.2019.0478

Akbaş, A., Emeksiz, Z. Ş., Yorulmaz, A., Hayran, Y., Kılınç, F., Yakut, H. I., et al. (2022). Complementary and alternative treatment methods practiced by parents in pediatric cases diagnosed with atopic dermatitis. *Journal of Dermatological Treatment* 33, 3014–3021. doi: 10.1080/09546634.2022.2097159

Akbay, P., Basaran, A. A., Undeger, U., and Basaran, N. (2003). In vitro immunomodulatory activity of flavonoid glycosides from Urtica dioica L. *Phytotherapy Research* 17, 34–37. doi: 10.1002/PTR.1068

Akhtar, N., and Yazan, Y. (2008). Formulation and in-vivo evaluation of a cosmetic multiple emulsion containing vitamin C and wheat protein. *Pak J Pharm Sci* 21, 45–50. Available at: https://pubmed.ncbi.nlm.nih.gov/18166519/ (Accessed June 9, 2023).

Akrami, R., Gharaei, A., Mansour, M. R., and Galeshi, A. (2015). Effects of dietary onion (Allium cepa) powder on growth, innate immune response and hemato–biochemical parameters of beluga (Huso huso Linnaeus, 1754) juvenile. *Fish Shellfish Immunol* 45, 828–834.

Ala, A., Ebrahimi Bakhtavar, H., Shams Vahdati, S., Rahmani, F., Azargoun, M., and Ebrahimi Bakhtavar, H. (2018). Effects of Silver Sulfadiazine and Adibderm® Herbal Ointments in Treatment of Patients with Second Degree Burns: A Randomized Clinical Trial. *Trauma Mon* 23. doi: 10.5812/traumamon.13396

Alahmadi, A. A., Alzahrani, A. A., Ali, S. S., Alahmadi, B. A., Arab, R. A., and El-Shitany, N. A. E. A. (2020). Both Matricaria chamomilla and Metformin Extract Improved the Function and Histological Structure of Thyroid Gland in Polycystic Ovary Syndrome Rats through Antioxidant Mechanism. *Biomolecules 2020, Vol. 10, Page 88* 10, 88. doi: 10.3390/BIOM10010088

Alake, S. E., Lightfoot, S., Wozniak, K., Lin, D., Chowanadisai, W., Smith, B. J., et al. (2023). Wheat Germ Supplementation Reduces Inflammation and Gut Epithelial Barrier Dysfunction in Female Interleukin-10 Knockout Mice Fed a Pro-Atherogenic Diet. *J Nutr* 153, 870–879. doi: 10.1016/J.TJNUT.2023.01.008

Alammar, N., Wang, L., Saberi, B., Nanavati, J., Holtmann, G., Shinohara, R. T., et al. (2019). The impact of peppermint oil on the irritable bowel syndrome: A meta-analysis of the pooled clinical data. *BMC Complement Altern Med* 19, 1–10. doi: 10.1186/S12906-018-2409-0/TABLES/3

Al-Bayati, F. A. (2008). Synergistic antibacterial activity between Thymus vulgaris and Pimpinella anisum essential oils and methanol extracts. *J Ethnopharmacol* 116, 403–406. doi: 10.1016/J.JEP.2007.12.003

Aldoghachi, F. E. H., Noor Al-Mousawi, U. M., and Shari, F. H. (2021). Antioxidant Activity of Rosmarinic Acid Extracted and Purified from Mentha piperita. *Arch Razi Inst* 76, 1279–1287. doi: 10.22092/ARI.2021.356072.1770

Al-Essa, M. K., Shafagoj, Y. A., Mohammed, F. I., and Afifi, F. U. (2010). Relaxant effect of ethanol extract of *Carum carvi* on dispersed intestinal smooth muscle cells of the guinea pig. *Pharm Biol* 48, 76–80. doi: 10.3109/13880200903046161

Alexandrovich, Irina., Rakovitskaya, O., Kolmo, E., Sidorova, T., and Shushunov, S. (2003). The effect of fennel (Foeniculum vulgare) seed oil emulsion in infantile colic: A randomized, placebo-controlled study. *Alternative Therapies in Health and Medicine; Aliso Viejo* 9, 58–61.

Algieri, F., Rodriguez-Nogales, A., Garrido-Mesa, N., Zorrilla, P., Burkard, N., Pischel, I., et al. (2014). Intestinal anti-inflammatory activity of the Serpylli herba extract in experimental models of rodent colitis. *J Crohns Colitis* 8, 775–788. doi: 10.1016/J.CROHNS.2013.12.012

Alhawiti, A. O., Toulah, F. H., and Wakid, M. H. (2019). Anthelmintic Potential of Cucurbita pepo Seeds on Hymenolepis nana. *Acta Parasitol* 64, 276–281. doi: 10.2478/s11686-019-00033-z

Ali, E. H. (2009). In vivo and In vitro Antimicrobial Activity of Flower and Callus Extracts of Matricaria chamomilla L. in The Treatment of Experimentally-Induced Skin. Ministry of Higher Education PhD Thesis.

Ali-Shtayeh, M. S., and Jamous, R. M. (2008). *Traditional Arabic Palestinian Herbal Medicine, TAPHM.* Nablus, Palestine: Nablus, Palestine: Biodiversity & Environmental Research Center BERCISBN: 978 9950 324 04 6.

Ali-Shtayeh, M. S., Jamous, R. M., and Jamous, R. M. (2015). Plants used during pregnancy, childbirth, postpartum and infant healthcare in Palestine. *Complement Ther Clin Pract* 21, 84–93. doi: 10.1016/J.CTCP.2015.03.004

Al-Jabi, S. W., Khader, M., Hamarsha, I., Atallh, D., Bani-Odeh, S., Daraghmeh, A., et al. (2021a). Complementary and alternative medicine use among pediatrics in Palestine: a cross-sectional study. *BMC Pediatr* 21, 1–9. doi: 10.1186/S12887-021-02985-6/TABLES/7

Al-Jabi, S. W., Khader, M., Hamarsha, I., Atallh, D., Bani-Odeh, S., Daraghmeh, A., et al. (2021b). Complementary and alternative medicine use among pediatrics in Palestine: a cross-sectional study. *BMC Pediatr* 21, 1–9. doi: 10.1186/S12887-021-02985-6/TABLES/7

Allio, A., Calorio, C., Franchino, C., Gavello, D., Carbone, E., and Marcantoni, A. (2015). Bud extracts from Tilia tomentosa Moench inhibit hippocampal neuronal firing through GABAA and benzodiazepine receptors activation. *J Ethnopharmacol* 172, 288–296. doi: 10.1016/J.JEP.2015.06.016

Alonso-Castro, A. J., Ruiz-Noa, Y., Martínez-de la Cruz, G. C., Ramírez-Morales, M. A., Deveze-Álvarez, M. A., Escutia-Gutiérrez, R., et al. (2022). Factors and Practices Associated with Self-Medicating Children among Mexican Parents. *Pharmaceuticals* 15, 1078. doi: 10.3390/ph15091078

Alotaibi, M. F. (2020). Pimpinella anisum extract attenuates spontaneous and agonist-induced uterine contraction in term-pregnant rats. *J Ethnopharmacol* 254. doi: 10.1016/J.JEP.2020.112730

Alqutub, M. N., Alhumaidan, A. A., Alali, Y., Al-Aali, K. A., Javed, F., Vohra, F., et al. (2023). Comparison of the postoperative anti-inflammatory efficacy of chlorhexidine, saline rinses and herbal mouthwashes after mechanical debridement in patients with peri-implant mucositis: A randomized controlled trial. *Int J Dent Hyg* 21, 203–210. doi: 10.1111/IDH.12582

Alves, A. R., and da Silva, M. J. (2003). O uso da fitoterapia no cuidado de crianças com até cinco anos em área central e periférica da cidade de São Paulo. *Revista da Escola de Enfermagem da USP* 37, 85–91. doi: 10.1590/S0080-62342003000400010

Alzoreky, N. S., and Nakahara, K. (2003). Antibacterial activity of extracts from some edible plants commonly consumed in Asia. *Int J Food Microbiol* 80, 223–230. doi: 10.1016/S0168-1605(02)00169-1

Amin, G. R. (2005). *Popular Medicinal Plants of Iran*. Tehran, Iran: Tehran University of Medical Science Press.

Amirshahrokhi, K. (2020). The Effect of Rosa canina Extract Against Paraquat-induced Lung Injury. *Journal of Ardabil University of Medical Sciences* 19, 400–409. doi: 10.29252/jarums.19.4.400

Andishe Tadbir, A., Pourshahidi, S., Ebrahimi, H., Hajipour, Z., Memarzade, M. R., and Shirazian, S. (2015). The effect of Matricaria chamomilla (chamomile) extract in Orabase on minor aphthous stomatitis, a randomized clinical trial. *J Herb Med* 5, 71–76. doi: 10.1016/J.HERMED.2015.05.001

Andrianova, I. V, Sobenin, I. A., Sereda, E. V, Borodina, L. I., and Studenikin, M. I. (2003). [Effect of long-acting garlic tablets “allicor” on the incidence of acute respiratory viral infections in children]. *Ter Arkh* 75, 53–56.
Anson, N. M., Aura, A. M., Selinheimo, E., Mattila, I., Poutanen, K., Van Den Berg, R., et al. (2011). Bioprocessing of Wheat Bran in Whole Wheat Bread Increases the Bioavailability of Phenolic Acids in Men and Exerts Antiinflammatory Effects ex Vivo. *J Nutr* 141, 137–143. doi: 10.3945/JN.110.127720
Arbab, S., Ullah, H., Bano, I., Li, K., Ul Hassan, I., Wang, W., et al. (2022). Evaluation of in vitro antibacterial effect of essential oil and some herbal plant extract used against mastitis pathogens. *Vet Med Sci* 8, 2655–2661. doi: 10.1002/VMS3.959
Arbizu, S., Mertens-Talcott, S. U., Talcott, S., and Noratto, G. D. (2023). Dark Sweet Cherry (Prunus avium) Supplementation Reduced Blood Pressure and Pro-Inflammatory Interferon Gamma (IFNγ) in Obese Adults without Affecting Lipid Profile, Glucose Levels and Liver Enzymes. *Nutrients* 15, 681. doi: 10.3390/nu15030681
Arda, H., and Doğanlar, O. (2022). Stress-induced miRNAs isolated from wheat have a unique therapeutic potential in ultraviolet-stressed human keratinocyte cells. *Environ Sci Pollut Res Int* 29, 17977–17996. doi: 10.1007/S11356-021-17039-8
Arruda, J., and Yeh, A. M. (2019). Integrative approach to pediatric nausea. *Pediatr Ann* 48, e236–e242. doi: 10.3928/19382359-20190517-01
Arruda, M. O., Mendes, S. J. F., Teixeira, S. A., De Mesquita, L. S. S., De Sousa Ribeiro, M. N., Galvão, S. D. S. L., et al. (2017). The Hydroalcoholic Extract Obtained from Mentha piperita L. Leaves Attenuates Oxidative Stress and Improves Survival in Lipopolysaccharide-Treated Macrophages. *J Immunol Res* 2017. doi: 10.1155/2017/2078794
Asadi, Z., Ghazanfari, T., and Hatami, H. (2020). Anti-inflammatory effects of matricaria chamomilla extracts on BALB/c mice macrophages and lymphocytes. *Iran J Allergy Asthma Immunol* 19, 63–73. doi: 10.18502/IJAAI.V19I(S1.R1).2862
Asghari, M. H., Hobbenaghi, R., Nazarizadeh, A., and Mikaili, P. (2015). Hydro-alcoholic extract of Raphanus sativus L. var niger attenuates bleomycin-induced pulmonary fibrosis via decreasing transforming growth factor β1 level. *Res Pharm Sci* 10, 429. Available at: /pmc/articles/PMC4691963/ (Accessed June 18, 2023).
Ashour, D. S., Deyab, F. A., Eliwa, K. F., and El-Kowrany, S. I. (2023). Anthelmintic potential of sulphonamides and Cucurbita pepo seeds extract on Heterophyes heterophyes experimentally infected mice. *Journal of Parasitic Diseases* 47, 697–706. doi: 10.1007/s12639-023-01620-8
Atta, A. H., and Alkofahi, A. (1998). Anti-nociceptive and anti-inflammatory effects of some Jordanian medicinal plant extracts. *J Ethnopharmacol* 60, 117–124. doi: 10.1016/S0378-8741(97)00137-2

Ayati, Z., Ramezani, M., Amiri, M. S., Sahebkar, A., and Emami, S. A. (2021). Genus Rosas : A Review of Ethnobotany, Phytochemistry and Traditional Aspects According to Islamic Traditional Medicine (ITM). *Adv Exp Med Biol* 1308, 353–401. doi: 10.1007/978-3-030-64872-5_23/COVER

Ayivi, B., and Dan, V. (1990). [Accidents linked to traditional treatment of convulsions of infants and children in Benin]. *Dev Sante*, 25–29.

Ayrle, H., Mevissen, M., Kaske, M., Nathues, H., Gruetzner, N., Melzig, M., et al. (2016). Medicinal plants – prophylactic and therapeutic options for gastrointestinal and respiratory diseases in calves and piglets? A systematic review. *BMC Veterinary Research 2016 12:1* 12, 1–31. doi: 10.1186/S12917-016-0714-8

Babotă, M., Voştinaru, O., Păltinean, R., Mihali, C., Dias, M. I., Barros, L., et al. (2021). Chemical Composition, Diuretic, and Antityrosinase Activity of Traditionally Used Romanian Cerasorum stipites. *Front Pharmacol* 12. doi: 10.3389/fphar.2021.647947

Badea, M. L., Iconaru, S. L., Groza, A., Chifiriuc, M. C., Beuran, M., and Predoi, D. (2019). Peppermint Essential Oil-Doped Hydroxyapatite Nanoparticles with Antimicrobial Properties. *Molecules 2019, Vol. 24, Page 2169* 24, 2169. doi: 10.3390/MOLECULES24112169

Badgujar, S. B., Patel, V. V., and Bandivdekar, A. H. (2014). *Foeniculum vulgare* Mill: A Review of Its Botany, Phytochemistry, Pharmacology, Contemporary Application, and Toxicology. *Biomed Res Int* 2014, 1–32. doi: 10.1155/2014/842674

Badirzadeh, A., Heidari-Kharaji, M., Fallah-Omrani, V., Dabiri, H., Araghi, A., and Chirani, A. S. (2020). Antileishmanial activity of Urtica dioica extract against zoonotic cutaneous leishmaniasis. *PLoS Negl Trop Dis* 14, e0007843. doi: 10.1371/JOURNAL.PNTD.0007843

Bahmani, M., Zargaran, A., and Rafieian-Kopaei, M. (2014). Identification of medicinal plants of Urmia for treatment of gastrointestinal disorders. *Revista Brasileira de Farmacognosia* 24, 468–480. doi: 10.1016/j.bjp.2014.08.001

Băieş, M.-H., Cotuţiu, V.-D., Spînu, M., Mathe, A., Cozma-Petruț, A., Miere, D., et al. (2023). The Effects of Coriandrum sativum L. and Cucurbita pepo L. against Gastrointestinal Parasites in Swine: An In Vivo Study. *Microorganisms* 11, 1230. doi: 10.3390/microorganisms11051230

Băieş, M.-H., Gherman, C., Boros, Z., Olah, D., Vlase, A.-M., Cozma-Petruț, A., et al. (2022). The Effects of Allium sativum L., Artemisia absinthium L., Cucurbita pepo L., Coriandrum sativum L., Satureja hortensis L. and Calendula officinalis L. on the Embryogenesis of Ascaris suum Eggs during an In Vitro Experimental Study. *Pathogens* 11. doi: 10.3390/pathogens11091065

Banez, G. A. (2008). Chronic abdominal pain in children: what to do following the medical evaluation. *Curr Opin Pediatr* 20, 571–575. doi: 10.1097/MOP.0B013E32830C6EF1

Baraneţchi, C., Segal, B., and Segal, R. (1985). [Administration of a water-alcohol extract of the fir tree (Abies alba) as an aerosol in respiratory tract diseases]. *Rev Med Chir Soc Med Nat Iasi* 89, 123–124.
Barbu, I. A., Ciorîță, A., Carpa, R., Moț, A. C., Butiuc-Keul, A., and Pârvu, M. (2023). Phytochemical Characterization and Antimicrobial Activity of Several Allium Extracts. *Molecules* 28, 3980. doi: 10.3390/molecules28103980
BÂRLĂ, G. F., POROCH-SERIŢAN, M., SĂNDULEAC, E., and CIORNEI, S. E. (2016). Antioxidant activity and total phenolic content in Allium ursinum and Ranunculus Ficaria. *Food and Environment Safety Journal* 13.
Barreiro Arcos, M. L., Cremaschi, G., Werner, S., Coussio, J., Ferraro, G., and Anesini, C. (2006). Tilia cordata Mill. Extracts and scopoletin (isolated compound): differential cell growth effects on lymphocytes. *Phytother Res* 20, 34–40. doi: 10.1002/PTR.1798
Bassyouni, R. H., Kamel, Z., Algameel, A. A., Ismail, G., and Gaber, S. N. (2022). In-vitro determination of antimicrobial activities of Eruca sativa seed oil against antibiotic-resistant gram-negative clinical isolates from neonates: a future prospect. *BMC Complement Med Ther* 22. doi: 10.1186/S12906-022-03710-1
Bavarsad, N., Mapar, M. A., Safaezadeh, M., and Latifi, S. M. (2021). A double-blind, placebo-controlled randomized trial of skin-lightening cream containing lycopene and wheat bran extract on melasma. *J Cosmet Dermatol* 20, 1795–1800. doi: 10.1111/JOCD.13799
Bayramoglu, G., Bayramoglu, A., Engur, S., Senturk, H., Ozturk, N., and Colak, S. (2014). The hepatoprotective effects of Hypericum perforatum L. on hepatic ischemia/reperfusion injury in rats. *Cytotechnology* 66, 443–448. doi: 10.1007/s10616-013-9595-x
Beevi, S. S., Mangamoori, L. N., Dhand, V., and Ramakrishna, D. S. (2009). Isothiocyanate Profile and Selective Antibacterial Activity of Root, Stem, and Leaf Extracts Derived from Raphanus sativus L. *https://home.liebertpub.com/fpd* 6, 129–136. doi: 10.1089/FPD.2008.0166
Beshay, E. V. N., Rady, A. A., Afifi, A. F., and Mohamed, A. H. (2019). Schistosomicidal, antifibrotic and antioxidant effects of Cucurbita pepo L. seed oil and praziquantel combined treatment for Schistosoma mansoni infection in a mouse model. *J Helminthol* 93, 286–294. doi: 10.1017/S0022149X18000317
Bhusal, K. K., Magar, S. K., Thapa, R., Lamsal, A., Bhandari, S., Maharjan, R., et al. (2022). Nutritional and pharmacological importance of stinging nettle (Urtica dioica L.): A review. *Heliyon* 8, e09717. doi: 10.1016/J.HELIYON.2022.E09717
Biltekin, S. N., Karadağ, A. E., Demirci, F., and Demirci, B. (2023). In Vitro Anti-Inflammatory and Anticancer Evaluation of Mentha spicata L. and Matricaria chamomilla L. Essential Oils. *ACS Omega* 8, 17143–17150. doi: 10.1021/ACSOMEGA.3C01501
Bisset, N. G., and Wichtl, M. (1994). *Herbal Drugs and Phytopharmaceuticals*. Stuttgart: Medpharm GmbH Scientific Publishers, Stuttgart, CRC Press, Boca Raton.

Biswas, S. J., Bhattacharjee, N., and Khuda-Bukhsh, A. R. (2008). Efficacy of a plant extract (Chelidonium majus L.) in combating induced hepatocarcinogenesis in mice. *Food and Chemical Toxicology* 46, 1474–1487. doi: 10.1016/j.fct.2007.12.009

Blazsó, G., and Gábor, M. (1994). Anti-inflammatory effects of cherry (Prunus avium L.) stalk extract. *Pharmazie* 49, 540–1.

Bombicz, M., Priksz, D., Varga, B., Gesztelyi, R., Kertesz, A., Lengyel, P., et al. (2016). Anti-Atherogenic Properties of Allium ursinum Liophylisate: Impact on Lipoprotein Homeostasis and Cardiac Biomarkers in Hypercholesterolemic Rabbits. *Int J Mol Sci* 17, 1284. doi: 10.3390/ijms17081284

Boros, Z., Baies, M. H., Gherman, C., and Cozma, V. (2021). The effects of Artemisia absinthium (wormwood), Allium sativum (garlic), Cucurbita pepo (pumpkin), and Coriandrum sativum (coriander) on Trichinella spiralis and Trichinella britovi larvae, in vitro study. *Sci. Parasitol,* 22, 70–78.

Boskabady, M. H., and Ramazani-Assari, M. (2001). Relaxant effect of Pimpinella anisum on isolated guinea pig tracheal chains and its possible mechanism(s). *J Ethnopharmacol* 74, 83–88. doi: 10.1016/S0378-8741(00)00314-7

Bradley, P. ed. (2006). *British Herbal Compendium: A Handbook of Scientific Information on Widely Used Plant Drugs*. Esher, Surrey, United Kingdom: British Herbal Medicine Association.

Braga, A. S., Abdelbary, M. M. H., Kim, R. R., de Melo, F. P. de S. R., Saldanha, L. L., Dokkedal, A. L., et al. (2022). The Effect of Toothpastes Containing Natural Extracts on Bacterial Species of a Microcosm Biofilm and on Enamel Caries Development. *Antibiotics 2022, Vol. 11, Page 414* 11, 414. doi: 10.3390/ANTIBIOTICS11030414

Braga, A. S., De Melo, F. P. D. S. R., Saldanha, L. L., Dokkedal, A. L., Meissner, T., Bemmann, M., et al. (2021). The Effect of Solutions Containing Extracts of Vochysia tucanorum Mart., Myrcia bellaCambess., Matricaria chamomilla L. and Malva sylvestris L. on Cariogenic Bacterial Species and Enamel Caries Development. *Caries Res* 55, 193–204. doi: 10.1159/000515234

Brezoiu, A. M., Prundeanu, M., Berger, D., Deaconu, M., Matei, C., Oprea, O., et al. (2020). Properties of Salvia officinalis L. and Thymus serpyllum L. Extracts Free and Embedded into Mesopores of Silica and Titania Nanomaterials. *Nanomaterials 2020, Vol. 10, Page 820* 10, 820. doi: 10.3390/NANO10050820

Buckle, J. (1999). Use of aromatherapy as a complementary treatment for chronic pain. *Altern Ther Health Med* 5, 42–51.

Buzigi, E., Pillay, K., and Siwela, M. (2020). Child acceptability of a novel provitamin A carotenoid, iron and zinc-rich complementary food blend prepared from pumpkin and common bean in Uganda: a randomised control trial. *BMC Pediatr* 20, 412. doi: 10.1186/s12887-020-02326-z

Buzzi, M., de Freitas, F., and Winter, M. (2016). A prospective, descriptive study to assess the clinical benefits of using Calendula officinalis hydroglycolic extract for the topical treatment of diabetic foot ulcers. *Ostomy Wound Manage* 62, 8–24.

Buzzonetti, L., Petroni, S., and Federici, M. (2023). Effectiveness of hyaluronic acid and arnica extract ophthalmic solution in reducing dry eye symptoms in pediatric population. *Eur J Ophthalmol* 33, 1011–1017. doi: 10.1177/11206721221128670

Cao Van, H., Guinand, N., Damis, E., Mansbach, A. L., Poncet, A., Hummel, T., et al. (2018). Olfactory stimulation may promote oral feeding in immature newborn: a randomized controlled trial. *European Archives of Oto-Rhino-Laryngology* 275, 125–129. doi: 10.1007/S00405-017-4796-0/METRICS

Carl, W., and Emrich, L. S. (1991). Management of oral mucositis during local radiation and systemic chemotherapy: a study of 98 patients. *J Prosthet Dent* 66, 361–369. doi: 10.1016/0022-3913(91)90264-W

Castro, D. V., Pantoja, A., and Gomajoa, H. A. (2017). Evaluación <i>in vitro</i> de la capacidad antimicrobiana del aceite esencial de eneldo -<i>Anethum graveolens</i>- como inhibidor del crecimiento de <i>Staphylococcus aureus</i>, coliformes y hongos presentes en la carne de trucha. *Revista de la Facultad de Medicina Veterinaria y de Zootecnia* 64, 44–51. doi: 10.15446/rfmvz.v64n2.67212

Cemek, M., Kağa, S., Şimşek, N., Büyükokuroğlu, M. E., and Konuk, M. (2008). Antihyperglycemic and antioxidative potential of Matricaria chamomilla L. in streptozotocin-induced diabetic rats. *J Nat Med* 62, 284–293. doi: 10.1007/S11418-008-0228-1/METRICS

Cemek, M., Yilmaz, E., and Büyükokuroĝlu, M. E. (2010). Protective effect of Matricaria chamomilla on ethanol-induced acute gastric mucosal injury in rats. *http://dx.doi.org/10.3109/13880200903296147* 48, 757–763. doi: 10.3109/13880200903296147

Chaiet, S. R., and Marcus, B. C. (2016). Perioperative Arnica montana for Reduction of Ecchymosis in Rhinoplasty Surgery. *Ann Plast Surg* 76, 477–482. doi: 10.1097/SAP.0000000000000312

Chakraborthy, G. S. (2008). Antimicrobial activity of the leaf extracts of Calendula officinalis (Linn). *Herb Med Toxicol* 2, 65–66.

Chakravartula, S. S. N., Moscetti, R., Farinon, B., Vinciguerra, V., Merendino, N., Bedini, G., et al. (2021). Stinging Nettles as Potential Food Additive: Effect of Drying Processes on Quality Characteristics of Leaf Powders. *Foods 2021, Vol. 10, Page 1152* 10, 1152. doi: 10.3390/FOODS10061152

Chavez, M. L. (2000). With resurgence in use of herbal remedies, unanswered questions take on greater urgency. *J Am Pharm Assoc (Wash)* 40, 349–351. doi: 10.1016/S1086-5802(16)31094-4

Chen, G., Lv, C., Nie, Q., Li, X., Lv, Y., Liao, G., et al. (2024). Essential Oil of Matricaria chamomilla Alleviate Psoriatic-Like Skin Inflammation by Inhibiting PI3K/Akt/mTOR and p38MAPK Signaling Pathway. *Clin Cosmet Investig Dermatol* Volume 17, 59–77. doi: 10.2147/CCID.S445008

Chen, X., Shang, S., Yan, F., Jiang, H., Zhao, G., Tian, S., et al. (2023). Antioxidant Activities of Essential Oils and Their Major Components in Scavenging Free Radicals, Inhibiting Lipid Oxidation and Reducing Cellular Oxidative Stress. *Molecules 2023, Vol. 28, Page 4559* 28, 4559. doi: 10.3390/MOLECULES28114559

COCETTA, V., GIACOMINI, I., TINAZZI, M., BERRETTA, M., QUAGLIARIELLO, V., MAUREA, N., et al. (2023). Maintenance of intestinal epithelial barrier integrity by a combination of probiotics, herbal extract, and vitamins. *Minerva Pediatrics*. doi: 10.23736/S2724-5276.23.07128-8

Colombo, M. L., Marangon, K., and Bugatti, C. (2009). CoulArray electrochemical evaluation of tocopherol and tocotrienol isomers in barley, oat and spelt grains. *Nat Prod Commun* 4, 251–254. doi: 10.1177/1934578x0900400217

Coppock, R. W., and Dziwenka, M. (2021). “St John’s wort (Hypericum perforatum L),” in *Nutraceuticals*, (Elsevier), 661–695. doi: 10.1016/B978-0-12-821038-3.00040-9

Corbo, F., Brunetti, G., Crupi, P., Bortolotti, S., Storlino, G., Piacente, L., et al. (2019). Effects of Sweet Cherry Polyphenols on Enhanced Osteoclastogenesis Associated With Childhood Obesity. *Front Immunol* 10. doi: 10.3389/fimmu.2019.01001

Cortes, A., Garcia-Ferrus, M., Sotillo, J., Guillermo Esteban, J., Toledo, R., and Munoz-Antoli, C. (2017). Effects of dietary intake of garlic on intestinal trematodes. *Parasitol Res* 116, 2119–2129. doi: 10.1007/s00436-017-5511-1

Crowe, S., and Lyons, B. (2004a). Herbal medicine use by children presenting for ambulatory anesthesia and surgery. *Pediatric Anesthesia* 14, 916–919. doi: 10.1111/j.1460-9592.2004.01353.x

Crowe, S., and Lyons, B. (2004b). Herbal medicine use by children presenting for ambulatory anesthesia and surgery. *Pediatric Anesthesia* 14, 916–919. doi: 10.1111/j.1460-9592.2004.01353.x

Czarnecki, M. L., Michlig, J. R., Norton, A. M., Stelter, A. J., and Hainsworth, K. R. (2022). Use of Aromatherapy for Pediatric Surgical Patients. *Pain Manag Nurs* 23, 703–710. doi: 10.1016/J.PMN.2022.08.003

Dağlıoğlu, Y., Öztürk, B. Y., and Khatami, M. (2023). Apoptotic, cytotoxic, antioxidant, and antibacterial activities of biosynthesized silver nanoparticles from nettle leaf. *Microsc Res Tech* 86, 669–685. doi: 10.1002/JEMT.24306

Danby, S. G., AlEnezi, T., Sultan, A., Lavender, T., Chittock, J., Brown, K., et al. (2013). Effect of Olive and Sunflower Seed Oil on the Adult Skin Barrier: Implications for Neonatal Skin Care. *Pediatr Dermatol* 30, 42–50. doi: 10.1111/j.1525-1470.2012.01865.x

Darmstadt, G. L., Badrawi, N., Law, P. A., Ahmed, S., Bashir, M., Iskander, I., et al. (2004). Topically Applied Sunflower Seed Oil Prevents Invasive Bacterial Infections in Preterm Infants in Egypt. *Pediatr Infect Dis J* 23, 719–725. doi: 10.1097/01.inf.0000133047.50836.6f

Davidović-Plavšić, B., Kukavica, B., Škondrić, S., Jimenez-Gallardo, C., and Žabić, M. (2021). Wild garlic extract reduces lipid peroxidation in terbuthylazine-treated human erythrocytes. *Biomarkers* 26, 617–624. doi: 10.1080/1354750X.2021.1953598

Davis, L. E., Shen, J. K., and Cai, Y. (1990). Antifungal activity in human cerebrospinal fluid and plasma after intravenous administration of Allium sativum. *Antimicrob Agents Chemother* 34, 651–653. doi: 10.1128/AAC.34.4.651

Deliorman Orhan, D., Hartevioğlu, A., Küpeli, E., and Yesilada, E. (2007). In vivo anti-inflammatory and antinociceptive activity of the crude extract and fractions from Rosa canina L. fruits. *J Ethnopharmacol* 112, 394–400. doi: 10.1016/J.JEP.2007.03.029

Dhiraj, R. S., and Anjna, K. D. (2013). Studies on traditional herbal pediatrics practices in Jaisinghpur, district Kangra (Himachal Pradesh, India). *Global Journal of Research on Medicinal Plants & Indigenous Medicine* 2, 219.

Dorsch, W., Adelmann-Grill, B., Bayer, T., Ettl, M., Hein, G., Jaggy, H., et al. (1987). Zwiebelextrakte als Asthma-Therapeutika. *Allergologie* 10, 316–324.

[Double-blind comparison of an apple pectin-chamomile extract preparation with placebo in children with diarrhea] - PubMed (n.d.). Available at: https://pubmed.ncbi.nlm.nih.gov/9463302/ (Accessed July 12, 2023).

Duan, X., Li, J., Cui, J., Li, H., Hasan, B., and Xin, X. (2022). Chemical component and in vitro protective effects of Matricaria chamomilla (L.) against lipopolysaccharide insult. *J Ethnopharmacol* 296, 115471. doi: 10.1016/J.JEP.2022.115471

Duarte, C.-M.-E., Quirino, M.-R.-S., Patrocínio, M.-C., Anbinder, A.-L., Engenheiro, R., and Longo, F. J. (2011). Med Oral Patol Oral Cir Bucal. 16, 716–737. doi: 10.4317/medoral.17029

Duran, V., Matic, M., Jovanovć, M., Mimica, N., Gajinov, Z., Poljacki, M., et al. (2005). Results of the clinical examination of an ointment with marigold (Calendula officinalis) extract in the treatment of venous leg ulcers. *Int J Tissue React* 27, 101–6.

Dutto, M., Montù, D., and Raineri, G. (2012). [Enterobiasis in pediatric subjects in north-western Italy: a study of home remedies]. *Ann Ig* 24, 81–84.

Ebrahimi, H., Mardani, A., Basirinezhad, M. H., Hamidzadeh, A., and Eskandari, F. (2022). The effects of Lavender and Chamomile essential oil inhalation aromatherapy on depression, anxiety and stress in older community-dwelling people: A randomized controlled trial. *EXPLORE* 18, 272–278. doi: 10.1016/J.EXPLORE.2020.12.012

Egea, I., Sánchez-Bel, P., Romojaro, F., and Pretel, M. T. (2010). Six edible wild fruits as potential antioxidant additives or nutritional supplements. *Plant Foods Hum Nutr* 65, 121–129. doi: 10.1007/S11130-010-0159-3

Eid, A. M., and Jaradat, N. (2020). Public Knowledge, Attitude, and Practice on Herbal Remedies Used During Pregnancy and Lactation in West Bank Palestine. *Front Pharmacol* 11, 490472. doi: 10.3389/FPHAR.2020.00046/BIBTEX

Ejderhamn, J., Hedenborg, G., and Strandvik, B. (2009). Long-term double-blind study on the influence of dietary fibres on faecal bile acid excretion in juvenile ulcerative colitis. *https://doi.org/10.3109/00365519209115515* 52, 697–706. doi: 10.3109/00365519209115515

El-Hashim, A. Z., Khajah, M. A., Orabi, K. Y., Balakrishnan, S., Sary, H. G., and Abdelali, A. A. (2020). Onion Bulb Extract Downregulates EGFR/ERK1/2/AKT Signaling Pathway and Synergizes With Steroids to Inhibit Allergic Inflammation. *Front Pharmacol* 11, 551683. doi: 10.3389/fphar.2020.551683

Erda, R., Badar, M., Yunaspi, D., and Oktavina, Y. (2021). Compress Onions (Allium Cepa) As A Nonpharmacological Therapy in Febris Toddlers. *Jurnal Aisyah : Jurnal Ilmu Kesehatan* 6, 409–414.

Erol, A., and Hamit, C. kun (2015). Evaluation of the anthelmintic activity of pumpkin seeds (Cucurbita maxima) in mice naturally infected with Aspiculuris tetraptera. *Journal of Pharmacognosy and Phytotherapy* 7, 189–193. doi: 10.5897/JPP2015.0341

Faghihi, T., Haghgoo, R., Taghizade, F., Zareiyan, M., Mehran, M., and Ansari, G. (2021). The clinical and radiographic evaluation of Allium sativum oil (garlic oil) in comparison with mineral trioxide aggregate in primary molar pulpotomy. *Dent Res J (Isfahan)* 18, 100. doi: 10.4103/1735-3327.330881

Fallahi, S., Rostami, A., Delfan, B., Pournia, Y., and Rashidipour, M. (2016). Effect of olive leaf, Satureja khuzestanica, and Allium sativum extracts on Giardia lamblia cysts compared with metronidazole in vitro. *J Parasit Dis* 40, 1204–1209. doi: 10.1007/s12639-015-0650-8

Farouk Abolwafa, N., Mohamed Sayed Osman, Y., Waly El Din Ali, M., and Samir Ahmed El-husseiny, H. (2023). Impact of thyme drink with Honey on Nocturnal Cough and Sleep Quality for Children. *Egyptian Journal of Health Care* 14, 947–957. doi: 10.21608/ejhc.2023.325081

Fazal, H., Akram, M., Ahmad, N., Qaisar, M., Kanwal, F., Rehman, G., et al. (2023). Nutritionally rich biochemical profile in essential oil of various Mentha species and their antimicrobial activities. *Protoplasma* 260, 557–570. doi: 10.1007/S00709-022-01799-2/METRICS

Feitosa, T. F., Vilela, V. L. R., Athayde, A. C. R., Braga, F. R., Dantas, E. S., Vieira, V. D., et al. (2012). Anthelmintic efficacy of pumpkin seed (Cucurbita pepo Linnaeus, 1753) on ostrich gastrointestinal nematodes in a semiarid region of Paraíba State, Brazil. *Trop Anim Health Prod* 45, 123–127. doi: 10.1007/s11250-012-0182-5

Ferrando, J. (1986). [Clinical trial of a topical preparation containing urea, sunflower oil, evening primrose oil, wheat germ oil and sodium pyruvate, in several hyperkeratotic skin conditions]. *Med Cutan Ibero Lat Am* 14, 133–137. Available at: https://europepmc.org/article/MED/3528709 (Accessed June 9, 2023).

Fetni, S., Bertella, N., and Ouahab, A. (2020). LC–DAD/ESI–MS/MS characterization of phenolic constituents in Rosa canina L. and its protective effect in cells. *Biomedical Chromatography* 34, e4961. doi: 10.1002/BMC.4961

Fischer, N., Darmstadt, G. L., Shahunja, K., Crowther, J. M., Kendall, L., Gibson, R. A., et al. (2021). Topical emollient therapy with sunflower seed oil alters the skin microbiota of young children with severe acute malnutrition in Bangladesh: A randomised, controlled study. *J Glob Health* 11, 04047. doi: 10.7189/jogh.11.04047

Franco, E. P. D. de, Contesini, F. J., Lima da Silva, B., Alves de Piloto Fernandes, A. M., Wielewski Leme, C., Gonçalves Cirino, J. P., et al. (2019). Enzyme-assisted modification of flavonoids from Matricaria chamomilla: antioxidant activity and inhibitory effect on digestive enzymes. *https://doi.org/10.1080/14756366.2019.1681989* 35, 42–49. doi: 10.1080/14756366.2019.1681989

Frezza, C., De Vita, D., Spinaci, G., Sarandrea, M., Venditti, A., and Bianco, A. (2019). Secondary metabolites of Tilia tomentosa Moench inflorescences collected in Central Italy: chemotaxonomy relevance and phytochemical rationale of traditional use. *https://doi.org/10.1080/14786419.2018.1550487* 34, 1167–1174. doi: 10.1080/14786419.2018.1550487

Fronza, M., Heinzmann, B., Hamburger, M., Laufer, S., and Merfort, I. (2009). Determination of the wound healing effect of Calendula extracts using the scratch assay with 3T3 fibroblasts. *J Ethnopharmacol* 126, 463–467. doi: 10.1016/j.jep.2009.09.014

Fryatt, J., and Bell, P. (2020). Effect of Peppermint Oil On Postoperative Urinary Retention. *J Pediatr Nurs* 51, 116–118. doi: 10.1016/j.pedn.2020.01.001

Funel, N., Dini, V., Janowska, A., Loggini, B., Minale, M., Grieco, F., et al. (2020). Triticum vulgare extract modulates protein-kinase B and matrix metalloproteinases 9 protein expression in BV-2 cells: Bioactivity on inflammatory pathway associated with molecular mechanism wound healing. *Mediators Inflamm*.

Galdiero, E., Di Onofrio, V., Maione, A., Gambino, E., Gesuele, R., Menale, B., et al. (2020). Allium ursinum and Allium oschaninii against Klebsiella pneumoniae and Candida albicans Mono- and Polymicrobic Biofilms in In Vitro Static and Dynamic Models. *Microorganisms* 8, 336. doi: 10.3390/microorganisms8030336

Gao, L., Li, H., Li, B., Shao, H., Yu, X., Miao, Z., et al. (2022). Traditional uses, phytochemistry, transformation of ingredients and pharmacology of the dried seeds of Raphanus sativus L. (Raphani Semen), A comprehensive review. *J Ethnopharmacol* 294, 115387. doi: 10.1016/J.JEP.2022.115387

Gao, M., Singh, A., Macri, K., Reynolds, C., Singhal, V., Biswal, S., et al. (2011). Antioxidant components of naturally-occurring oils exhibit marked anti-inflammatory activity in epithelial cells of the human upper respiratory system. *Respir Res* 12, 1–15. doi: 10.1186/1465-9921-12-92/TABLES/3

Garami, M., Schuler, D., Babosa, M., Borgulya, G., Hauser, P., Müller, J., et al. (2004). Fermented Wheat Germ Extract Reduces Chemotherapy-Induced Febrile Neutropenia in Pediatric Cancer Patients. *J Pediatr Hematol Oncol* 26, 631–635. doi: 10.1097/01.mph.0000141897.04996.21

Geetha, S., Singh, V., Ram, M. S., Ilavazhagan, G., Banerjee, P. K., and Sawhney, R. C. (2005). Immunomodulatory effects of seabuckthorn (Hippophae rhamnoides L.) against chromium (VI) induced immunosuppression. *Mol Cell Biochem* 278, 101–109. doi: 10.1007/s11010-005-7095-9

Gentil, L. B., Robles, A. C. C., and Grosseman, S. (2010). Uso de terapias complementares por mães em seus filhos: estudo em um hospital universitário. *Cien Saude Colet* 15, 1293–1299. doi: 10.1590/S1413-81232010000700038

Georgiev, Y. N., Paulsen, B. S., Kiyohara, H., Ciz, M., Ognyanov, M. H., Vasicek, O., et al. (2017). Tilia tomentosa pectins exhibit dual mode of action on phagocytes as β-glucuronic acid monomers are abundant in their rhamnogalacturonans I. *Carbohydr Polym* 175, 178–191. doi: 10.1016/J.CARBPOL.2017.07.073

Gerenčer, M., Turecek, P. L., Kistner, O., Mitterer, A., Savidis-Dacho, H., and Barrett, N. P. (2006). In vitro and in vivo anti-retroviral activity of the substance purified from the aqueous extract of Chelidonium majus L. *Antiviral Res* 72, 153–156. doi: 10.1016/j.antiviral.2006.03.008

Getaneh, A., Derseh, F., Abreha, M., and Yirtaw, T. (2018). Misconceptions and traditional practices towards infant teething symptoms among mothers in Southwest Ethiopia. *BMC Oral Health* 18, 159. doi: 10.1186/s12903-018-0619-y

Ghorani, V., Marefati, N., Shakeri, F., Rezaee, R., Boskabady, M., and Boskabady, M. H. (2018). The Effects of Allium Cepa Extract on Tracheal Responsiveness, Lung Inflammatory Cells and Phospholipase A2 Level in Asthmatic Rats. *Iran J Allergy Asthma Immunol* 17, 221–231.

Ghosian Moghaddam, M., Roghani, M., and Maleki, M. (2016). Effect of Hypericum perforatum aqueous extracts on serum lipids, aminotransferases, and lipid peroxidation in hyperlipidemic rats. *Res Cardiovasc Med* 5, 5. doi: 10.5812/cardiovascmed.31326

Glowania, H. J., Raulin, C., and Swoboda, M. (1987). [Effect of chamomile on wound healing--a clinical double-blind study]. *Z Hautkr* 62, 1262, 1267–71.

Göbel, H., Heinze, A., Heinze-Kuhn, K., Göbel, A., and Göbel, C. (2016). Oleum menthae piperitae (Pfefferminzöl) in der Akuttherapie des Kopfschmerzes vom Spannungstyp. *Schmerz* 30, 295–310. doi: 10.1007/S00482-016-0109-6/METRICS

Goes, P., Dutra, C. S., Lisboa, M. R. P., Gondim, D. V., Leitão, R., Brito, G. A. C., et al. (2016). Clinical efficacy of a 1% Matricaria chamomile L. mouthwash and 0.12% chlorhexidine for gingivitis control in patients undergoing orthodontic treatment with fixed appliances. *J Oral Sci* 58, 569–574. doi: 10.2334/JOSNUSD.16-0280

Golsorkhi, H., Qorbani, M., Kamalinejad, M., Sabbaghzadegan, S., Bahrami, M., Vafaee-Shahi, M., et al. (2022). The effect of Rosa canina L. and a polyherbal formulation syrup in patients with attention-deficit/hyperactivity disorder: a study protocol for a multicenter randomized controlled trial. *Trials* 23, 434. doi: 10.1186/s13063-022-06297-7

Gonçalves, A. C., Costa, A. R., Flores-Félix, J. D., Falcão, A., Alves, G., and Silva, L. R. (2022). Anti-Inflammatory and Antiproliferative Properties of Sweet Cherry Phenolic-Rich Extracts. *Molecules* 27, 268. doi: 10.3390/molecules27010268

Grzybek, M., Kukula-Koch, W., Strachecka, A., Jaworska, A., Phiri, A., Paleolog, J., et al. (2016). Evaluation of Anthelmintic Activity and Composition of Pumpkin (Cucurbita pepo L.) Seed Extracts—In Vitro and in Vivo Studies. *Int J Mol Sci* 17, 1456. doi: 10.3390/ijms17091456

Gulbagca, F., Ozdemir, S., Gulcan, M., and Sen, F. (2019). Synthesis and characterization of Rosa canina-mediated biogenic silver nanoparticles for anti-oxidant, antibacterial, antifungal, and DNA cleavage activities. *Heliyon* 5. doi: 10.1016/J.HELIYON.2019.E02980

Gulec, M., Tan, N., Canverdi, O., and Tan, E. (2018). The usage of the most frequently preferred herbal products in Turkey by mothers for themselves and their children. *Istanbul Journal of Pharmacy*. doi: 10.5152/IstanbulJPharm.2017.0014

Gurmu, A. E., Kisi, T., Shibru, H., Graz, B., and Willcox, M. (2018). Treatments used for malaria in young Ethiopian children: a retrospective study. *Malar J* 17, 451. doi: 10.1186/s12936-018-2605-x

Gürol, A., Şener Taplak, A., and Polat, S. (2019a). Herbal supplement products used by mothers to cope with the common health problems in childhood. *Complement Ther Med* 47, 102214. doi: 10.1016/J.CTIM.2019.102214

Gürol, A., Şener Taplak, A., and Polat, S. (2019b). Herbal supplement products used by mothers to cope with the common health problems in childhood. *Complement Ther Med* 47, 102214. doi: 10.1016/j.ctim.2019.102214

Gutman, G., Nunez, A. T., and Fisher, M. (2022). Dysmenorrhea in adolescents. *Curr Probl Pediatr Adolesc Health Care* 52, 101186. doi: 10.1016/J.CPPEDS.2022.101186

Hahm, K. M., Park, S. H., Oh, S. W., Kim, J. H., Yeom, H. S., Lee, H. J., et al. (2021). Aspergillus oryzae-Fermented Wheat Peptone Enhances the Potential of Proliferation and Hydration of Human Keratinocytes through Activation of p44/42 MAPK. *Molecules 2021, Vol. 26, Page 6074* 26, 6074. doi: 10.3390/MOLECULES26196074

Hajhashemi, M., Ghanbari, Z., Movahedi, M., Rafieian, M., Keivani, A., and Haghollahi, F. (2018). The effect of *Achillea millefolium* and *Hypericum perforatum* ointments on episiotomy wound healing in primiparous women. *The Journal of Maternal-Fetal & Neonatal Medicine* 31, 63–69. doi: 10.1080/14767058.2016.1275549

Halsted, C. (2004). The ABC Clinical Guide to Herbsedited by Mark Blumenthal, 2003, 480 pages, hardcover, $69.95. American Botanical Council, Austin, TX. *Am J Clin Nutr* 79, 1127–1128. doi: 10.1093/AJCN/79.6.1127

Hammer, K. D. P., Yum, M.-Y., Dixon, P. M., and Birt, D. F. (2010). Identification of JAK–STAT pathways as important for the anti-inflammatory activity of a Hypericum perforatum fraction and bioactive constituents in RAW 264.7 mouse macrophages. *Phytochemistry* 71, 716–725. doi: 10.1016/j.phytochem.2010.02.006

Hanlon, P. R., Webber, D. M., and Barnes, D. M. (2007). Aqueous extract from Spanish black radish (Raphanus sativus L. Var. niger) induces detoxification enzymes in the HepG2 human hepatoma cell line. *J Agric Food Chem* 55, 6439–6446. doi: 10.1021/JF070530F

Harati, E., Bahrami, M., Razavi, A., Kamalinejad, M., Mohammadian, M., Rastegar, T., et al. (2018). Effects of Viola tricolor Flower Hydroethanolic Extract on Lung Inflammation in a Mouse Model of Chronic Asthma. *Iran J Allergy Asthma Immunol* 17, 409–417.

Heidarifar, R., Mehran, N., Heidari, A., Tehran, H. A., Koohbor, M., and Mansourabad, M. K. (2014). Effect of Dill (Anethum graveolens) on the severity of primary dysmenorrhea in compared with mefenamic acid: A randomized, double-blind trial. *J Res Med Sci* 19, 326–30.

Hejna, M., Kovanda, L., Rossi, L., and Liu, Y. (2021). Mint Oils: In Vitro Ability to Perform Anti-Inflammatory, Antioxidant, and Antimicrobial Activities and to Enhance Intestinal Barrier Integrity. *Antioxidants 2021, Vol. 10, Page 1004* 10, 1004. doi: 10.3390/ANTIOX10071004

Hekmatzadeh, S. F., Bazarganipour, F., Malekzadeh, J., Goodarzi, F., and Aramesh, S. (2014). A randomized clinical trial of the efficacy of applying a simple protocol of boiled Anethum Graveolens seeds on pain intensity and duration of labor stages. *Complement Ther Med* 22, 970–976. doi: 10.1016/j.ctim.2014.10.007

Hellinger, R., Koehbach, J., Fedchuk, H., Sauer, B., Huber, R., Gruber, C. W., et al. (2014). Immunosuppressive activity of an aqueous Viola tricolor herbal extract. *J Ethnopharmacol* 151, 299–306. doi: 10.1016/J.JEP.2013.10.044

Hendrich, A. B., Strugała, P., Dudra, A., Kucharska, A. Z., Sokół-ŁĘtowska, A., Wojnicz, D., et al. (2020). Microbiological, antioxidant and lipoxygenase-1 inhibitory activities of fruit extracts of chosen Rosaceae family species. *Adv Clin Exp Med* 29, 215–224. doi: 10.17219/ACEM/115086

Herasymova, I., Yuryeva, G., Konovalenko, I., and Joumblat, A. (2022). Development of the composition of expectorant syrup for pediatric practice. *Annals of Mechnikov's Institute*, 15–19.

Hills, J. M., and Aaronson, P. I. (1991). The Mechanism of Action of Peppermint Oil on Gastrointestinal Smooth Muscle An Analysis Using Patch Clamp Electrophysiology and Isolated Tissue Pharmacology in Rabbit and Guinea Pig. *Gastroenterology* 101, 55–65.

Hooman, N., Mojab, F., Nickavar, B., and Pouryousefi-Kermani, P. (2009). Diuretic effect of powdered Cerasus avium (cherry) tails on healthy volunteers. *Pak J Pharm Sci* 22, 381–383.

Hritcu, L., and Cioanca, O. (2016). "Prevalence of Use of Herbal Medicines and Complementary and Alternative Medicine in Europe," in *Herbal Medicine in Depression*, (Cham: Springer International Publishing), 135–181. doi: 10.1007/978-3-319-14021-6_5

Hsu, H., Sheth, C. C., and Veses, V. (2020). Herbal Extracts with Antifungal Activity against Candida albicans: A Systematic Review. *Mini-Reviews in Medicinal Chemistry* 21, 90–117. doi: 10.2174/1389557520666200628032116

Huang, N., Rizshsky, L., Hauck, C., Nikolau, B. J., Murphy, P. A., and Birt, D. F. (2011). Identification of anti-inflammatory constituents in Hypericum perforatum and Hypericum gentianoides extracts using RAW 264.7 mouse macrophages. *Phytochemistry* 72, 2015–2023. doi: 10.1016/j.phytochem.2011.07.016

Hübner, W.-D., and Kirste, T. (2001). Experience with St John's Wort (*Hypericum perforatum*) in children under 12 years with symptoms of depression and psychovegetative disturbances. *Phytotherapy Research* 15, 367–370. doi: 10.1002/ptr.829

Hussain, A. I., Anwar, F., Chatha, S. A. S., Latif, S., Sherazi, S. T. H., Ahmad, A., et al. (2013). Chemical composition and bioactivity studies of the essential oils from two Thymus species from the Pakistani flora. *LWT - Food Science and Technology* 50, 185–192. doi: 10.1016/J.LWT.2012.06.003

Hussain, A. I., Anwar, F., Nigam, P. S., Ashraf, M., and Gilani, A. H. (2010). Seasonal variation in content, chemical composition and antimicrobial and cytotoxic activities of essential oils from four Mentha species. *J Sci Food Agric* 90, 1827–1836. doi: 10.1002/JSFA.4021

Hussein, S. N., and Shukur, M. S. (2020). In-vitro anthelmentic efficacy of pumpkin seed oil (Cucurbita pepo) on toxocariosis (Toxocara cati). *Exploratory Animal & Medical Research* 10.

Iannitti, T., Morales-Medina, J. C., Bellavite, P., Rottigni, V., and Palmieri, B. (2016). Effectiveness and Safety of Arnica montana in Post-Surgical Setting, Pain and Inflammation. *Am J Ther* 23, e184–e197. doi: 10.1097/MJT.0000000000000036

Iqbal, Z., Nadeem, Q. K., Khan, M. N., Akhtar, M. S., and Waraich, F. N. (2001). In vitro anthelmintic activity of Allium sativum, Zingiber officinale, Curcurbita mexicana and Ficus religiosa. *Int J Agric Biol* 3, 454–457.

Jafarirad, S., Mehrabi, M., Divband, B., and Kosari-Nasab, M. (2016). Biofabrication of zinc oxide nanoparticles using fruit extract of Rosa canina and their toxic potential against bacteria: A mechanistic approach. *Materials Science and Engineering: C* 59, 296–302. doi: 10.1016/J.MSEC.2015.09.089

Jaiswal, V., and Lee, H. J. (2022). Antioxidant Activity of Urtica dioica: An Important Property Contributing to Multiple Biological Activities. *Antioxidants 2022, Vol. 11, Page 2494* 11, 2494. doi: 10.3390/ANTIOX11122494

Jarić, S., Popović, Z., Mačukanović-Jocić, M., Djurdjević, L., Mijatović, M., Karadžić, B., et al. (2007a). An ethnobotanical study on the usage of wild medicinal herbs from Kopaonik Mountain (Central Serbia). *J Ethnopharmacol* 111, 160–175. doi: 10.1016/j.jep.2006.11.007

Jarić, S., Popović, Z., Mačukanović-Jocić, M., Djurdjević, L., Mijatović, M., Karadžić, B., et al. (2007b). An ethnobotanical study on the usage of wild medicinal herbs from Kopaonik Mountain (Central Serbia). *J Ethnopharmacol* 111, 160–175. doi: 10.1016/J.JEP.2006.11.007

Jarrahi, M., Vafaei, A. A., Taherian, A. A., Miladi, H., and Rashidi Pour, A. (2010). Evaluation of topical Matricaria chamomilla extract activity on linear incisional wound healing in albino rats. *Nat Prod Res* 24, 697–702. doi: 10.1080/14786410701654875

Jeon, H., Yang, D., Lee, N. H., Ahn, M., and Kim, G. (2020). Inhibitory Effect of Black Radish (Raphanus sativus L. var. niger) Extracts on Lipopolysaccharide-Induced Inflammatory Response in the Mouse Monocyte/Macrophage-Like Cell Line RAW 264.7. *Prev Nutr Food Sci* 25, 408–421. doi: 10.3746/PNF.2020.25.4.408

Jia, S., Mustoe, T., and Ketzis, J. (2006). In Vivo Testing of the Wound-healing Activity of a Natural-based Skin Cream for Dogs and Cats. *Planta Med* 72. doi: 10.1055/s-2006-950005

Jovanović, A. A., Ćujić, D., Stojadinović, B., Čutović, N., Živković, J., and Šavikin, K. (2022). Liposomal Bilayer as a Carrier of Rosa canina L. Seed Oil: Physicochemical Characterization, Stability, and Biological Potential. *Molecules* 28. doi: 10.3390/MOLECULES28010276

Juárez-Vázquez, M. D. C., Carranza-Álvarez, C., Alonso-Castro, A. J., González-Alcaraz, V. F., Bravo-Acevedo, E., Chamarro-Tinajero, F. J., et al. (2013). Ethnobotany of medicinal plants used in Xalpatlahuac, Guerrero, México. *J Ethnopharmacol* 148, 521–527. doi: 10.1016/J.JEP.2013.04.048

Jurcău, R., and Jurcău, I. (2013). The effectiveness of Arnica Montana treatment, in sports post-trauma ankle sprains. *Palestrica of the Third Millennium Civilization & Sport* 14.

Kafa, A. H. T., Aslan, R., Celik, C., and Hasbek, M. (2022). Antimicrobial synergism and antibiofilm activities of Pelargonium graveolens, Rosemary officinalis, and Mentha piperita essential oils against extreme drug-resistant Acinetobacter baumannii clinical isolates. *Zeitschrift fur Naturforschung - Section C Journal of Biosciences* 77, 95–104. doi: 10.1515/ZNC-2021-0079/DOWNLOADASSET/SUPPL/J_ZNC-2021-0079_SUPPL_002.JPG

Kaplan, B. (1994). Homoeopathy: 2. In pregnancy and for the under-fives. *Prof Care Mother Child* 4, 185–187.

Kaur, G. J., and Arora, D. S. (2008). In vitro antibacterial activity of three plants belonging to the family Umbelliferae. *Int J Antimicrob Agents* 31, 393–395.

Keefe, J. R., Mao, J. J., Soeller, I., Li, Q. S., and Amsterdam, J. D. (2016). Short-term open-label chamomile (Matricaria chamomilla L.) therapy of moderate to severe generalized anxiety disorder. *Phytomedicine* 23, 1699–1705. doi: 10.1016/J.PHYMED.2016.10.013

Khalili, S., Amiri-Farahani, L., Haghani, S., Bordbar, A., Shojaii, A., and Pezaro, S. (2023). The effect of Pimpinella Anisum herbal tea on human milk volume and weight gain in the preterm infant: a randomized controlled clinical trial. *BMC Complement Med Ther* 23, 1–11. doi: 10.1186/S12906-023-03848-6/TABLES/3

Khan, F. A., Bhat, S. A., and Narayan, S. (2017). Wild edible plants as a food Resource: Traditional Knowledge. *University of Agricultural Science and Technology, Research Gate*.

Khan, M. Z., Azad, A. K., Jan, S., Safdar, M., Bibi, S., Majid, A. M. S. A., et al. (2023). An Experimental and Computational Analysis of Plant Compounds from Whole Urtica dioica L. Plant's Essential Oil for Antioxidant and Antibacterial Activities. *Metabolites 2023, Vol. 13, Page 502* 13, 502. doi: 10.3390/METABO13040502

Kharazmi, A., and Winther, K. (1999). Rose hip inhibits chemotaxis and chemiluminescence of human peripheral blood neutrophils in vitro and reduces certain inflammatory parameters in vivo. *Inflammopharmacology* 7, 377–386. doi: 10.1007/S10787-999-0031-Y

KHATRI, R. S. (2013). Care of Newborn and Infant Skin by Ayurveda. *Pharma Innov* 2A.

Khmel’nitskaia, N., Vorob’ev, K., Kliachko, L., Ankhimova, E., and Kosenko, V. (1998). A comparative study of conservative treatment schemes in chronic tonsillitis in children. *Vest. Otorinolaringol.* 4, 39–42.

Khoda Karami, N., Moatta, F. r, and Ghahiri, A. (2008). Comparison of effectiveness of an herbal drug(celery, saffron, Anise) and mephnamic acid capsule On primary dismenorrhea. *Ofoghe Danesh* 14, 11–19.

Kim, J., Ahn, M., Choi, Y., Kang, T., Kim, J., Lee, N. H., et al. (2020). Alpha-Linolenic Acid Alleviates Dextran Sulfate Sodium-Induced Ulcerative Colitis in Mice. *Inflammation* 43, 1876–1883. doi: 10.1007/S10753-020-01260-7/METRICS

Klövekorn, W., Tepe, A., and Danesch, U. (2007). A randomized, double-blind, vehicle-controlled, half-side comparison with a herbal ointment containing Mahonia aquifolium, Viola tricolor and Centella asiatica for the treatment of mild-to-moderate atopic dermatitis. *Int J Clin Pharmacol Ther* 45, 583–591. doi: 10.5414/CPP45583

Knipping, K., Garssen, J., and Vant Land, B. (2012). An evaluation of the inhibitory effects against rotavirus infection of edible plant extracts. *Virol J* 9, 1–8. doi: 10.1186/1743-422X-9-137/FIGURES/4

Kocić-Tanackov, S., Dimić, G., Mojović, L., Gvozdanović-Varga, J., Djukić-Vuković, A., Tomović, V., et al. (2017). Antifungal activity of the onion (Allium cepa L.) essential oil against Aspergillus, Fusarium and Penicillium species isolated from food. *J Food Process Preserv* 41, e13050.

Koike, A., Barreira, J. C. M., Barros, L., Santos-Buelga, C., Villavicencio, A. L. C. H., and Ferreira, I. C. F. R. (2015). Edible flowers of Viola tricolor L. as a new functional food: Antioxidant activity, individual phenolics and effects of gamma and electron-beam irradiation. *Food Chem* 179, 6–14. doi: 10.1016/j.foodchem.2015.01.123

Komes, D., Belščak-Cvitanović, A., Horžić, D., Rusak, G., Likić, S., and Berendika, M. (2011). Phenolic composition and antioxidant properties of some traditionally used medicinal plants affected by the extraction time and hydrolysis. *Phytochem Anal* 22, 172–180. doi: 10.1002/PCA.1264

Koosha Paydary, Sahra Emamzadeh-Fard, Hamid Reza Khorram Khorshid, Koorosh Kamali, SeyedAhmad SeyedAlinaghi, and Minoo Mohraz (2012). Safety and Efficacy of Setarud (IMOD TM) Among People Living with HIV/AIDS: A Review. *Recent Pat Antiinfect Drug Discov* 7, 66–72. doi: 10.2174/157489112799829756

Koretz, R. L., and Rotblatt, M. (2004). Complementary and alternative medicine in gastroenterology: The good, the bad, and the ugly. *Clinical Gastroenterology and Hepatology* 2, 957–967. doi: 10.1016/S1542-3565(04)00461-6

Kovačević, T. K., Major, N., Sivec, M., Horvat, D., Krpan, M., Hruškar, M., et al. (2023). Phenolic Content, Amino Acids, Volatile Compounds, Antioxidant Capacity, and Their Relationship in Wild Garlic (A. ursinum L.). *Foods* 12, 2110. doi: 10.3390/foods12112110

Kozan, E., Küpeli, E., and Yesilada, E. (2006). Evaluation of some plants used in Turkish folk medicine against parasitic infections for their in vivo anthelmintic activity. *J Ethnopharmacol* 108, 211–216. doi: 10.1016/j.jep.2006.05.003

Krishnakumari, M. K., and Majumder, S. K. (1960). Bioassay of piperazine and some plant products with earthworms. *J Sci Ind Res (India)* 19, 202–204.

Křížkovská, B., Hoang, L., Brdová, D., Klementová, K., Szemerédi, N., Loučková, A., et al. (2023). Modulation of the bacterial virulence and resistance by well-known European medicinal herbs. *J Ethnopharmacol* 312, 116484. doi: 10.1016/J.JEP.2023.116484

Krstin, S., Sobeh, M., Braun, M., and Wink, M. (2018). Tulbaghia violacea and Allium ursinum Extracts Exhibit Anti-Parasitic and Antimicrobial Activities. *Molecules* 23, 313. doi: 10.3390/molecules23020313

Krycky, I. O. ;, Hoshchynskyi, P. V. ;, Dzhyvak, V. G. ;, Protsailo, and Badiuk, N. S. (n.d.). APPLICATION OF PHYTOCYLINDERS WITH LIPOPHILIC COMPLEX OF ROSA CANINA AND CHAMOMILE EXTRACT FOR TREATMENT OF CONSTIPATION IN PEDIATRIC SURGERY PRACTICE. *Archives • 2021* • 3, 1103–1106. doi: 10.3748/wjg.v22.i30.6864

Kumarasamy, Y., Cox, P. J., Jaspars, M., Nahar, L., and Sarker, S. D. (2002). Screening seeds of Scottish plants for antibacterial activity. *J Ethnopharmacol* 83, 73–77. doi: 10.1016/S0378-8741(02)00214-3

Lacy, B. E., Pimentel, M., Brenner, D. M., Chey, W. D., Keefer, L. A., Long, M. D., et al. (2021). ACG Clinical Guideline: Management of Irritable Bowel Syndrome. *American Journal of Gastroenterology* 116, 17–44. doi: 10.14309/AJG.0000000000001036

Lattanzio, F., Greco, E., Carretta, D., Cervellati, R., Govoni, P., and Speroni, E. (2011). In vivo anti-inflammatory effect of Rosa canina L. extract. *J Ethnopharmacol* 137, 880–885. doi: 10.1016/J.JEP.2011.07.006

Leahu, A., Damian, C., Oroian, M., Juravle, L., and Ropciuc, S. (2015). Physico-chemical and Antioxidant Properties of two Medicinal Wild Plants Grown in Moldova Region. *Scientific Papers: Animal Science & Biotechnologies/Lucrari Stiintifice: Zootehnie si Biotehnologii* 48.

Leahu, A., Hretcanu, C. E., Roşu, A. I., and Ghinea, C. (2019). Traditional uses of wild berries in the Bukovina region (Romania). *Food and Environment Safety* 18, 279–286.

Lee, J. H., Ki, H. H., Kim, D. K., and Lee, Y. M. (2018). Triticum aestivum sprout extract attenuates 2,4-dinitrochlorobenzene-induced atopic dermatitis-like skin lesions in mice and the expression of chemokines in human keratinocytes. *Mol Med Rep* 18, 3461–3468. doi: 10.3892/MMR.2018.9339/HTML

Lee, J. H., Lim, J. Y., Jeon, Y. D., Yun, D. H., Lee, Y. M., and Kim, D. K. (2023). Extract of Wheatgrass and Aronia Mixture Ameliorates Atopic Dermatitis-Related Symptoms by Suppressing Inflammatory Response and Oxidative Stress In Vitro and In Vivo. *Antioxidants* 12, 27. doi: 10.3390/ANTIOX12010027/S1

Lee, J.-H., Kim, D.-K., Lee, W.-M., Lee, S.-J., and Hong, S.-H. . (2007a). The Effects of Arnica Montana Gel for the Decreasing on the Swelling and Bruise. *Journal of the Korean Society of Aesthetic Plastic Surgery* , 151–158.

Lee, Y.-C., Kim, S.-H., Roh, S.-S., Choi, H.-Y., and Seo, Y.-B. (2007b). Suppressive effects of Chelidonium majus methanol extract in knee joint, regional lymph nodes, and spleen on collagen-induced arthritis in mice. *J Ethnopharmacol* 112, 40–48. doi: 10.1016/j.jep.2007.01.033

Leicester, R. J., and Hunt, R. H. (1982). Peppermint oil to reduce colonic spasm during endoscopy. *Lancet* 2, 989. doi: 10.1016/S0140-6736(82)90191-X

Leporatti, M. L., and Corradi, L. (2001). Ethnopharmacobotanical remarks on the Province of Chieti town (Abruzzo, Central Italy). *J Ethnopharmacol* 74, 17–40. doi: 10.1016/S0378-8741(00)00325-1

Leporatti, M. L., and Ghedira, K. (2009). Comparative analysis of medicinal plants used in traditional medicine in Italy and Tunisia. *J Ethnobiol Ethnomed* 5, 31. doi: 10.1186/1746-4269-5-31

Leporatti, M. L., and Ivancheva, S. (2003). Preliminary comparative analysis of medicinal plants used in the traditional medicine of Bulgaria and Italy. *J Ethnopharmacol* 87, 123–142. doi: 10.1016/S0378-8741(03)00047-3

Liu, S., Guo, W., Jia, Y., Ye, B., Liu, S., Fu, S., et al. (2021). Menthol Targeting AMPK Alleviates the Inflammatory Response of Bovine Mammary Epithelial Cells and Restores the Synthesis of Milk Fat and Milk Protein. *Front Immunol* 12, 782989. doi: 10.3389/FIMMU.2021.782989/BIBTEX

Lohse, B., Stotts, J. L., and Priebe, J. R. (2006). Survey of herbal use by Kansas and Wisconsin WIC participants reveals moderate, appropriate use and identifies herbal education needs. *J Am Diet Assoc* 106, 227–237. doi: 10.1016/j.jada.2005.10.033

Luczaj, L., Pieroni, A., Tardío, J., Pardo-de-Santayana, M., Sõukand, R., Svanberg, I., et al. (2012). Wild food plant use in 21 st century Europe, the disapperance of old traditions and the search for new ciusines involving wild edibles. *Acta societatis botanicorum poloniae* 81.

Łuczaj, Ł., and Szymański, W. M. (2007). Wild vascular plants gathered for consumption in the Polish countryside: a review. *J Ethnobiol Ethnomed* 3, 17. doi: 10.1186/1746-4269-3-17

Ludwig, S., Stier, H., and Weykam, S. (2015). Evaluation of Blood Alcohol Concentrations after Oral Administration of a Fixed Combination of Thyme Herb and Primrose Root Fluid Extract to Children with Acute Bronchitis. *Drug Res* 66, 69–73. doi: 10.1055/s-0034-1398543

Lugasi, A., Blázovics, A., Hagymási, K., Kocsis, I., and Kéry, Á. (2005). Antioxidant effect of squeezed juice from black radish (Raphanus sativus L. var niger) in alimentary hyperlipidaemia in rats. *Phytother Res* 19, 587–591. doi: 10.1002/PTR.1655

Lugasi, A., Dworschák, E., Blázovics, A., and Kéry, Á. (1998). Antioxidant and free radical scavenging properties of squeezed juice from black radish (Raphanus sativus l. varniger) root. *Phytotherapy Research* 12, 502–506. doi: 10.1002/(SICI)1099-1573(199811)12:7<502::AID-PTR336>3.0.CO;2-I

Lukačovičová, O., and Havránek, E. (2015). The utilization of radionuclide X-ray spectrometry in the determination of elements in medicinal plants and medicinal products used as antianemics. *Ceska Slov Farm* 64, 220–222. Available at: https://pubmed.ncbi.nlm.nih.gov/28649823/ (Accessed June 8, 2023).

Luo, X., Zhang, H., Duan, Y., and Chen, G. (2018). Protective effects of radish (Raphanus sativus L.) leaves extract against hydrogen peroxide-induced oxidative damage in human fetal lung fibroblast (MRC-5) cells. *Biomedicine & Pharmacotherapy* 103, 406–414. doi: 10.1016/J.BIOPHA.2018.04.049

Macêdo, S. B., Ferreira, L. R., Perazzo, F. F., and Tavares Carvalho, J. C. (2004). Anti-inflammatory activity of Arnica montana 6cH: preclinical study in animals. *Homeopathy* 93, 84–87. doi: 10.1016/j.homp.2004.02.006

Maciej Serda, Becker, F. G., Cleary, M., Team, R. M., Holtermann, H., The, D., et al. (2006). Evaluation of analgesic and anti inflammatory activity of Pimpinella anisum fixed oil extract. *INDIAN VETERINARY JOURNAL* 83, 343–354. doi: 10.2/JQUERY.MIN.JS

Macovei, I., Luca, S. V., Skalicka-Woźniak, K., Horhogea, C. E., Rimbu, C. M., Sacarescu, L., et al. (2023). Silver Nanoparticles Synthesized from Abies alba and Pinus sylvestris Bark Extracts: Characterization, Antioxidant, Cytotoxic, and Antibacterial Effects. *Antioxidants (Basel)* 12. doi: 10.3390/antiox12040797

Magi, G., Marini, E., and Facinelli, B. (2015). Antimicrobial activity of essential oils and carvacrol, and synergy of carvacrol and erythromycin, against clinical, erythromycin-resistant Group A Streptococci. *Front Microbiol* 6. doi: 10.3389/FMICB.2015.00165

Maldonade, I. R., Amaro, G. B., Luengo, R. F. A., Ribeiro, R. L. V., Lozada, M. I. O., Oliveira, L. L., et al. (2020). Phytochemical characterization of pumpkin seed with antiparasitic action. *Acta Hortic*, 127–134. doi: 10.17660/ActaHortic.2020.1287.17

Mann, C., and Staba, E. (1986). The chemistry, pharmacology, and commercial formulations of chamomile. *Herbs, spices and medicinal plants* 1, 235–280.

Manuele, M. G., Ferraro, G., and Anesini, C. (2008). Effect of Tilia x viridis flower extract on the proliferation of a lymphoma cell line and on normal murine lymphocytes: contribution of monoterpenes, especially limonene. *Phytother Res* 22, 1520–1526. doi: 10.1002/PTR.2524

Marefati, N., Eftekhar, N., Kaveh, M., Boskabadi, J., Beheshti, F., and Boskabady, M. H. (2018). The Effect of Allium cepa Extract on Lung Oxidant, Antioxidant, and Immunological Biomarkers in Ovalbumin-Sensitized Rats. *Medical Principles and Practice*. doi: 10.1159/000487885

Martinelli, M., Ummarino, D., Giugliano, F. P., Sciorio, E., Tortora, C., Bruzzese, D., et al. (2017). Efficacy of a standardized extract of Matricariae chamomilla L., Melissa officinalis L. and tyndallized Lactobacillus acidophilus (HA122) in infantile colic: An open randomized controlled trial. *Neurogastroenterol Motil* 29. doi: 10.1111/NMO.13145

Martins, I., Almeida, D., Yoon, I.-S., Costa, E. F., Magalhães, W. V, and Di Stasi, L. C. (2022). Recent Advances in Herbal-Derived Products with Skin Anti-Aging Properties and Cosmetic Applications. *Molecules 2022, Vol. 27, Page 7518* 27, 7518. doi: 10.3390/MOLECULES27217518

Marwaha, R. K., Bansal, D., Kaur, S., and Trehan, A. (2004). Wheat grass juice reduces transfusion requirement in patients with thalassemia major: a pilot study. *Indian Pediatr* 41, 716–720. Available at: https://pubmed.ncbi.nlm.nih.gov/15297687/ (Accessed June 9, 2023).

Masoumi, S., Asl, H., Poorolajal, J., Panah, M., and Oliaei, S. (2016). Evaluation of mint efficacy regarding dysmenorrhea in comparison with mefenamic acid: A double blinded randomized crossover study. *Iran J Nurs Midwifery Res* 21, 363–367. doi: 10.4103/1735-9066.185574

Matejić, J. S., Stefanović, N., Ivković, M., Živanović, N., Marin, P. D., and Džamić, A. M. (2020). Traditional uses of autochthonous medicinal and ritual plants and other remedies for health in Eastern and South-Eastern Serbia. *J Ethnopharmacol* 261. doi: 10.1016/J.JEP.2020.113186

Matricaria chamomilla L. (n.d.). Available at: https://www.worldfloraonline.org/taxon/wfo-0000003146 (Accessed April 6, 2024).

Matsuda, H., Ninomiya, K., Shimoda, H., and Yoshikawa, M. (2002). Hepatoprotective principles from the flowers of Tilia argentea (linden): Structure requirements of tiliroside and mechanisms of action. *Bioorg Med Chem* 10, 707–712. doi: 10.1016/S0968-0896(01)00321-2

McKay, D. L., and Blumberg, J. B. (2006). A review of the bioactivity and potential health benefits of peppermint tea (Mentha piperita L.). *Phytotherapy Research* 20, 619–633. doi: 10.1002/PTR.1936

Meenakshisundaram, A., Harikrishnan, T. J., and Anna, T. (2017). Anthelmintic evaluation of Cucurbita pepo againstgastrointestinal nematodes of sheep. *Indian J Anim Res*. doi: 10.18805/ijar.v0iOF.7264

Mehmood, M. H., Munir, S., Khalid, U. A., Asrar, M., and Gilani, A. H. (2015). Antidiarrhoeal, antisecretory and antispasmodic activities of Matricaria chamomilla are mediated predominantly through K+-channels activation. *BMC Complement Altern Med* 15, 1–9. doi: 10.1186/S12906-015-0595-6/FIGURES/4

Memarzia, A., Amin, F., Saadat, S., Jalali, M., Ghasemi, Z., and Boskabady, M. H. (2019). The contribution of beta-2 adrenergic, muscarinic and histamine (H1) receptors, calcium and potassium channels and cyclooxygenase pathway

in the relaxant effect of Allium cepa L. on the tracheal smooth muscle. *J Ethnopharmacol* 241, 112012. doi: https://doi.org/10.1016/j.jep.2019.112012
Meneses, C., Valdes-Gonzalez, M., Garrido-Suárez, B. B., and Garrido, G. (2023). Systematic review on the anxiolytic and hypnotic effects of flower extracts in in vivo pre-clinical studies published from 2010 to 2020. *Phytotherapy Research* 37, 2144–2167. doi: 10.1002/PTR.7830
Meral, I., and Kanter, M. (2003). Effects of Nigella sativa L. and Urtica dioica L. on Selected Mineral Status and Hematological Values in CCl4-Treated Rats. *Biol Trace Elem Res* 96, 263–270. doi: 10.1385/BTER:96:1-3:263/METRICS
Mihailovic-Stanojevic, N., Belščak-Cvitanović, A., Grujić-Milanović, J., Ivanov, M., Jovović, D., Bugarski, D., et al. (2013). Antioxidant and Antihypertensive Activity of Extract from Thymus serpyllum L. in Experimental Hypertension. *Plant Foods for Human Nutrition* 68, 235–240. doi: 10.1007/S11130-013-0368-7/METRICS
Mihaylova, D., Vrancheva, R., Desseva, I., Ivanov, I., Dincheva, I., Popova, M., et al. (2019). Analysis of the GC-MS of volatile compounds and the phytochemical profile and antioxidant activities of some Bulgarian medicinal plants. *Zeitschrift fur Naturforschung - Section C Journal of Biosciences* 74, 45–54. doi: 10.1515/ZNC-2018-0122/MACHINEREADABLECITATION/RIS
Miraldi, E., Ferri, S., and Mostaghimi, V. (2001). Botanical drugs and preparations in the traditional medicine of West Azerbaijan (Iran). *J Ethnopharmacol* 75, 77–87. doi: 10.1016/S0378-8741(00)00381-0
Mogosan, C., Vostinaru, O., Oprean, R., Heghes, C., Filip, L., Balica, G., et al. (2017). A Comparative Analysis of the Chemical Composition, Anti-Inflammatory, and Antinociceptive Effects of the Essential Oils from Three Species of Mentha Cultivated in Romania. *Molecules* 22. doi: 10.3390/MOLECULES22020263
Mohammad, I. H. (2012). Use of Anethum graveolens in the management of patients with irritable bowel syndrome. *Mustansiriya Medical Journal* 11, 94–98.
Moharreri, M., Vakili, R., Oskoueian, E., and Gh, R. (2022). Evaluation of Microencapsulated Essential Oils in Broilers Challenged with Salmonella Enteritidis: A Focus on the Body’s Antioxidant Status, Gut Microbiology, and Morphology. *Arch Razi Inst* 77, 629–639. doi: 10.22092/ARI.2021.354334.1634
Moldovan, C., Babota, M., Mocan, A., Menghini, L., Cesa, S., Gavan, A., et al. (2021). Optimization of the drying process of autumn fruits rich in antioxidants: a study focusing on rosehip (Rosa canina L.) and sea buckthorn (Elaeagnus rhamnoides (L.) A. Nelson) and their bioactive properties. *Food Funct* 12, 3939–3953. doi: 10.1039/D0FO02783A
Montoya-Arroyo, A., Toro-González, C., Sus, N., Warner, J., Esquivel, P., Jiménez, V. M., et al. (2022). Vitamin E and carotenoid profiles in leaves, stems, petioles and flowers of stinging nettle (Urtica leptophylla Kunth) from Costa Rica. *J Sci Food Agric* 102, 6340–6348. doi: 10.1002/JSFA.11985

Moosavy, M., Shavisi, N., and Khatibi, S. (2018). Therapeutic effects of Pumpkin in Islamic Texts, Islamic Iranian Traditional Medicine and Modern Medicine. *Med Hist J* 9, 77–92.

Muhe, L., Kidane, Y., Shamebo, D., Krantz, I., and Freij, L. (1994). The Butajira Rural Health Project in Ethiopia: mothers' perceptions and practices in the care of children with acute respiratory infections. *Int J Health Sci (Qassim)* 5, 99–103.

Muršić, I. (2020). Treatment of Verruca vulgaris in Traditional Medicine. *Acta Clin Croat*. doi: 10.20471/acc.2020.59.04.22

Musidlak, O., Warowicka, A., Broniarczyk, J., Adamczyk, D., Goździcka-Józefiak, A., and Nawrot, R. (2022). The Activity of Chelidonium majus L. Latex and Its Components on HPV Reveal Insights into the Antiviral Molecular Mechanism. *Int J Mol Sci* 23, 9241. doi: 10.3390/ijms23169241

Mussarat, S., Adnan, M., Begum, S., Alamgeer, Ullah, R., and Kowalczyk, A. (2022). In Vivo Efficacy, Toxicity Assessment, and Elemental Analysis of Traditionally Used Polyherbal Recipe for Diarrhea. *Evidence-based Complementary and Alternative Medicine* 2022. doi: 10.1155/2022/5977795

Mustafa, B., Hajdari, A., Pieroni, A., Pulaj, B., Koro, X., and Quave, C. L. (2015). A cross-cultural comparison of folk plant uses among Albanians, Bosniaks, Gorani and Turks living in south Kosovo. *J Ethnobiol Ethnomed* 11, 1–26. doi: 10.1186/S13002-015-0023-5/FIGURES/6

Nadpal, J. D., Lesjak, M. M., Šibul, F. S., Anačkov, G. T., Četojević-Simin, D. D., Mimica-Dukić, N. M., et al. (2016). Comparative study of biological activities and phytochemical composition of two rose hips and their preserves: Rosa canina L. and Rosa arvensis Huds. *Food Chem* 192, 907–914. doi: 10.1016/J.FOODCHEM.2015.07.089

Nair, R., and CHANDA, S. (2007). Antibacterial activities of some medicinal plants of the western region of India. *Turkish Journal of Biology* 31, 231–236.

Nakamura, A., Kawahara, A., Takahashi, H., Kuda, T., and Kimura, B. (2022). Comparison between the Antimicrobial Activity of Essential Oils and Their Components in the Vapor Phase against Food-related Bacteria. *J Oleo Sci* 71, 411–417. doi: 10.5650/JOS.ESS21337

Nam, H.-H., Nan, L., and Choo, B.-K. (2021). Anti-Inflammation and Protective Effects of Anethum graveolens L. (Dill Seeds) on Esophageal Mucosa Damages in Reflux Esophagitis-Induced Rats. *Foods* 10, 2500. doi: 10.3390/foods10102500

Nane, D., Hatløy, A., and Lindtjørn, B. (2021). A local-ingredients-based supplement is an alternative to corn-soy blends plus for treating moderate acute malnutrition among children aged 6 to 59 months: A randomized controlled non-inferiority trial in Wolaita, Southern Ethiopia. *PLoS One* 16, e0258715. doi: 10.1371/journal.pone.0258715

Nargesi, S., Nargesi, S., Moayeri, A., Ghorbani, A., Seifinejad, Y., Shirzadpour, E., et al. (2018). The effects of Matricaria chamomilla L.hydroalcoholic extract on atherosclerotic plaques, antioxidant activity, lipid profile and inflammatory indicators in rats. *Biomedical Research and Therapy* 5, 2752–2761. doi: 10.15419/bmrat.v5i10.490

Naseri, M., Mojab, F., Khodadoost, M., Kamalinejad, M., Davati, A., Choopani, R., et al. (2012). The Study of Anti-Inflammatory Activity of Oil-Based Dill (Anethum graveolens L.) Extract Used Topically in Formalin-Induced Inflammation Male Rat Paw. *Iran J Pharm Res* 11, 1169–74.

Nathan, M., and Scholten, R. (1999). The Complete German Commission E Monographs: Therapeutic Guide to Herbal Medicines. *Ann Intern Med* 130, 459. doi: 10.7326/0003-4819-130-5-199903020-00024

Nawrot, J., Wilk-Jędrusik, M., Nawrot, S., Nawrot, K., Wilk, B., Dawid-Pać, R., et al. (2020). Milky Sap of Greater Celandine (Chelidonium majus L.) and Anti-Viral Properties. *Int J Environ Res Public Health* 17. doi: 10.3390/ijerph17051540

Nawrot, R., Warowicka, A., Rudzki, P. J., Musidlak, O., Dolata, K. M., Musijowski, J., et al. (2021). Combined Protein and Alkaloid Research of Chelidonium majus Latex Reveals CmMLP1 Accompanied by Alkaloids with Cytotoxic Potential to Human Cervical Carcinoma Cells. *Int J Mol Sci* 22, 11838. doi: 10.3390/ijms222111838

Negele, L., Schneider, B., Ristl, R., Stulnig, T. M., Willfort-Ehringer, A., Helk, O., et al. (2015). Effect of a low-fat diet enriched either with rapeseed oil or sunflower oil on plasma lipoproteins in children and adolescents with familial hypercholesterolaemia. Results of a pilot study. *Eur J Clin Nutr* 69, 337–343. doi: 10.1038/ejcn.2014.234

Ni, W., Gao, T., Wang, H., Du, Y., Li, J., Li, C., et al. (2013). Anti-fatigue activity of polysaccharides from the fruits of four Tibetan plateau indigenous medicinal plants. *J Ethnopharmacol* 150, 529–535. doi: 10.1016/j.jep.2013.08.055

Nicolaus, C., Junghanns, S., Hartmann, A., Murillo, R., Ganzera, M., and Merfort, I. (2017). In vitro studies to evaluate the wound healing properties of Calendula officinalis extracts. *J Ethnopharmacol* 196, 94–103. doi: 10.1016/j.jep.2016.12.006

Nicolescu, A., Babotă, M., Zhang, L., Bunea, C. I., Gavrilaș, L., Vodnar, D. C., et al. (2022). Optimized Ultrasound-Assisted Enzymatic Extraction of Phenolic Compounds from Rosa canina L. Pseudo-Fruits (Rosehip) and Their Biological Activity. *Antioxidants* 11, 1123. doi: 10.3390/ANTIOX11061123/S1

Niederhofer, H. (2009). Observational study: Matricaria chamomilla may improve some symptoms of attention-deficit hyperactivity disorder. *Phytomedicine* 16, 284–286. doi: 10.1016/J.PHYMED.2008.10.006

Nikkhahi, M., Souri, E., Sarkhail, P., Baeeri, M., and Mohammadhosseini, N. (2018). Evaluation of anti-tyrosinase activity of Allium ursinum extracts and their metal complexes. *Acta Sci Pol Technol Aliment* 17, 219–226. doi: 10.17306/J.AFS.0585

Niknam, S., Tofighi, Z., Faramarzi, M. A., Abdollahifar, M. A., Sajadi, E., Dinarvand, R., et al. (2021). Polyherbal combination for wound healing: Matricaria chamomilla L. and Punica granatum L. *DARU, Journal of Pharmaceutical Sciences* 29, 133–145. doi: 10.1007/S40199-021-00392-X/METRICS

Niwa, Y., Tominaga, K., and Yoshida, K. (1998). Successful treatment of severe atopic dermatitis-complicated cataract and male infertility with a natural product antioxidant. *Int J Tissue React* 20, 63–69. Available at: https://europepmc.org/article/MED/9638503 (Accessed June 9, 2023).

N'Jai, A. U., Kemp, M. Q., Metzger, B. T., Hanlon, P. R., Robbins, M., Czuyprynski, C., et al. (2012). Spanish black radish (Raphanus sativus L. Var. niger) diet enhances clearance of DMBA and diminishes toxic effects on bone marrow progenitor cells. *Nutr Cancer* 64, 1038–1048. doi: 10.1080/01635581.2012.714831

Noonan, K., Arensman, R. M., and Hoover, J. D. (2004). Herbal Medication Use in the Pediatric Surgical Patient. *J Pediatr Surg* 39, 500–503. doi: 10.1016/j.jpedsurg.2003.11.017

Nóra Papp, Sámuel Barthaa, Gyöngyvér Boris, and Lajos Balogh (2011). Traditional Uses of Medicinal Plants for Respiratory Diseases in Transylvania. *Nat Prod Commun* 6, 1459–1460.

Nunes, A. R., Flores-Félix, J. D., Gonçalves, A. C., Falcão, A., Alves, G., and Silva, L. R. (2022). Anti-Inflammatory and Antimicrobial Activities of Portuguese Prunus avium L. (Sweet Cherry) By-Products Extracts. *Nutrients* 14, 4576. doi: 10.3390/nu14214576

Okpo, S., and Nnajekwu, O. (2013). <i>In vitro</i> anthelmintic activity of the seed extracts of three plants of the Cucurbitaceae family on <i>Lumbricus terrestris</i> *Journal of Pharmacy & Bioresources* 9. doi: 10.4314/jpb.v9i2.6

Olioso, D., Marzotto, M., Bonafini, C., Brizzi, M., and Bellavite, P. (2016). Arnica montana effects on gene expression in a human macrophage cell line. Evaluation by quantitative Real-Time PCR. *Homeopathy* 105, 131–147. doi: 10.1016/j.homp.2016.02.001

Oliveira, T. T., Campos, K. M., Cerqueira-Lima, A. T., Cana Brasil Carneiro, T., da Silva Velozo, E., Ribeiro Melo, I. C. A., et al. (2015). Potential therapeutic effect of Allium cepa L. and quercetin in a murine model of Blomia tropicalis induced asthma. *Daru* 23, 18. doi: 10.1186/s40199-015-0098-5

Ortiz, M. I., Cariño-Cortés, R., Ponce-Monter, H. A., González-García, M. P., Castañeda-Hernández, G., and Salinas-Caballero, M. (2017). Synergistic Interaction of Matricaria Chamomilla Extract with Diclofenac and Indomethacin on Carrageenan-Induced Paw Inflammation in Rats. *Drug Dev Res* 78, 360–367. doi: 10.1002/DDR.21401

Ortiz, M. I., Fernández-Martínez, E., Soria-Jasso, L. E., Lucas-Gómez, I., Villagómez-Ibarra, R., González-García, M. P., et al. (2016). Isolation, identification and molecular docking as cyclooxygenase (COX) inhibitors of the main constituents of Matricaria chamomilla L. extract and its synergistic interaction with diclofenac on nociception and gastric damage in rats. *Biomedicine & Pharmacotherapy* 78, 248–256. doi: 10.1016/J.BIOPHA.2016.01.029

Özalkaya, E., Aslandoğdu, Z., Özkoral, A., Topcuoğlu, S., and Karatekin, G. (2018). Effect of a galactagogue herbal tea on breast milk production and prolactin secretion by mothers of preterm babies. *Niger J Clin Pract* 21, 38–42. doi: 10.4103/1119-3077.224788

Öztürk, Y., Aydin, S., Başer, K. H. C., Kirimer, N., and Kurtar-Öztürk, N. (1992). Hepatoprotective activity ofHypericum perforatum L. alcoholic extract in rodents. *Phytotherapy Research* 6, 44–46. doi: 10.1002/ptr.2650060111

Panahi, Y., Sharif, M. R., Sharif, A., Beiraghdar, F., Zahiri, Z., Amirchoopani, G., et al. (2012). A randomized comparative trial on the therapeutic efficacy of topical aloe vera and calendula officinalis on diaper dermatitis in children. *The Scientific World Journal* 2012, 810234. doi: 10.1100/2012/810234

Parandin, R., Ghowsi, M., and Dadbod, A. (2023). Protective effects of hydroalcoholic extract of Rosa canina L. fruit on cyclophosphamide-induced testicular toxicity in mice. *Avicenna J Phytomed* 13, 7–17. doi: 10.22038/AJP.2022.20893

Parisio, C., Lucarini, E., Micheli, L., Toti, A., Mannelli, L. D. C., Antonini, G., et al. (2019). Researching New Therapeutic Approaches for Abdominal Visceral Pain Treatment: Preclinical Effects of an Assembled System of Molecules of Vegetal Origin. *Nutrients 2020, Vol. 12, Page 22* 12, 22. doi: 10.3390/NU12010022

Passalacqua, N. G., Guarrera, P. M., and De Fine, G. (2007). Contribution to the knowledge of the folk plant medicine in Calabria region (Southern Italy). *Fitoterapia* 78, 52–68. doi: 10.1016/j.fitote.2006.07.005

Pastare, L., Berga, M., Kienkas, L., Boroduskis, M., Ramata-Stunda, A., Reihmane, D., et al. (2023). Exploring the Potential of Supercritical Fluid Extraction of Matricaria chamomilla White Ray Florets as a Source of Bioactive (Cosmetic) Ingredients. *Antioxidants* 12, 1092. doi: 10.3390/ANTIOX12051092/S1

Patzelt-Wenczler, R., and Ponce-Pöschl, E. (2000). Proof of efficacy of Kamillosan(R) cream in atopic eczema. *Eur J Med Res* 5, 171–5.

Pavlić, B., Mrkonjić, Ž., Teslić, N., Kljakić, A. C., Pojić, M., Mandić, A., et al. (2022). Natural Deep Eutectic Solvent (NADES) Extraction Improves Polyphenol Yield and Antioxidant Activity of Wild Thyme (Thymus serpyllum L.) Extracts. *Molecules 2022, Vol. 27, Page 1508* 27, 1508. doi: 10.3390/MOLECULES27051508

Pavlović, D. R., Veljković, M., Stojanović, N. M., Gočmanac-Ignjatović, M., Mihailov-Krstev, T., Branković, S., et al. (2017). Influence of different wild-garlic (*Allium ursinum*) extracts on the gastrointestinal system: spasmolytic, antimicrobial and antioxidant properties. *Journal of Pharmacy and Pharmacology* 69, 1208–1218. doi: 10.1111/jphp.12746

Peña, F., Valencia, S., Tereucán, G., Nahuelcura, J., Jiménez-Aspee, F., Cornejo, P., et al. (2023). Bioactive Compounds and Antioxidant Activity in the Fruit of Rosehip (Rosa canina L. and Rosa rubiginosa L.). *Molecules* 28, 3544. doi: 10.3390/MOLECULES28083544/S1

Perrone, S., Coppi, S., Coviello, C., Cecchi, S., Becucci, E., Tataranno, M. L., et al. (2012). Efficacy of Arnica Echinacea powder in umbilical cord care in a large cohort study. *The Journal of Maternal-Fetal & Neonatal Medicine* 25, 1111–1113.

Piana, M., Silva, M. A., Trevisan, G., Brum, T. F. de, Silva, C. R., Boligon, A. A., et al. (2013). Antiinflammatory effects of Viola tricolor gel in a model of sunburn in rats and the gel stability study. *J Ethnopharmacol* 150, 458–465. doi: 10.1016/j.jep.2013.08.040

Picon, P. D., Picon, R. V., Costa, A. F., Sander, G. B., Amaral, K. M., Aboy, A. L., et al. (2010). Randomized clinical trial of a phytotherapic compound containing Pimpinella anisum, Foeniculum vulgare, Sambucus nigra, and Cassia augustifolia for chronic constipation. *BMC Complement Altern Med* 10. doi: 10.1186/1472-6882-10-17

Pieroni, A., Dibra, B., Grishaj, G., Grishaj, I., and Gjon Maçai, S. (2005). Traditional phytotherapy of the Albanians of Lepushe, Northern Albanian Alps. *Fitoterapia* 76, 379–399. doi: 10.1016/j.fitote.2005.03.015

Pokharel, Y. R., Kunwar, R. M., Bussmann, R. W., Paniagua-Zambrana, N. Y., and Abbasi, A. M. (2021). “Hippophae rhamnoides L. ssp. turkestanica Rousi Hippophae rhamnoides L. Hippophae salicifolia D. Don Hippophae tibetana Schltdl. Elaeagnaceae,” 1033–1041. doi: 10.1007/978-3-030-57408-6_117

Polumackanycz, M., Kaszuba, M., Konopacka, A., Marzec-Wróblewska, U., Wesolowski, M., Waleron, K., et al. (2020). Phenolic Composition and Biological Properties of Wild and Commercial Dog Rose Fruits and Leaves. *Molecules* 25. doi: 10.3390/MOLECULES25225272

Pop, R. M., Bocsan, I. C., Buzoianu, A. D., Chedea, V. S., Socaci, S. A., Pecoraro, M., et al. (2020). Evaluation of the Antioxidant Activity of Nigella sativa L. and Allium ursinum Extracts in a Cellular Model of Doxorubicin-Induced Cardiotoxicity. *Molecules* 25, 5259. doi: 10.3390/molecules25225259

Prangya Paramita Pati, Manas Ranjan Mishra, and Surya Narayan Das (2023). A Comparative Study between Cyproheptadine and Carum Carvi as Appetite Stimulant in Case of Weight Gain. *International Journal of Current Science Research and Review* 06, 1125–1127.

Prazina, N., Redzic, S., and Tuka, M. (2011). The use of wild medicinal plants in the traditional therapy of respiratory diseases in high mountain region of W. Balkan. *Planta Med* 77. doi: 10.1055/s-0031-1282392

Prvulovic, D., Popovic, M., Malencic, D., Ljubojevic, M., and Ognjanov, V. (2011). Phenolic compounds in sweet cherry (Prunus avium L.) petioles and their antioxidant properties. *Research Journal of Agricultural Science* 43, 198–202.

Puppala, E. R., Aochenlar, S. L., Shantanu, P. A., Ahmed, S., Jannu, A. K., Jala, A., et al. (2022). Perillyl alcohol attenuates chronic restraint stress aggravated dextran sulfate sodium-induced ulcerative colitis by modulating TLR4/NF-κB and JAK2/STAT3 signaling pathways. *Phytomedicine* 106, 154415. doi: 10.1016/J.PHYMED.2022.154415

Quave, C. L., Plano, L. R. W., Pantuso, T., and Bennett, B. C. (2008). Effects of extracts from Italian medicinal plants on planktonic growth, biofilm formation and adherence of methicillin-resistant Staphylococcus aureus. *J Ethnopharmacol* 118, 418–428. doi: 10.1016/J.JEP.2008.05.005

Radha, Kumar, M., Puri, S., Pundir, A., Bangar, S. P., Changan, S., et al. (2021). Evaluation of Nutritional, Phytochemical, and Mineral Composition of Selected Medicinal Plants for Therapeutic Uses from Cold Desert of Western Himalaya. *Plants 2021, Vol. 10, Page 1429* 10, 1429. doi: 10.3390/PLANTS10071429

Rakel, D. (2018). “Part I: Integrative Medicine,” in *Integrative Medicine*, (Elsevier), 1096–1123. doi: 10.1016/B978-0-323-35868-2.12001-8

Rasooli, I., and Mirmostafa, S. A. (2002). Antibacterial properties of Thymus pubescens and Thymus serpyllum essential oils. *Fitoterapia* 73, 244–250. doi: 10.1016/S0367-326X(02)00064-3

Rein, E., Kharazmi, A., and Winther, K. (2004). A herbal remedy, Hyben Vital (stand. powder of a subspecies of Rosa canina fruits), reduces pain and improves general wellbeing in patients with osteoarthritis--a double-blind, placebo-controlled, randomised trial. *Phytomedicine* 11, 383–391. doi: 10.1016/J.PHYMED.2004.01.001

Reiter, M., and Brandt, W. (1985). Relaxant effects on tracheal and ileal smooth muscles of the guinea pig. *Arzneimittelforschung* 35, 408–14.

Resmi, S., Fathima, L., and Vijayaraghavan, R. (2017). Formulation of a herbal extract for anemia treatment and its effect on physical work and intelligence capacity in adolescent girls with iron deficiency in India. *Afr J Pharm Pharmacol* 11, 284–288. doi: 10.5897/AJPP2017.4774

Roche, M. L., Ambato, L., Sarsoza, J., and Kuhnlein, H. V. (2017). Mothers’ groups enrich diet and culture through promoting traditional Quichua foods. *Matern Child Nutr* 13 Suppl 3. doi: 10.1111/MCN.12530

Röhrl, J., Jaklin, M., Piqué-Borràs, M.-R., Ammendola, A., and Künstle, G. (2022). Arnica planta tota is superior to Arnica flos in terms of anti-inflammatory properties in vitro and in vivo. doi: 10.1055/s-0042-1759296

Röhrl, J., Piqué-Borràs, M.-R., Jaklin, M., Werner, M., Werz, O., Josef, H., et al. (2023). Anti-Inflammatory Activities of Arnica montana Planta Tota versus Flower Extracts: Analytical, In Vitro and In Vivo Mouse Paw Oedema Model Studies. *Plants* 12, 1348. doi: 10.3390/plants12061348

Rovná, K., Ivanišová, E., Žiarovská, J., Ferus, P., Terentjeva, M., Kowalczewski, P. Ł., et al. (2020). Characterization of Rosa canina Fruits Collected in Urban Areas of Slovakia. Genome Size, iPBS Profiles and Antioxidant and Antimicrobial Activities. *Molecules* 25. doi: 10.3390/MOLECULES25081888

S, A., R, B., and P, A. (2011). Antihyperglycemic effect of Hypericum perforatum ethyl acetate extract on streptozotocin–induced diabetic rats. *Asian Pac J Trop Biomed* 1, 386–390. doi: 10.1016/S2221-1691(11)60085-3

Saaby, L., Moesby, L., Hansen, E. W., and Christensen, S. B. (2011). Isolation of immunomodulatory triterpene acids from a standardized rose hip powder (Rosa canina L.). *Phytother Res* 25, 195–201. doi: 10.1002/PTR.3241

Saaby, L., and Nielsen, C. H. (2012). Triterpene acids from rose hip powder inhibit self-antigen- and LPS-induced cytokine production and $CD4^+$ T-cell proliferation in human mononuclear cell cultures. *Phytother Res* 26, 1142–1147. doi: 10.1002/PTR.3713

Sabahi, Z., Hasan, S. M. F., Ayatollahi, S. A., Farmani, F., Afsari, A., and Moein, M. (2022). Improvement of Phenolic Compound Extraction by Using Ion Exchange Chromatography and Evaluation of Biological Activities of Polyphenol-enriched Fraction of Rosa canina Fruits. *Iran J Pharm Res* 21. doi: 10.5812/IJPR-126558

Sabzian-Molaei, F., Hosseini, S., Alipour, A., Ghaderi, H., Fotouhi-Chahouki, F., Hadi, A., et al. (2023). Urtica dioica agglutinin (UDA) as a potential candidate for inhibition of SARS-CoV-2 Omicron variants: In silico prediction and experimental validation. *Phytomedicine* 111, 154648. doi: 10.1016/J.PHYMED.2023.154648

Sabzian-Molaei, F., Khalili, M. A. N., Sabzian-Molaei, M., Shahsavarani, H., Pour, A. F., Rad, A. M., et al. (2022). Urtica dioica Agglutinin: A plant protein candidate for inhibition of SARS-COV-2 receptor-binding domain for control of Covid19 Infection. *PLoS One* 17, e0268156. doi: 10.1371/JOURNAL.PONE.0268156

Sahib, A., Mohammed, I., and Sloo, S. (2014). Antigiardial effect of <i>Anethum graveolens </i>aqueous extract in children. *J Intercult Ethnopharmacol* 3, 109. doi: 10.5455/jice.20140523104104

Said, A. A. H., Derfoufi, S., Sbai, I., and Benmoussa, A. (2015). Ethnopharmacological survey of traditional medicinal plants used for the treatment of infantile colic in Morocco. *J Chem Pharm Res* 7, 664–671.

Sakinah, M. (2021). Use of Herbal Medicinal Products among Children. *Eureka Herba Indonesia* 4, 156–159. doi: 10.37275/ehi.v4i1.29

Salah-Abbès, J. Ben, Abbès, S., Zohra, H., and Oueslati, R. (2015). Tunisian radish (Raphanus sativus) extract prevents cadmium-induced immunotoxic and biochemical alterations in rats. *J Immunotoxicol* 12, 40–47. doi: 10.3109/1547691X.2014.880534/SUPPL_FILE/IIMT_A_880534_SM8617.DOCX

Salaria, D., Rolta, R., Patel, C. N., Dev, K., Sourirajan, A., and Kumar, V. (2022). In vitro and in silico analysis of Thymus serpyllum essential oil as bioactivity enhancer of antibacterial and antifungal agents. *J Biomol Struct Dyn* 40, 10383–10402. doi: 10.1080/07391102.2021.1943530

Saleh, A. S., El-Newary, S. A., Mohamed, W. A., Elgamal, A. M., and Farah, M. A. (2024). Pumpkin seeds (Cucurbita pepo subsp. ovifera) decoction promotes Trichinella spiralis expulsion during intestinal phase via “Weep and Sweep” mechanism. *Sci Rep* 14, 1548. doi: 10.1038/s41598-024-51616-4

Sallustio, V., Chiocchio, I., Mandrone, M., Cirrincione, M., Protti, M., Farruggia, G., et al. (2022). Extraction, Encapsulation into Lipid Vesicular Systems, and Biological Activity of Rosa canina L. Bioactive Compounds for Dermocosmetic Use. *Molecules 2022, Vol. 27, Page 3025* 27, 3025. doi: 10.3390/MOLECULES27093025

Salman, S. S., and Ardalan, N. M. (2022). Evaluation of Amygdalin (B17) and Cucurbita pepo (Pumpkin seed) Activity Against Blastocystis from Diarrheic Patients in Baghdad, Iraq: in Vitro Study. *Baghdad Science Journal* 19, 0016. doi: 10.21123/bsj.2022.19.1.0016

Samadi, S., Khadivzadeh, T., Emami, A., Moosavi, N. S., Tafaghodi, M., and Behnam, H. R. (2010). The Effect of *Hypericum perforatum* on the Wound Healing and Scar of Cesarean. *The Journal of Alternative and Complementary Medicine* 16, 113–117. doi: 10.1089/acm.2009.0317

Sanguigno, L., Casamassa, A., Funel, N., Minale, M., Riccio, R., Riccio, S., et al. (2018). Triticum vulgare extract exerts an anti-inflammatory action in two in vitro models of inflammation in microglial cells. *PLoS One* 13. doi: 10.1371/JOURNAL.PONE.0197493

Sani, M. N., Kianifar, H. R., Kianee, A., and Khatami, G. (2006). Effect of oral garlic on arterial oxygen pressure in children with hepatopulmonary syndrome. *World J Gastroenterol* 12, 2427–31. doi: 10.3748/wjg.v12.i15.2427

Sargin, S. A., Akçicek, E., and Selvi, S. (2013). An ethnobotanical study of medicinal plants used by the local people of Alaşehir (Manisa) in Turkey. *J Ethnopharmacol* 150, 860–874. doi: 10.1016/J.JEP.2013.09.040

SARI, F., KÜLTÜR, Ş., and KOÇYİĞİT, M. (2023a). TRADITIONAL USAGES OF SOME MEDICINAL PLANTS FOR PEDIATRIC DISEASES. *Ankara Universitesi Eczacilik Fakultesi Dergisi* 47, 4–4. doi: 10.33483/jfpau.1214245

SARI, F., KÜLTÜR, Ş., and KOÇYİĞİT, M. (2023b). TRADITIONAL USAGES OF SOME MEDICINAL PLANTS FOR PEDIATRIC DISEASES. *Ankara Universitesi Eczacilik Fakultesi Dergisi* 47, 4–4. doi: 10.33483/jfpau.1214245

Sarrell, E. M., Cohen, H. A., and Kahan, E. (2003). Naturopathic treatment for ear pain in children. *Pediatrics* 111, e574–e579.

Sarrell, E. M., Mandelberg, A., and Cohen, H. A. (2001). Efficacy of naturopathic extracts in the management of ear pain associated with acute otitis media. *Arch Pediatr Adolesc Med* 155, 796–799. doi: 10.1001/archpedi.155.7.796

Sarwar, W., Ali, Q., and Ahmed, S. (2022). Microscopic visualization of the antibiofilm potential of essential oils against Staphylococcus aureus and Klebsiella pneumoniae. *Microsc Res Tech* 85, 3921–3931. doi: 10.1002/JEMT.24243

Savino, F., Cresi, F., Castagno, E., Silvestro, L., and Oggero, R. (2005). A randomized double-blind placebo-controlled trial of a standardized extract of Matricariae recutita, Foeniculum vulgare and Melissa officinalis (ColiMil) in the treatment of breastfed colicky infants. *Phytotherapy research* 19, 335–340. doi: 10.1002/ptr.1668

Schaal, B., Marlier, L., and Soussignan, R. (2000). Human Foetuses Learn Odours from their Pregnant Mother’s Diet. *Chem Senses* 25, 729–737. doi: 10.1093/CHEMSE/25.6.729

Schneider, F., Danski, M. T. R., and Vayego, S. A. (2015). Usage of Calendula officinalis in the prevention and treatment of radiodermatitis: a randomized double-blind controlled clinical trial. *Revista da Escola de Enfermagem da USP* 49, 0221–0228. doi: 10.1590/S0080-623420150000200006

Selected Evidence-Based Health Benefits of Topically Applied Sunflower Oil (2015). *Applied Science Reports* 10. doi: 10.15192/PSCP.ASR.2015.10.1.4549

Senol, F. S., Kan, A., Coksari, G., and Orhan, I. E. (2012). Antioxidant and anticholinesterase effects of frequently consumed cereal grains using in vitro test models. *Int J Food Sci Nutr* 63, 553–559. doi: 10.3109/09637486.2011.641943

Shahrajabian, M. H. (2021). Spear Thistle (Cirsium vulgare L.) and Ramsons (Allium ursinum L.), Impressive Health Benefits and High-Nutrient Medicinal Plants. *Pharmacognosy Communications* 11, 168–171.

Shahunja, K. M., Sévin, D. C., Kendall, L., Ahmed, T., Hossain, Md. I., Mahfuz, M., et al. (2021). Effect of topical applications of sunflower seed oil on systemic fatty acid levels in under-two children under rehabilitation for severe acute malnutrition in Bangladesh: a randomized controlled trial. *Nutr J* 20, 51. doi: 10.1186/s12937-021-00707-3

Shakeri, F., Amin, F., Marefati, N., Roshan, N. M., Boskabady, M., and Boskabady, M. H. (2023). Effect of Allium cepa extract on total and differential WBC, TP level, oxidant and antioxidant biomarkers, and lung pathology in ovalbumin-sensitized rats. *Allergol Immunopathol (Madr)* 51, 153–162. doi: 10.15586/aei.v51i3.532

Shakibaei, M., Allaway, D., Nebrich, S., and Mobasheri, A. (2012). Botanical extracts from rosehip (Rosa canina), willow bark (Salix alba), and nettle leaf (Urtica dioica) suppress IL-1β-induced NF-κB activation in canine articular chondrocytes. *Evidence-based Complementary and Alternative Medicine* 2012. doi: 10.1155/2012/509383

Shamsi, M., Ganji, A., Mosayebi, G., Amirhoseiny, E. S., Shohani, S., and Ghazavi, A. (2023). Chamomile and Urtica dioica extracts improve immunological and histological alterations associated with polycystic ovarian syndrome in DHEA -induced mice. *BMC Complement Med Ther* 23, 102. doi: 10.1186/S12906-023-03936-7/FIGURES/6

Shanaida, M., Hudz, N., Białoń, M., Kryvtsowa, M., Svydenko, L., Filipska, A., et al. (2021). Chromatographic profiles and antimicrobial activity of the essential oils obtained from some species and cultivars of the Mentheae tribe (Lamiaceae). *Saudi J Biol Sci* 28, 6145–6152. doi: 10.1016/J.SJBS.2021.06.068

Shaparenko, B. A., Slivko, A. B., Bazarova, O. V, Vishnevetskaia, E. N., and Selezneva, G. T. (1979). [Use of medicinal plants for the treatment of chronic suppurative otitis]. *Zh Ushn Nos Gorl Bolezn*, 48–51.

Sharif, M. R., Safari, A., Baghshahi, H., Akbari, H., Memarzadeh, M. R., MEHRAN, M., et al. (2024). THE EFFECT OF A THYME-IVY FLUID EXTRACT COMBINATION ON THE SEVERITY OF COUGH IN CHILDREN: RANDOMIZED CONTROLLED TRIAL. *New Armenian Medical Journal* 18.

Sharquie, K. E., and Al-Obaidi, H. K. (2002). Onion juice (Allium cepa L.), a new topical treatment for alopecia areata. *Journal of Dermatology* 29, 343–6. doi: 10.1111/j.1346-8138.2002.tb00277.x

Sheikh, A. A., Loveleen, L., Pant, K. K., and Chandra, V. (2022). Cherries: A Treasure Of Bioactive Compounds With Promising Health Promoting Functional Potential. *IJFANS International Journal of Food and Nutritional Sciences* 11.

Shekhawat, G., and Jana, S. (2010). Anethum graveolens: An Indian traditional medicinal herb and spice. *Pharmacogn Rev* 4, 179. doi: 10.4103/0973-7847.70915

Sherkatolabbasieh, H., Bahmani, M., and Hamidi, M. (2021). Common treatments with indigenous Iranian plants for routine pediatric diseases and disorders. *International Journal of Biology and Chemistry* 14. doi: 10.26577/ijbch.2021.v14.i1.09

Shulman, R. J., Chumpitazi, B. P., Abdel-Rahman, S. M., Garg, U., Musaad, S., and Kearns, G. L. (2022). Randomised trial: Peppermint oil (menthol) pharmacokinetics in children and effects on gut motility in children with functional abdominal pain. *Br J Clin Pharmacol* 88, 1321–1333. doi: 10.1111/BCP.15076

Silverberg, N. B. (2002). Garlic cloves for verruca vulgaris. *Pediatr Dermatol* 19, 183. doi: 10.1046/j.1525-1470.2002.00038.x
Singh, H., and Khar, A. (2022). Potential of onion (Allium cepa) as traditional therapeutic and functional food: An update. *The Indian Journal of Agricultural Sciences* 92. doi: 10.56093/ijas.v92i11.123235
Singh, K. G., Sonia, S., and Konsoor, N. (2018). IN-VITRO AND EX-VIVO STUDIES ON THE ANTIOXIDANT, ANTI-INFLAMMATORY AND ANTIARTHRITIC PROPERTIES OF CAMELLIA SINENSIS, HIBISCUS ROSA SINENSIS, MATRICARIA CHAMOMILLA, ROSA SP., ZINGIBER OFFICINALE TEA EXTRACTS. *Int J Pharm Sci Res* 9, 3543. doi: 10.13040/IJPSR.0975-8232.9(8).3543-51
Singh, K., Pannu, M. S., Singh, P., and Singh, J. (2010). Effect of wheat grass tablets on the frequency of blood transfusions in Thalassemia Major. *Indian J Pediatr* 77, 90–91. doi: 10.1007/S12098-010-0002-8/METRICS
Sipos, P., Hagymási, K., Lugasi, A., Fehér, E., and Blázovics, A. (2002). Effects of black radish root (Raphanus sativus L. var niger) on the colon mucosa in rats fed a fat rich diet. *Phytotherapy Research* 16, 677–679. doi: 10.1002/PTR.950
Slimestad, R., Johny, A., Thomsen, M. G., Karlsen, C. R., and Rosnes, J. T. (2022). Chemical Profiling and Biological Activity of Extracts from Nine Norwegian Medicinal and Aromatic Plants. *Molecules 2022, Vol. 27, Page 7335* 27, 7335. doi: 10.3390/MOLECULES27217335
Smeets, K., Van Damme*, E. J. M., Van Leuven, F., and Peumans, W. J. (1997). Isolation, characterization and molecular cloning of a leaf-specific lectin from ramsons (Allium ursinum L.). *Plant Mol Biol* 35, 531–535. doi: 10.1023/A:1005887016694
Sobolewska, D., Janeczko, Z., Kisiel, W., Podolak, I., Galanty, A., and Trojanowska, D. (2006). Steroidal glycosides from the underground parts of Allium ursinum L. and their cytostatic and antimicrobial activity. *Acta Pol Pharm* 63, 219–23.
Soffar, S. A., and Mokhtar, G. M. (1991). Evaluation of the antiparasitic effect of aqueous garlic (Allium sativum) extract in hymenolepiasis nana and giardiasis. *J Egypt Soc Parasitol* 21, 497–502.
Sorme, F. M., Tabarrai, M., Alimadady, H., Rahimi, R., Sepidarkish, M., and Karimi, M. (2019). Efficacy of Matricaria chamomilla L. in Infantile Colic: A Double Blind, Placebo Controlled Randomized Trial. *J Pharm Res Int* 31, 1–11. doi: 10.9734/JPRI/2019/V31I630385
Soualeh, N., Stiévenard, A., Baudelaire, E., Soulimani, R., and Bouayed, J. (2017). Improvement of cytoprotective and antioxidant activity of Rosa canina L. and Salix alba L. by controlled differential sieving process against H2O2-induced oxidative stress in mouse primary splenocytes. *Int J Vitam Nutr Res* 87, 191–200. doi: 10.1024/0300-9831/A000506
Sõukand, R. (2016). Perceived reasons for changes in the use of wild food plants in Saaremaa, Estonia. *Appetite* 107, 231–241. doi: 10.1016/j.appet.2016.08.011
Sõukand, R., and Kalle, R. (2013). Where does the border lie: locally grown plants used for making tea for recreation and/or healing, 1970s-1990s Estonia. *J Ethnopharmacol* 150, 162–174. doi: 10.1016/J.JEP.2013.08.031

Soulimani, R., Dicko, A., Baudelaire, E., and Bouayed, J. (2021). Increased anti-inflammatory activity and enhanced phytochemical concentrations in superfine powders obtained by controlled differential sieving process from four medicinal plants. *Int J Vitam Nutr Res*. doi: 10.1024/0300-9831/A000739

Spaggiari, C., Annunziato, G., Spadini, C., Montanaro, S. L., Iannarelli, M., Cabassi, C. S., et al. (2023). Extraction and Quantification of Azelaic Acid from Different Wheat Samples (Triticum durum Desf.) and Evaluation of Their Antimicrobial and Antioxidant Activities. *Molecules* 28, 2134. doi: 10.3390/MOLECULES28052134/S1

Stanisavljević, N., Soković Bajić, S., Jovanović, Ž., Matić, I., Tolinački, M., Popović, D., et al. (2020). Antioxidant and Antiproliferative Activity of Allium ursinum and Their Associated Microbiota During Simulated in vitro Digestion in the Presence of Food Matrix. *Front Microbiol* 11. doi: 10.3389/fmicb.2020.601616

Sterling, J. C., Gibbs, S., Haque Hussain, S. S., Mohd Mustapa, M. F., Handfield-Jones, S. E., Hughes, J. R., et al. (2014). British Association of Dermatologists' guidelines for the management of cutaneous warts 2014. *British Journal of Dermatology* 171, 696–712. doi: 10.1111/bjd.13310

Stinging Nettle (2021). *Drugs and Lactation Database (LactMed®)*. Available at: https://www.ncbi.nlm.nih.gov/books/NBK501777/ (Accessed June 8, 2023).

Su, Y. H., and Lin, J. Y. (2022). Menthone Inhalation Alleviates Local and Systemic Allergic Inflammation in Ovalbumin-Sensitized and Challenged Asthmatic Mice. *Int J Mol Sci* 23. doi: 10.3390/IJMS23074011

Sui, H., Wang, F., Weng, Z., Song, H., Fang, Y., Tang, X., et al. (2020). A wheat germ-derived peptide YDWPGGRN facilitates skin wound-healing processes. *Biochem Biophys Res Commun* 524, 943–950. doi: 10.1016/J.BBRC.2020.01.162

Sümeli, R., Cömert, H. S. Y., Sarıhan, H., İmamoğlu, M., and Saygın, İ. (2022). Effectiveness of *Hypericum perforatum* Extract in the Treatment of Corrosive Esophageal Burns. *Journal of Investigative Surgery* 35, 647–652. doi: 10.1080/08941939.2021.1921083

Summers, A., Visscher, M. O., Khatry, S. K., Sherchand, J. B., LeClerq, S. C., Katz, J., et al. (2019). Impact of sunflower seed oil versus mustard seed oil on skin barrier function in newborns: a community-based, cluster-randomized trial. *BMC Pediatr* 19, 512. doi: 10.1186/s12887-019-1871-2

Sun, Z., Wang, H., Wang, J., Zhou, L., and Yang, P. (2014). Chemical Composition and Anti-Inflammatory, Cytotoxic and Antioxidant Activities of Essential Oil from Leaves of Mentha piperita Grown in China. *PLoS One* 9. doi: 10.1371/JOURNAL.PONE.0114767

Suthutvoravut, U., Tontisirin, K., Varavithya, W., Valyasevi, A., Björck, I., and Dahlqvist, A. (1984). Wheat extract and milk mixture as a milk substitute for children with milk intolerance. *J Diarrhoeal Dis Res* 2, 168–172. Available at: https://pubmed.ncbi.nlm.nih.gov/6512217/ (Accessed June 9, 2023).

Takahashi, M., and Shibamoto, T. (2008). Chemical Compositions and Antioxidant/Anti-inflammatory Activities of Steam Distillate from Freeze-Dried Onion (Allium cepa L.) Sprout. *J Agric Food Chem* 56, 10462–10467. doi: 10.1021/jf801220b

Tan, H., Wu, G., Li, X., and Hou, W. (2020). Suggestion of an alternative approach of inhalation of volatile chemicals from onion and garlic for isolated patient of mild onset infected flu: review and communication.

Tas, A. (2009). Analgesic effect of Pimpinella anisum L. essential oil extract in mice. *Indian Veterinary Journal* 86, 145–147.

Tavassoli, S., Eftekhari, K., Karimi, M., Ghobadi, A., Shati, M., Naddaf, A., et al. (2021a). Effectiveness of Viola Flower Syrup Compared with Polyethylene Glycol in Children with Functional Constipation: A Randomized, Active-Controlled Clinical Trial. *Evidence-Based Complementary and Alternative Medicine* 2021, 1–8. doi: 10.1155/2021/9915289

Tavassoli, S., Eftekhari, K., Karimi, M., Ghobadi, A., Shati, M., Naddaf, A., et al. (2021b). Effectiveness of Viola Flower Syrup Compared with Polyethylene Glycol in Children with Functional Constipation: A Randomized, Active-Controlled Clinical Trial. *Evidence-Based Complementary and Alternative Medicine* 2021, 1–8. doi: 10.1155/2021/9915289

Tayefi-Nasrabadi, H., Sadigh-Eteghad, S., and Aghdam, Z. (2012). The Effects of the Hydroalcohol Extract of Rosa canina L. Fruit on Experimentally Nephrolithiasic Wistar Rats. *Phytotherapy Research* 26, 78–85. doi: 10.1002/PTR.3519

Thapa, S., Luna, R. A., Chumpitazi, B. P., Oezguen, N., Abdel-Rahman, S. M., Garg, U., et al. (2022). Peppermint oil effects on the gut microbiome in children with functional abdominal pain. *Clin Transl Sci* 15, 1036–1049. doi: 10.1111/CTS.13224

The first newborn patient with SARS-CoV-2 variant B.1.1.7 identified in Viet Nam: treatment and care practices: Newborn with B1.1.7 variant in Viet Nam | Western Pacific Surveillance and Response (n.d.). Available at: https://ojs.wpro.who.int/ojs/index.php/wpsar/article/view/870 (Accessed June 21, 2023).

Thomas, A., Thakur, S., and Habib, R. (2017). Comparison of Antimicrobial Efficacy of Green Tea, Garlic with Lime, and Sodium Fluoride Mouth Rinses against Streptococcus mutans, Lactobacilli species, and Candida albicans in Children: A Randomized Double-blind Controlled Clinical Trial. *Int J Clin Pediatr Dent* 10, 234–239. doi: 10.5005/jp-journals-10005-1442

Thompson Coon, J., and Ernst, E. (2002). Herbal medicinal products for non-ulcer dyspepsia. *Aliment Pharmacol Ther* 16, 1689–1699. doi: 10.1046/j.1365-2036.2002.01339.x

Thompson, E. A., Bishop, J. L., and Northstone, K. (2010a). The Use of Homeopathic Products in Childhood: Data Generated over 8.5 Years from the Avon Longitudinal Study of Parents and Children (ALSPAC). *The Journal of Alternative and Complementary Medicine* 16, 69–79. doi: 10.1089/acm.2009.0007

Thompson, E. A., Bishop, J. L., and Northstone, K. (2010b). The use of homeopathic products in childhood: data generated over 8.5 years from the Avon Longitudinal Study of Parents and Children (ALSPAC). *The Journal of Alternative and Complementary Medicine* 16, 69–79.

Timoshenko, A. V., Kayser, K., Kaltner, H., André, S., and Gabius, H. J. (1996). Binding capacities of two immunomodulatory lectins, carrier-immobilized glycoligands and steroid hormones in lung cancer and the concentration of nitrite/nitrate in pleural effusions. *Lung Cancer* 14, 75–84. doi: 10.1016/0169-5002(95)00513-7

Tirapelli, C. R., de Andrade, C. R., Cassano, A. O., De Souza, F. A., Ambrosio, S. R., da Costa, F. B., et al. (2007). Antispasmodic and relaxant effects of the hidroalcoholic extract of Pimpinella anisum (Apiaceae) on rat anococcygeus smooth muscle. *J Ethnopharmacol* 110, 23–29. doi: 10.1016/J.JEP.2006.08.031

Tobyn, G., Denham, A., and Whitelegg, M. (2011). “Some observations on the Western herbal tradition,” in *Medical Herbs*, (Elsevier), 23–28. doi: 10.1016/B978-0-443-10344-5.00007-0

Toiu, A., Pârvu, A. E., Oniga, I., and Tămaş, M. (2007). Evaluation of anti-inflammatory activity of alcoholic extract from Viola tricolor. *Rev Med Chir Soc Med Nat Iasi* 111, 525–9.

Tsioutsiou, E. E., Giordani, P., Hanlidou, E., Biagi, M., De Feo, V., and Cornara, L. (2019). Ethnobotanical Study of Medicinal Plants Used in Central Macedonia, Greece. *Evidence-based Complementary and Alternative Medicine* 2019. doi: 10.1155/2019/4513792

Tullio, V., Roana, J., Cavallo, L., and Mandras, N. (2023). Immune Defences: A View from the Side of the Essential Oils. *Molecules 2023, Vol. 28, Page 435* 28, 435. doi: 10.3390/MOLECULES28010435

Tullio, V., Roana, J., Scalas, D., and Mandras, N. (2019). Evaluation of the Antifungal Activity of Mentha x piperita (Lamiaceae) of Pancalieri (Turin, Italy) Essential Oil and Its Synergistic Interaction with Azoles. *Molecules 2019, Vol. 24, Page 3148* 24, 3148. doi: 10.3390/MOLECULES24173148

Tumbas, V. T., Čanadanović-Brunet, J. M., Četojević-Simin, D. D., Ćetković, G. S., Dilas, S. M., and Gille, L. (2012). Effect of rosehip (Rosa canina L.) phytochemicals on stable free radicals and human cancer cells. *J Sci Food Agric* 92, 1273–1281. doi: 10.1002/JSFA.4695

Tural Büyük E, Güdek E, and Kalaycı N. (2014). Approaches of mothers to children with cough. *Gümüşhane Univ J Health Sci.* 3, 1019–1031.

Turrini, F., Vallarino, G., Cisani, F., Donno, D., Beccaro, G. L., Zunin, P., et al. (2020). Use of an Animal Model to Evaluate Anxiolytic Effects of Dietary Supplementation with Tilia tomentosa Moench Bud Extracts. *Nutrients 2020, Vol. 12, Page 3328* 12, 3328. doi: 10.3390/NU12113328

Uğur, Y., and Güzel, A. (2023). Determination of phytochemical content by LC-MS/MS, investigation of antioxidant capacity, and enzyme inhibition effects of nettle (Urtica dioica). *Eur Rev Med Pharmacol Sci* 27, 1793–1800. doi: 10.26355/EURREV_202303_31540

Urban, J., Kokoska, L., Langrova, I., and Matejkova, J. (2008a). *In Vitro* Anthelmintic Effects of Medicinal Plants Used in Czech Republic. *Pharm Biol* 46, 808–813. doi: 10.1080/13880200802315618
Urban, J., Kokoska, L., Langrova, I., and Matejkova, J. (2008b). *In Vitro* Anthelmintic Effects of Medicinal Plants Used in Czech Republic. *Pharm Biol* 46, 808–813. doi: 10.1080/13880200802315618
Valková, V., Ďúranová, H., Vukovic, N. L., Vukic, M., Kluz, M., and Kačániová, M. (2022). Assessment of Chemical Composition and Anti-Penicillium Activity of Vapours of Essential Oils from Abies Alba and Two Melaleuca Species in Food Model Systems. *Molecules* 27. doi: 10.3390/molecules27103101
Van Tilburg, M. A. L., and Felix, C. T. (2013). Diet and functional abdominal pain in children and adolescents. *J Pediatr Gastroenterol Nutr* 57, 141–148. doi: 10.1097/MPG.0B013E31829AE5C5
Vinci, G., Prencipe, S. A., Armeli, F., and Businaro, R. (2023). A Multimethodological Approach for the Valorization of “Senatore Cappelli” Wheat Milling By-Products as a Source of Bioactive Compounds and Nutraceutical Activity. *International Journal of Environmental Research and Public Health 2023, Vol. 20, Page 5057* 20, 5057. doi: 10.3390/IJERPH20065057
Viola, H., Wolfman, C., de Stein, M. L., Wasowski, C., Peña, C., Medina, J. H., et al. (1994). Isolation of pharmacologically active benzodiazepine receptor ligands from Tilia tomentosa (Tiliaceae). *J Ethnopharmacol* 44, 47–53. doi: 10.1016/0378-8741(94)90098-1
Vukics, V., Kery, A., Bonn, G. K., and Guttman, A. (2008). Major flavonoid components of heartsease (Viola tricolor L.) and their antioxidant activities. *Anal Bioanal Chem* 390, 1917–1925. doi: 10.1007/s00216-008-1885-3
Wali, R., Khan, M. F., Mahmood, A., Mahmood, M., Qureshi, R., Ahmad, K. S., et al. (2022). Ethnomedicinal appraisal of plants used for the treatment of gastrointestinal complaints by tribal communities living in Diamir district, Western Himalayas, Pakistan. *PLoS One* 17, e0269445. doi: 10.1371/JOURNAL.PONE.0269445
Wanes, D., Toutounji, M., Sebai, H., Rizk, S., and Naim, H. Y. (2021). Rosa canina L. Can Restore Endoplasmic Reticulum Alterations, Protein Trafficking and Membrane Integrity in a Dextran Sulfate Sodium-Induced Inflammatory Bowel Disease Phenotype. *Nutrients 2021, Vol. 13, Page 441* 13, 441. doi: 10.3390/NU13020441
Warholm, O., Skaar, S., Hedman, E., Mølmen, H. M., and Eik, L. (2003). The Effects of a Standardized Herbal Remedy Made from a Subtype of Rosa canina in Patients with Osteoarthritis: A Double-Blind, Randomized, Placebo-Controlled Clinical Trial. *Curr Ther Res Clin Exp* 64, 21–31. doi: 10.1016/S0011-393X(03)00004-3
Weber, L., Kuck, K., Jürgenliemk, G., Heilmann, J., Lipowicz, B., and Vissiennon, C. (2020). Anti-Inflammatory and Barrier-Stabilising Effects of Myrrh, Coffee Charcoal and Chamomile Flower Extract in a Co-Culture Cell Model of the Intestinal Mucosa. *Biomolecules 2020, Vol. 10, Page 1033* 10, 1033. doi: 10.3390/BIOM10071033

Weber, W., Vander Stoep, A., McCarty, R. L., Weiss, N. S., Biederman, J., and McClellan, J. (2008). Hypericum perforatum (St John’s Wort) for Attention-Deficit/Hyperactivity Disorder in Children and Adolescents. *JAMA* 299, 2633. doi: 10.1001/jama.299.22.2633

Wenzig, E. M., Widowitz, U., Kunert, O., Chrubasik, S., Bucar, F., Knauder, E., et al. (2008). Phytochemical composition and in vitro pharmacological activity of two rose hip (Rosa canina L.) preparations. *Phytomedicine* 15, 826–835. doi: 10.1016/J.PHYMED.2008.06.012

Whent, M., Huang, H., Xie, Z., Lutterodt, H., Yue, L., Fuerst, E. P., et al. (2012). Phytochemical Composition, Anti-inflammatory, and Antiproliferative Activity of Whole Wheat Flour. *J Agric Food Chem* 60, 2129–2135. doi: 10.1021/JF203807W

Willich, S. N., Rossnagel, K., Roll, S., Wagner, A., Mune, O., Erlendson, J., et al. (2010). Rose hip herbal remedy in patients with rheumatoid arthritis – a randomised controlled trial. *Phytomedicine* 17, 87–93. doi: 10.1016/J.PHYMED.2009.09.003

Wińska, K., Mączka, W., Łyczko, J., Grabarczyk, M., Czubaszek, A., and Szumny, A. (2019). Essential Oils as Antimicrobial Agents—Myth or Real Alternative? *Molecules 2019, Vol. 24, Page 2130* 24, 2130. doi: 10.3390/MOLECULES24112130

Winther, K., Apel, K., and Thamsborg, G. (2005). A powder made from seeds and shells of a rose-hip subspecies (Rosa canina) reduces symptoms of knee and hip osteoarthritis: a randomized, double-blind, placebo-controlled clinical trial. *Scand J Rheumatol* 34, 302–308. doi: 10.1080/03009740510018624

Witkowska-Banaszczak, E., Bylka, W., Matławska, I., Goślińska, O., and Muszyński, Z. (2005a). Antimicrobial activity of Viola tricolor herb. *Fitoterapia* 76, 458–461. doi: 10.1016/j.fitote.2005.03.005

Witkowska-Banaszczak, E., Bylka, W., Matławska, I., Goślińska, O., and Muszyński, Z. (2005b). Antimicrobial activity of Viola tricolor herb. *Fitoterapia* 76, 458–461. doi: 10.1016/j.fitote.2005.03.005

Xiao, M., Qiu, X., Yue, D., Cai, Y., and Mo, Q. (2013). Influence of hippophae rhamnoides on two appetite factors, gastric emptying and metabolic parameters, in children with functional dyspepsia. *Hell J Nucl Med* 16, 38–43. doi: 10.1967/s002449910070

YIN, Z. N., WU, W. J., SUN, C. Z., LIU, H. F., CHEN, W. B., ZHAN, Q. P., et al. (2019). Antioxidant and Anti-inflammatory Capacity of Ferulic Acid Released from Wheat Bran by Solid-state Fermentation of Aspergillus niger. *Biomedical and Environmental Sciences* 32, 11–21. doi: 10.3967/BES2019.002

Yousefi, S. S., Sadeghpour, O., Hamzehgardeshi, Z., and Sohrabvand, F. (2019). The effects of Carum carvi (Bunium persicum Boiss) on early return of bowel motility after caesarean section: Double-blind, randomized, placebo-controlled trial. *J Family Reprod Health* 13, 35.

Zahid, R., Asif, H. M., Rasheed, F., Rashid, F., Kamran, R., Ahmed, K., et al. (2020). In vitro and in vivo anti-Helicobacter pylori activity of selected medicinal plants employed for the management of gastrointestinal disorders. *Pak J Pharm Sci* 33, 2809–2814.

Zaia, M. G., Cagnazzo, T. di O., Feitosa, K. A., Soares, E. G., Faccioli, L. H., Allegretti, S. M., et al. (2016). Anti-Inflammatory Properties of Menthol and Menthone in Schistosoma mansoni Infection. *Front Pharmacol* 7. doi: 10.3389/FPHAR.2016.00170

Zardast, M., Namakin, K., Esmaelian Kaho, J., and Hashemi, S. S. (2016). Assessment of antibacterial effect of garlic in patients infected with Helicobacter pylori using urease breath test. *Avicenna J Phytomed* 6, 495–501.

Zargar, S., Wani, T. A., and Rizwan Ahamad, S. (2023). An Insight into Wheat Germ Oil Nutrition, Identification of Its Bioactive Constituents and Computer-Aided Multidimensional Data Analysis of Its Potential Anti-Inflammatory Effect via Molecular Connections. *Life (Basel)* 13. doi: 10.3390/LIFE13020526

Zargari, A. (1997). *Medicinal Plants*., ed. 1997 Tehran Univ. Tehran: Tehran University publications.

Zdunić, G., Gođevac, D., Milenković, M., Vučićević, D., Šavikin, K., Menković, N., et al. (2009). Evaluation of *Hypericum perforatum* oil extracts for an antiinflammatory and gastroprotective activity in rats. *Phytotherapy Research* 23, 1559–1564. doi: 10.1002/ptr.2809

Ziarlarimi, A., Irani, M., Gharahveysi, S., and Rahmani, Z. (2011). Investigation of antibacterial effects of garlic (Allium sativum), mint (Menthe spp.) and onion (Allium cepa) herbal extracts on Escherichia coli isolated from broiler chickens. *Afr J Biotechnol* 10, 10320–10322.

Zielińska, S., Jezierska-Domaradzka, A., Wójciak-Kosior, M., Sowa, I., Junka, A., and Matkowski, A. M. (2018). Greater Celandine’s Ups and Downs−21 Centuries of Medicinal Uses of Chelidonium majus From the Viewpoint of Today’s Pharmacology. *Front Pharmacol* 9. doi: 10.3389/fphar.2018.00299

Živković, J., Stojković, D., Petrović, J., Zdunić, G., Glamočlija, J., and Soković, M. (2015). Rosa canina L.--new possibilities for an old medicinal herb. *Food Funct* 6, 3687–3692. doi: 10.1039/C5FO00820D

Zou, Y., Lu, Y., and Wei, D. (2010). Protective effects of a flavonoid-rich extract of *Hypericum perforatum* L. against hydrogen peroxide-induced apoptosis in PC12 cells. *Phytotherapy Research* 24. doi: 10.1002/ptr.2852
